# Supplementary material for: Astrocytic GABA controls fidelity of temporal cortical processing in Fragile X Syndrome
Source: Neurobiol Dis. Author manuscript; Available in PMC 2026 Jul 15. (PMC13371466; doi:10.1016/j.nbd.2025.107233)

**Supplementary Information**

**Astrocytic GABA controls fidelity of temporal cortical processing in Fragile X Syndrome**

Victoria A. Wagner, Ritika Thapa, Anna O. Norman, Alexandra Varallo, Jordan Wimberly, Maham Rais, Dmytro Gerasymchuk, Timo P. Piepponen, Khaleel A. Razak, Maija Castren, Iryna M. Ethell


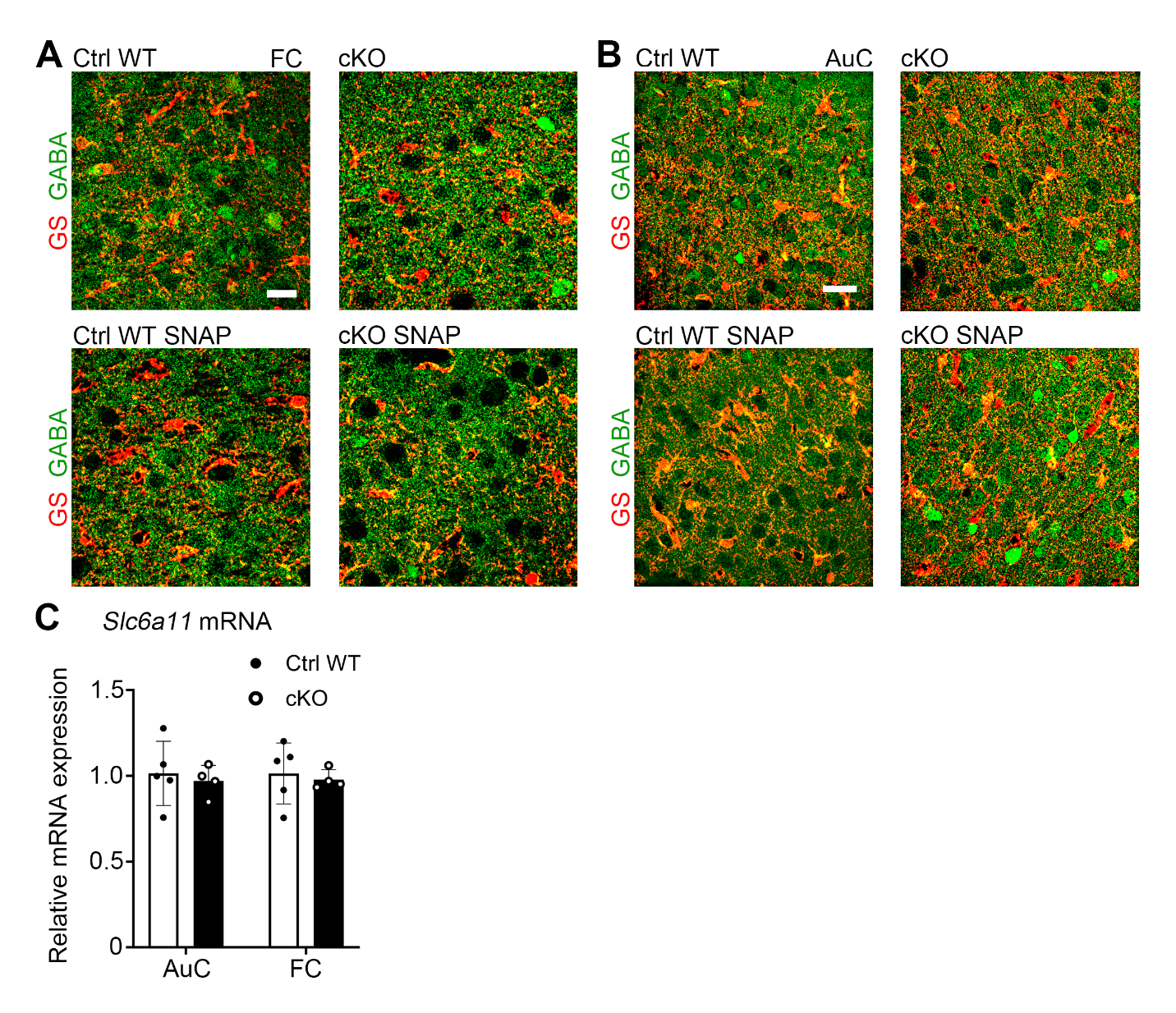


**Figure S1: Astrocyte-specific Fmr1 deletion alters GABA levels in astrocytes but does not affect expression of mRNA encoding GAT3, related to Figure 3**

A-B, Confocal images showing Glutamine Synthetase (GS, red) and GABA (green) immunoreactivity in superficial layers of FC (A) and AuC (B) of vehicle-treated and SNAP-treated Ctrl WT and cKO mice. Scale bar 20 µm for A, 30 µm for B. C, Quantitative analysis of *Slc6a11* mRNA performed with qPCR. Graph shows mean ± SD (n=4-5 mice/group; p=0.05; t-test). We found no significant differences in *Slc6a11* expression in cKO mice compared to Ctrl WT.


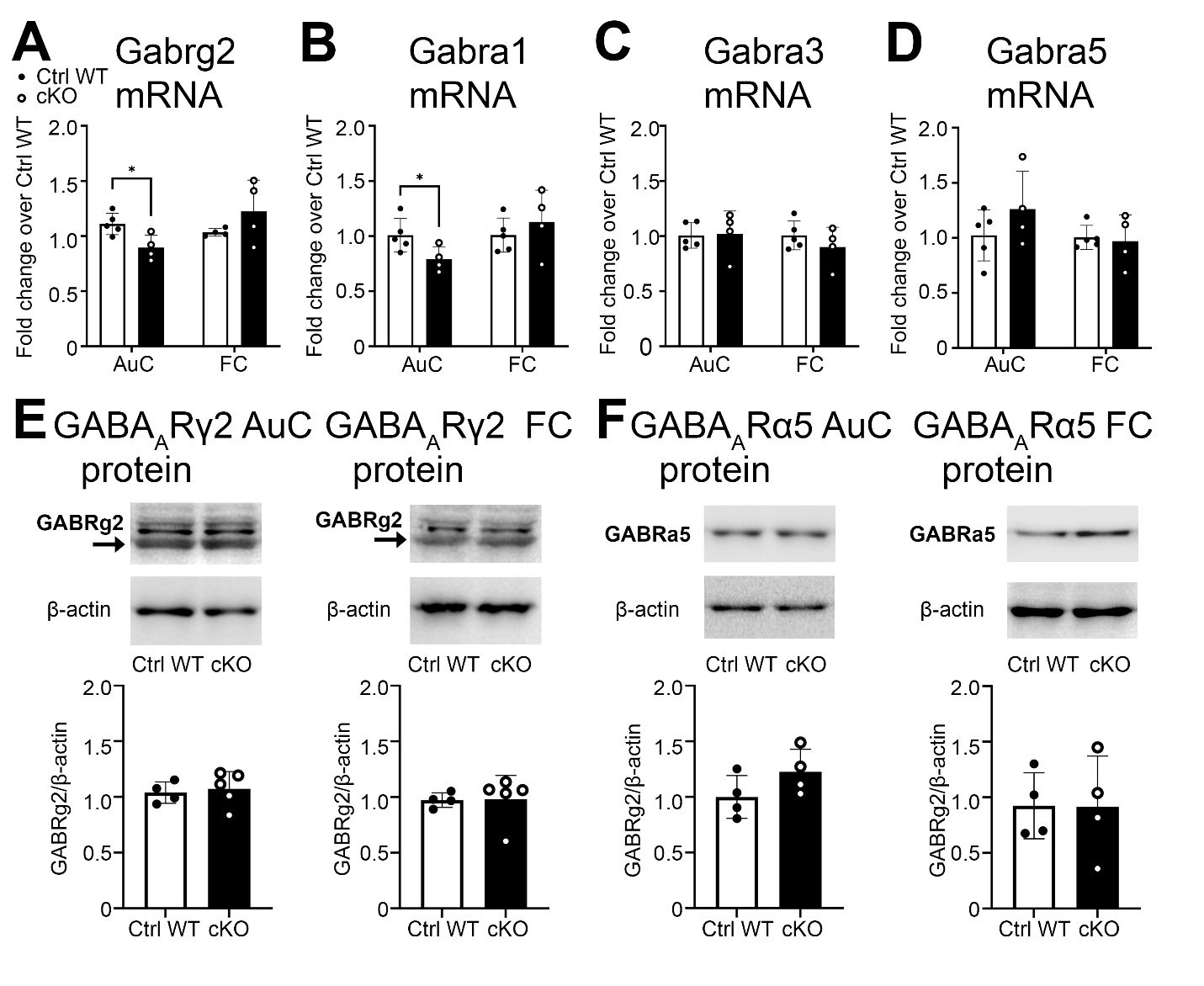


**Figure S2: Effects of astrocyte-specific Fmr1 deletion on mRNA expression and protein levels of GABAA receptor subunits, related to Figure 4**

A-D, Quantitative analysis of mRNA encoding GABA_A_ receptor subunits performed with qPCR. Graphs show mean ± SD (n=4-5 mice/group; *p<0.05; **p<0.01; t-test). There is a significant increase in mRNA levels of *Gabrg2* (A) and *Gabra1* (B) in the frontal cortex of cKO mice compared to Ctrl WT.  No significant differences were observed in mRNA levels of *Gabra3* (C) and *Gabra5* (D). E-F, Western blots showing GABAARγ2 (E, arrows indicate the measured band), GABAARα5 (F), and beta-actin protein levels in lysates from auditory cortex (left) and frontal cortex (right) of Ctrl WT and cKO mice. Graphs show mean ± SD (n=3-4/group, p=0.05; t-test). No differences were observed in GABAARγ2 or GABAARα5 protein levels.

**
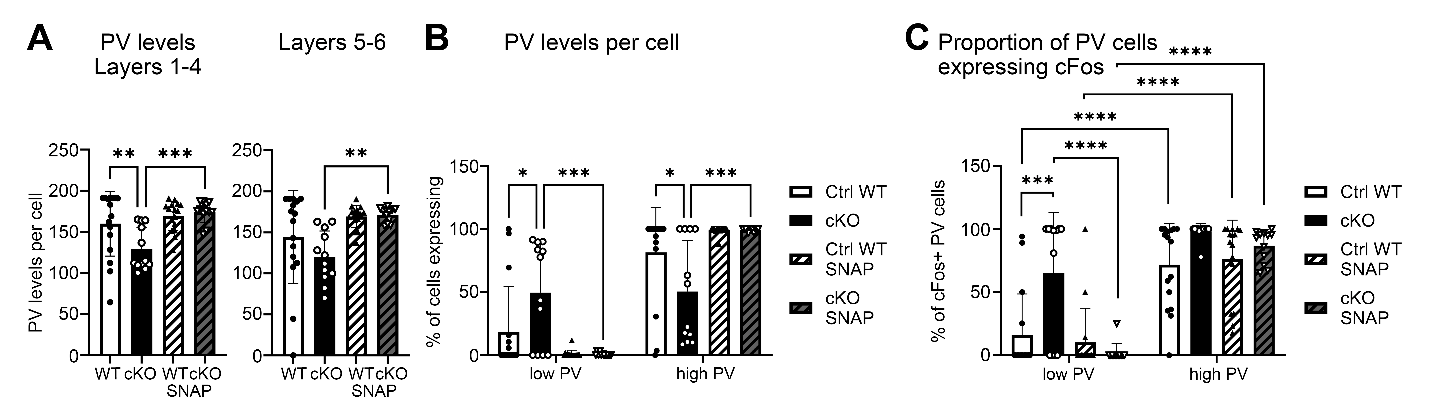
**

**Figure S3: Parvalbumin levels are significantly reduced in superficial layers in AuC of astrocyte-specific cKO mice and enhanced following acute blockage of GAT3-mediated astrocytic GABA transport, related to Figure 5**

A, Quantitative analysis of average PV immunoreactivity per cell in L1-4 (left) and L5/6 (right) AuC of Ctrl WT, cKO, Ctrl WT SNAP, and cKO SNAP groups. Graph shows mean ± SD (n= 12-16 images/group, **p < 0.01; ***p < 0.001; two-way ANOVA, Bonferroni's multiple comparisons test). PV levels are significantly reduced in L1-4 of cKO mice and are trending towards significance in L5/6. SNAP treatment significantly increases PV in L1-4 and L5/6 of cKO. B, Proportion of low expressing PV cells and high expressing PV cells in L1-6 AuC of Ctrl WT, cKO, Ctrl WT SNAP, and cKO SNAP groups. Graph shows mean ± SD (n= 12-16 images/group, *p<0.05; ***p < 0.001; two-way ANOVA, Tukey's multiple comparisons test). cKO mice have a significantly higher proportion of low PV cells than Ctrl WT. SNAP treatment significantly increased proportion of high PV cells in cKO. C, Proportion of low and high PV cells positive for cFos immunolabeling in AuC of Ctrl WT, cKO, Ctrl WT SNAP, and cKO SNAP groups. Graph shows mean ± SD (n= 12-16 images/group, ***p < 0.001; ****p<0.0001; three-way ANOVA, Bonferroni's multiple comparisons test). Proportion of cFos positive PV cells is greater for high expressing PV cells than low expressing PV cells for Ctrl WT but not cKO, and cFos/PV ratio is normalized in cKO following SNAP treatment.
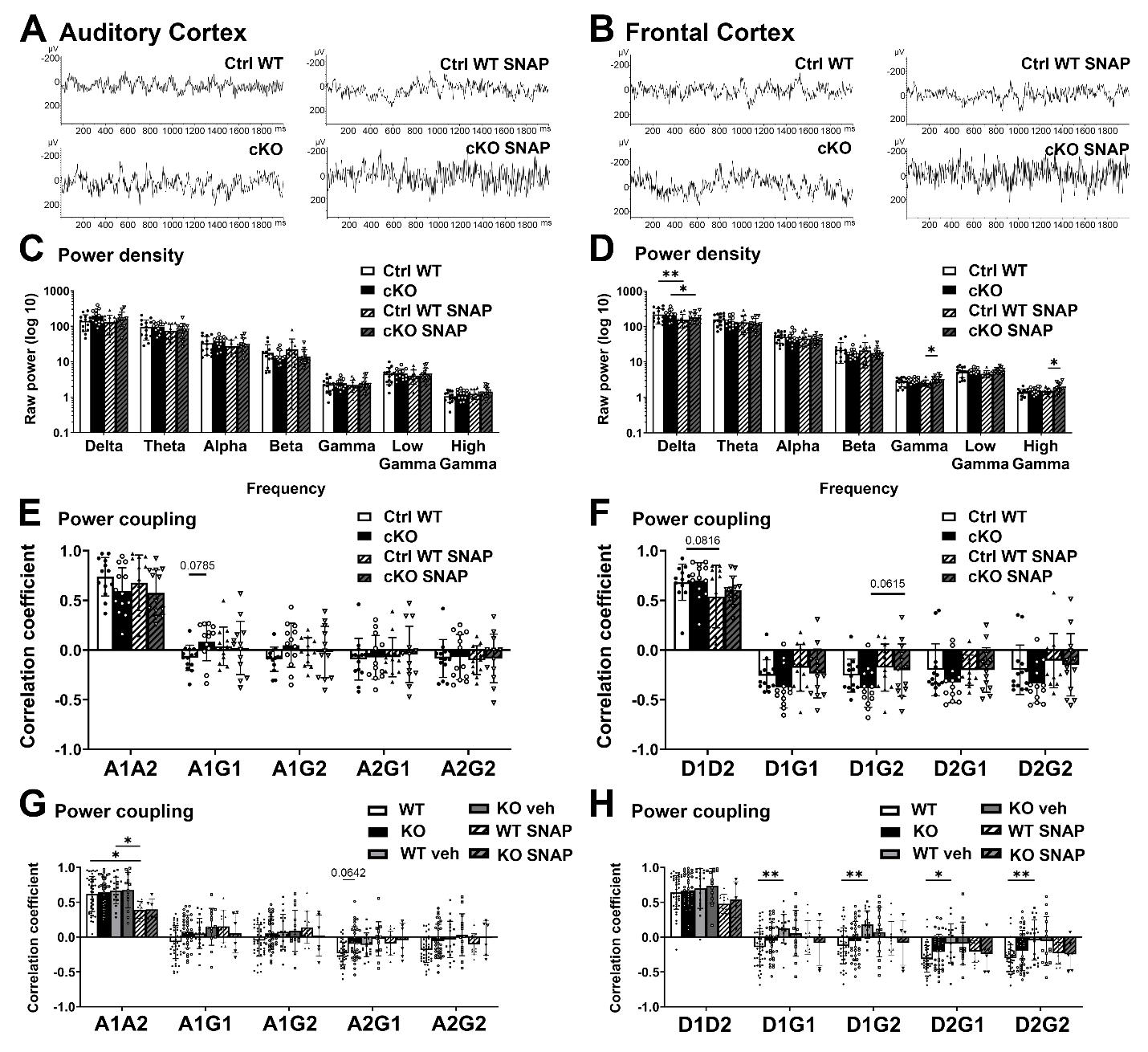
**Figure S4 Acute blockade of GAT3-mediated astrocytic GABA transport enhances baseline EEG power of gamma oscillations in FC**

A-B, Examples of representative EEG traces (in the absence of auditory stimulation) from electrodes implanted in AuC (A) and FC (B) in Ctrl WT (n=14) and cKO (n=13) mice before and after SNAP treatment. C-D, Quantification of spectral power differences in AuC (C) and FC (D) of Ctrl WT and cKO mice before and after SNAP treatment. Mice were first recorded at P27-P29 and then one or two days later following SNAP treatment. The enhanced high frequency gamma oscillations can be visually observed in FC of cKO mice following SNAP treatment. Mixed-effects analysis controlling for the effect of movement revealed differences in the gamma range in FC of cKO SNAP mice with Bonferroni's multiple comparisons test (*p<0.05, **p<0.01). The gamma band was further subdivided into low and high gamma revealing treatment differences in high gamma bands. E-H, Power coupling of different oscillation frequencies. Graphs show Pearson’s correlation (r) for Alpha frequency power coupling (E,G): AuC Alpha/FC Alpha, AuC Alpha/AuC Gamma, AuC Alpha/FC Gamma, FC Alpha/AuC Gamma, FC Alpha/FC Gamma; and Delta frequency power coupling (F,H): AuC Delta/FC Delta, AuC Delta/AuC Gamma, AuC Delta/FC Gamma, FC Delta/AuC Gamma, FC Delta/FC Gamma (*p<0.05; **p<0.01; two-way ANOVA, Bonferroni's multiple comparisons test). Vehicle, but not SNAP treatment, had negative effects in the WT group; both vehicle and SNAP did not affect A/G and D/G coupling in KO groups.


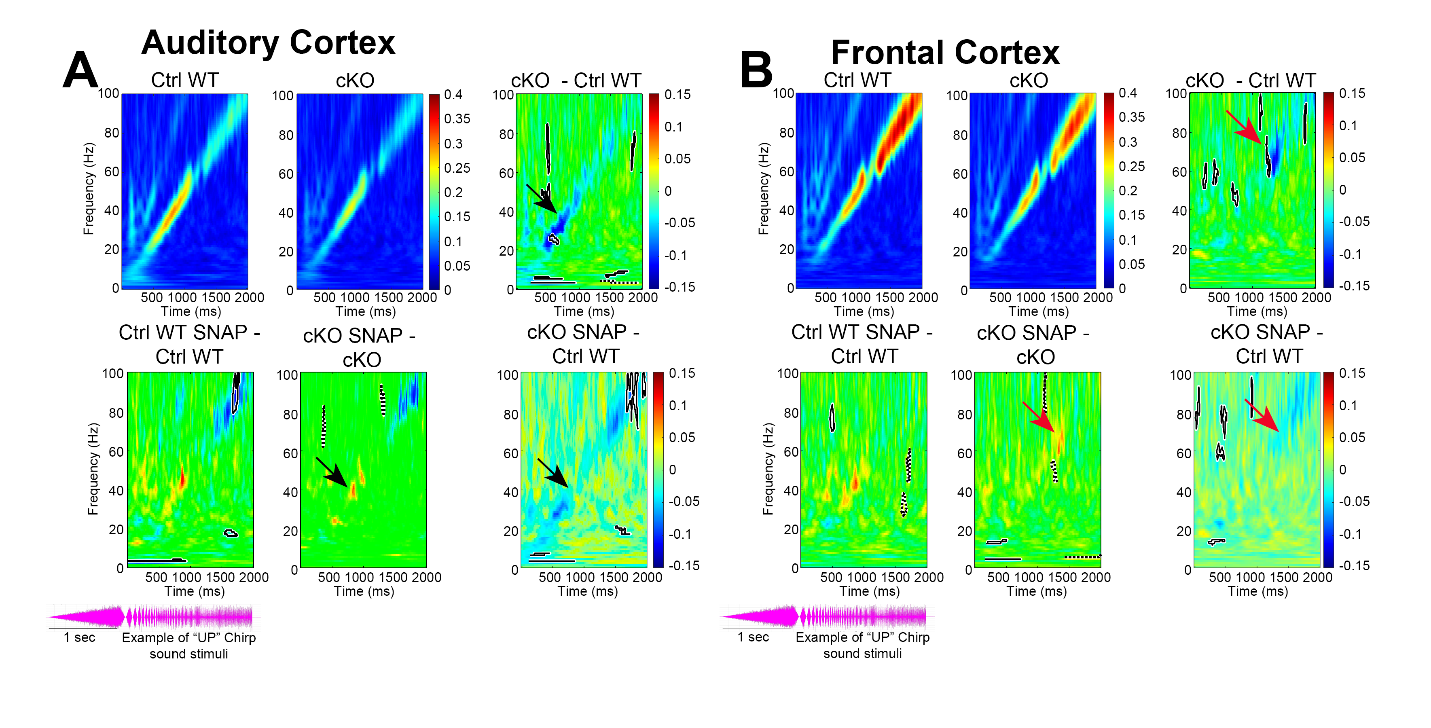


**Figure S5 Acute blockade of GAT3-mediated astrocytic GABA transport improves fidelity of temporal processing to the frequency-modulated sound chirp in high gamma range in both cKO and global Fmr1 KO mice**

The chirp stimulus (oscillogram shown at the bottom of this figure) is a 2 s broadband noise with amplitude modulated linearly by a frequency sweep with frequencies increasing from 1 to 100 Hz. To prevent the early response to stimulus onset, the chirp is preceded by a 1 s slow ramp of broadband noise. The ability of the cortical neural generators to follow this temporally dynamic stimulus is quantified by measuring the inter-trial phase coherence (ITPC, also known as phase locking factor). Trains of chirp stimuli were presented to each mouse 300 times. For each mouse, ITPC was measured to determine the degree of phase locking across trials. A-B, Grand average matrices were calculated for each genotype and treatment, and then Ctrl WT (n=12) average values were subtracted from cKO (n=13) average values (right panels) and pre-treatment values were subtracted from SNAP-treated values (bottom panels) for AuC (A) and FC (B). SNAP-treated cKO was also compared to Ctrl WT group. Blue areas indicate less phase locking, green areas no difference, and red areas more phase locking. Statistical cluster analysis reveals contiguous time x frequency regions that are significantly different between genotypes and treatments. Black solid contours (mean negative difference) and black dashed contours (mean positive difference) indicate clusters with significant differences. cKO mice express statistically significant decrease in ITPC at gamma frequencies (30-100 Hz, blue) that was restored to Ctrl WT levels following SNAP treatment.


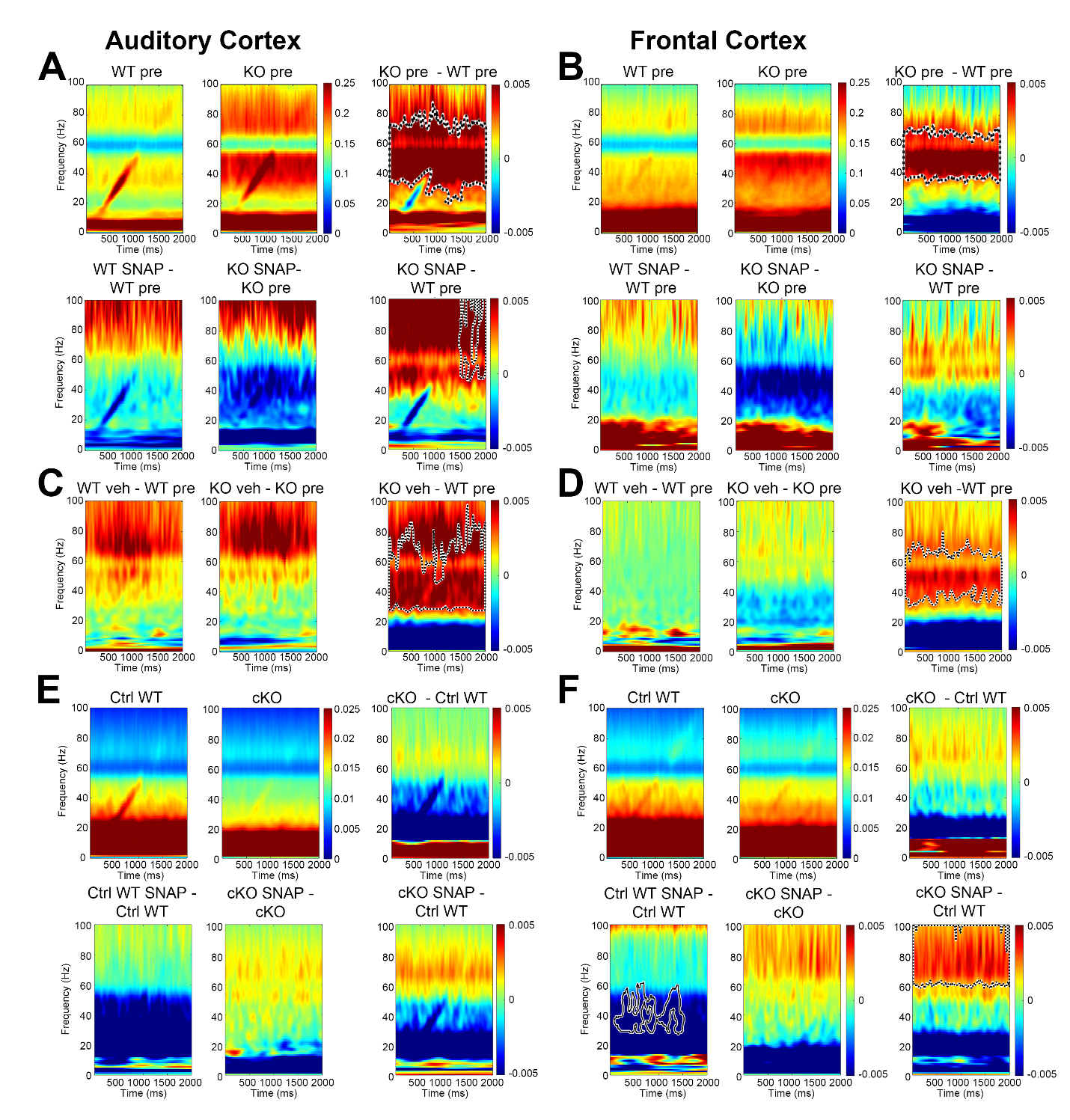


**Figure S6: Acute blockage of GAT3-mediated GABA transport in astrocytes normalized background gamma power in the auditory and frontal cortex of global KO mice during chirp presentation, related to Figure 7**

For each mouse, single-trial power (STP) was measured to determine the average total non-phase locked power during chirp train presentation. A-B, Grand average of STP values were calculated for global KO (n=13) and WT (n=21) before (pre) and global KO (n=7) and WT (n=11) after SNAP treatment and subtracted to generate difference plots by genotype (right panels) and treatment (bottom panels) for AuC (A) and FC (B). Global KO mice express statistically significant increase in STP at gamma frequencies (30-100 Hz, blue), with gamma STP restored to Ctrl WT levels in AuC and FC following SNAP treatment. C-D, Grand average matrices of STP values were calculated for global KO (n=20) and WT (n=22) before (pre) and global KO (n=12) and WT (n=16) after vehicle treatment and subtracted to generate difference plots by genotype (right panels) and treatment (bottom panels) for AuC (C) and FC (D). Vehicle treatment did not significantly affect STP values in gamma range. E-F, Grand average matrices were calculated for each genotype, and then Ctrl WT (n=12) STP values were subtracted from cKO (n=13) values and pre-treatment STP values were subtracted from SNAP-treated values (bottom panels) for AuC (E) and FC (F). Statistical cluster analysis reveals contiguous time x frequency regions that are significantly different between genotypes. Black dashed contour indicates these significant clusters. Consistent with the increase in gamma power changes in baseline EEGs, SNAP-treated cKO mice express statistically significant increase in non-phase gamma power range (30-100 Hz, red) throughout sound presentation in FC.


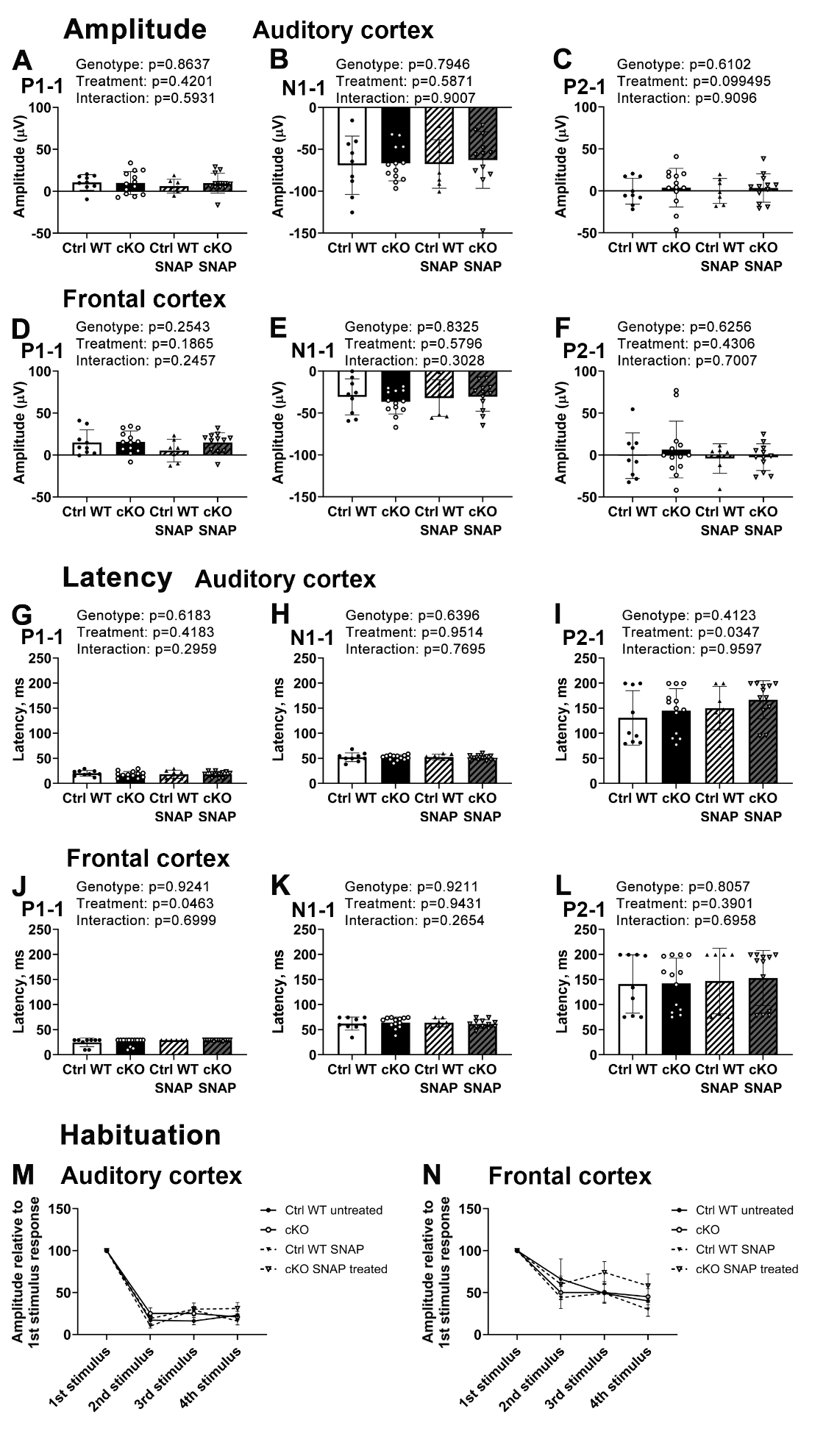


**Figure S7: Astrocyte-specific Fmr1 deletion does not affect habituation, latency or amplitude of ERP waves, related to Figure 7**

A-L, Auditory ERP amplitudes (A-F) and latencies (G-L) in AuC and FC of Ctrl WT (n=9), cKO (n=13), Ctrl WT SNAP (n=7), cKO SNAP (n=12) mice. Grand average ERPs obtained from P28 mice in response to the first 100-ms broadband noise presented at 4Hz repetition rate. P1, N1, and P2 were defined as maximum or minimum voltage deflections within 0–30 ms, 30–80 ms, or 80–150 ms, respectively. A-L, Graphs show mean ± SD of P1 amplitude in the AuC (A) and FC (D); N1 amplitude in the AuC (B) and FC (E); P2 amplitude in the AuC (C) and FC (F); P1 latency in the AuC (G) and FC (J); N1 latency in the AuC (H) and FC (K); P2 latency in the AuC (I) and FC (L) (p=0.05; Mixed-effects analysis, Bonferroni's multiple comparisons test). No significant differences were observed with post-hoc testing; however, there is an effect of treatment on latency in P1 in FC and P2 in AuC. M-N, Habituation is indicated by N1 amplitude of subsequent stimuli relative to first stimulus amplitude in 4Hz sound train in AuC (M) and FC (N). Graphs show mean ± SD. No differences are observed in habituation.

# Table S1

Statistics for Figure 1B

| % GFAP/S100β+ cells | **Mean** | **SD** | **N** |
| --- | --- | --- | --- |
| CON | 96.58 | 0.5563 | 3 |
| FXS | 97.79 | 0.8383 | 3 |
| Statistics | Two-tailed, unpaired t-test | t=2.089, df=4, p=0.1050  η²= 0.5217 |  |

List of primer sequences used for qRT-PCR in Figures 1C, 1E-1G, S1C, S2A-S2D

| **Gene** | **Forward primer sequence**  **(5′–3′)** | **Reverse primer sequence**  **(5′–3′)** |
| --- | --- | --- |
| **Human** |  |  |
| *Fmr1* | GGG GAA TCC CAG AAA CCT GAA | CGC AAC TGG TCT ACT TCC TTT A |
| *GFAP* | ACC TGC AGA TTC GAG AAA CCA G | GGT CCT GCC TCA CAT CAC ATC |
| *S100B* | GTG GCC CTC ATC GAC GTT TT | ACC TCC TGC TCT TTG ATT TCC TCT |
| *Slc1a3* | ATT CCA GCA GGG AGT CCG TA | TCC AAG GAT TGT ACC CAC AAT GA |
| *GAPDH* | TTG GCT ACA GCA ACA GGG TG | GGG GAG ATT CAG TGT GGT GG |
| **Mouse** |  |  |
| *Slc6a11* | CGG CTG GGT ATA TGG AAG CA | ACG ACT TTC CAG CAC CAC TT |
| *Gabra1* | CAGAAAAGCCAAAGAAAGTAAAGGA | TGGTTGCTGTAGGAGCATATGTG |
| *Gabra3* | GCTGCTCAGACTGGTAGATAATGG | GGGCATTCAGCGTGTATTGTT |
| *Gabrg2* | CCTGCCCCCTGGAGTTCT | ACTGCGCTTCCATTGATAAACA |
| *Gabra5* | GCAGACAGTAGGCACTGAGAACA | GGAAGTGAGCAGTCATGATCGTAT |
| *GAPDH* | ACTCCACTCACGGCAAATTC | TCTCCATGGTGGTGAAGACA |

Statistics for Figure 1C

| FMR1 mRNA | **Mean** | **SD** | **N** |
| --- | --- | --- | --- |
| CON | 0.9638 | 0.6170 | 5 |
| FXS | 0.02298 | 0.04554 | 5 |
| Statistics | Two-tailed, unpaired t-test | t=3.400, df=8, p=0.0094  η²= 0.5910 |  |

Statistics for Figure 1D

| FMRP protein | **Mean** | **SD** | **N** |
| --- | --- | --- | --- |
| CON | 1.000 | 0.8166 | 3 |
| FXS | 0.05250 | 0.02192 | 2 |
| Statistics | Two-tailed, unpaired t-test | t=1.557, df=3, p=0.2173  η²= 0.4469 |  |

Statistics for Figure 1E

| GFAP mRNA | **Mean** | **SD** | **N** |
| --- | --- | --- | --- |
| CON | 0.8353 | 0.4516 | 6 |
| FXS | 0.4433 | 0.5092 | 5 |
| Statistics | Two-tailed, unpaired t-test | t=1.354, df=9, p=0.2088  η²= 0.1692 |  |

Statistics for Figure 1F

| S100β mRNA | **Mean** | **SD** | **N** |
| --- | --- | --- | --- |
| CON | 0.9639 | 0.3202 | 6 |
| FXS | 0.5080 | 0.4427 | 5 |
| Statistics | Two-tailed, unpaired t-test | t=1.984, df=9, p=0.0786  η²= 0.3043 |  |

Statistics for Figure 1G

| SLC1A3 mRNA | **Mean** | **SD** | **N** |
| --- | --- | --- | --- |
| CON | 0.7899 | 0.5538 | 6 |
| FXS | 0.2821 | 0.2340 | 5 |
| Statistics | Two-tailed, unpaired t-test | t=1.900, df=9, p=0.0899  η²= 0.2863 |  |

Statistics for Figure 1H

| GABA HPLC | **Mean** | **SD** | **N** |
| --- | --- | --- | --- |
| CON | 1.277 | 1.425 | 3 |
| FXS | 8.768 | 3.469 | 4 |
| Statistics | Two-tailed, unpaired t-test | t=3.461, df=5, p=0.0180  η²= 0.7055 |  |

Statistics for Figure 1I

| GAD65/67 protein | **Mean** | **SD** | **N** |
| --- | --- | --- | --- |
| CON | 1.000 | 0.1376 | 6 |
| FXS | 1.273 | 0.2046 | 6 |
| Statistics | Two-tailed, unpaired t-test | t=2.712, df=10, p=0.0219 η²= 0.4237 |  |

# Table S2

Statistics for Figure 2A

| GABA levels | **Mean** | **SD** | **N** |
| --- | --- | --- | --- |
| WT | 1.000 | 0.09537 | 5 |
| KO | 1.248 | 0.1684 | 5 |
| Statistics | Two-tailed, unpaired t-test | t=2.864, df=8, p=0.0210  η²=0.5062 |  |

Statistics for Figure 2B

| GAD65/67 protein | **Mean** | **SD** | **N** |
| --- | --- | --- | --- |
| WT | 1.000 | 0.03853 | 5 |
| KO | 1.525 | 0.4230 | 6 |
| Statistics | Two-tailed, unpaired t-test | t=2.742, df=9, p=0.0228  η²=0.4551 |  |

| Aldha1 protein | **Mean** | **SD** | **N** |
| --- | --- | --- | --- |
| WT | 1.000 | 0.3121 | 5 |
| KO | 0.8600 | 0.1255 | 6 |
| Statistics | Two-tailed, unpaired t-test | t=1.013, df=9, p=0.3373  η²=0.1024 |  |

# Table S3

Statistics for Figure 3C

| FMRP in astrocytes | **Mean** | **SD** | **N** |
| --- | --- | --- | --- |
| Ctrl WT | 14.3 | 1.66 | 3 |
| cKO | 1.76 | 0.19 | 3 |
| Statistics | Two-tailed, unpaired t-test | t=12.93, df=4, p=0.0002  η²=0.9768 |  |

Statistics for Figure 3E

GABA levels in astrocytes in FC

| **ANOVA table** | **SS (Type III)** | **DF** | **MS** | **F (DFn, DFd)** | **P value** | **% of total variation** |
| --- | --- | --- | --- | --- | --- | --- |
| Genotype x Treatment | 3309 | 1 | 3309 | F (1, 85) = 6.717 | P=0.0112 | 4.540 |
| Genotype | 27351 | 1 | 27351 | F (1, 85) = 55.52 | P<0.0001 | 37.53 |
| Treatment | 1844 | 1 | 1844 | F (1, 85) = 3.743 | P=0.0563 | 2.530 |

| **Šídák's multiple comparisons test** | **Predicted (LS) mean diff.** | **95.00% CI of**  **diff.** | **Individual**  **P Value** |
| --- | --- | --- | --- |
| Ctrl WT |  |  |  |
| Vehicle vs. SNAP | 3.106 | -11.48 to 17.69 | 0.8624 |
| cKO |  |  |  |
| Vehicle vs. SNAP | -21.40 | -37.22 to -5.571 | 0.0056 |
| Vehicle |  |  |  |
| Ctrl WT vs. cKO | -22.97 | -37.88 to -8.055 | 0.0015 |
| SNAP |  |  |  |
| Ctrl WT vs. cKO | -47.47 | -62.99 to -31.95 | <0.0001 |

Statistics for Figure 3F

GABA levels in astrocytes in AuC

| **ANOVA table** | **SS (Type III)** | **DF** | **MS** | **F (DFn, DFd)** | **P value** | **% of total variation** |
| --- | --- | --- | --- | --- | --- | --- |
| Genotype x Treatment | 11279 | 1 | 11279 | F (1, 117) = 21.84 | P<0.0001 | 10.75 |
| Genotype | 16385 | 1 | 16385 | F (1, 117) = 31.73 | P<0.0001 | 15.61 |
| Treatment | 18867 | 1 | 18867 | F (1, 117) = 36.53 | P<0.0001 | 17.98 |

| **Šídák's multiple comparisons test** | **Predicted (LS) mean diff.** | **95.00% CI of**  **diff.** | **Individual**  **P Value** |
| --- | --- | --- | --- |
| Ctrl WT |  |  |  |
| Vehicle vs. SNAP | -5.838 | -17.85 to 6.171 | 0.4715 |
| cKO |  |  |  |
| Vehicle vs. SNAP | -45.65 | -60.75 to -30.54 | <0.0001 |
| Vehicle |  |  |  |
| Ctrl WT vs. cKO | -4.086 | -18.37 to 10.20 | 0.7681 |
| SNAP |  |  |  |
| Ctrl WT vs. cKO | -43.89 | -56.86 to -30.92 | <0.0001 |

Statistics for Figure 3G

| GAT3 protein AC | **Mean** | **SD** | **N** |
| --- | --- | --- | --- |
| Ctrl WT | 1.000 | 0.3487 | 5 |
| cKO | 0.9848 | 0.2905 | 5 |
| Statistics | Two-tailed, unpaired t-test | t=0.07491, df=8, p=0.9421  η²=0.0007010 |  |

| GAT3 protein FC | **Mean** | **SD** | **N** |
| --- | --- | --- | --- |
| Ctrl WT | 1.000 | 0.08737 | 5 |
| cKO | 1.052 | 0.2372 | 4 |
| Statistics | Two-tailed, unpaired t-test | t=0.4597, df=7, p=0.6597  η²=0.02930 |  |

Statistics for Figure S1B

| GAT3 mRNA in AuC | **Mean** | **SD** | **N** |
| --- | --- | --- | --- |
| Ctrl WT | 1.014 | 0.1869 | 5 |
| cKO | 0.9697 | 0.09106 | 4 |
| Statistics | Two-tailed, unpaired t-test | t=0.4309, df=7, p=0.6795  η²=0.02584 |  |

| GAT3 mRNA in FC | **Mean** | **SD** | **N** |
| --- | --- | --- | --- |
| Ctrl WT | 1.013 | 0.1774 | 5 |
| cKO | 0.9782 | 0.05646 | 4 |
| Statistics | Two-tailed, unpaired t-test | t=0.3762, df=7, p=0.7179  η²=0.01982 |  |

# Table S4

Statistics for Figure 4C

vGAT/Gephyrin co-localization

| **ANOVA table** | **SS (Type III)** | **DF** | **MS** | **F (DFn, DFd)** | **P value** | **% of total variation** |
| --- | --- | --- | --- | --- | --- | --- |
| Brain region x Genotype | 9.698 | 1 | 9.698 | F (1, 129) = 0.02451 | P=0.8758 | 0.005079 |
| Brain region | 134845 | 1 | 134845 | F (1, 129) = 340.8 | P<0.0001 | 70.62 |
| Genotype | 7143 | 1 | 7143 | F (1, 129) = 18.05 | P<0.0001 | 3.741 |

| **Šídák's multiple comparisons test** | **Predicted (LS) mean diff.** | **95.00% CI of**  **diff.** | **Individual**  **P Value** |
| --- | --- | --- | --- |
| AuC |  |  |  |
| Ctrl WT vs. cKO | 15.21 | 4.291 to 26.13 | 0.0040 |
| FC |  |  |  |
| Ctrl WT vs. cKO | 14.13 | 2.954 to 25.31 | 0.0098 |
| Ctrl WT |  |  |  |
| AC vs. FC | -63.21 | -74.31 to -52.11 | <0.0001 |
| cKO |  |  |  |
| AC vs. FC | -64.29 | -75.29 to -53.29 | <0.0001 |

Statistics for Figure 4D

vGAT puncta density

| **ANOVA table** | **SS (Type III)** | **DF** | **MS** | **F (DFn, DFd)** | **P value** | **% of total variation** |
| --- | --- | --- | --- | --- | --- | --- |
| Brain region x Genotype | 28.84 | 1 | 28.84 | F (1, 129) = 0.04784 | P=0.8272 | 0.009404 |
| Brain region | 224452 | 1 | 224452 | F (1, 129) = 372.3 | P<0.0001 | 73.19 |
| Genotype | 8242 | 1 | 8242 | F (1, 129) = 13.67 | P=0.0003 | 2.688 |

| **Šídák's multiple comparisons test** | **Predicted (LS) mean diff.** | **95.00% CI of**  **diff.** | **Individual**  **P Value** |
| --- | --- | --- | --- |
| AuC |  |  |  |
| Ctrl WT vs. cKO | 14.83 | 1.132 to 28.54 | 0.0310 |
| FC |  |  |  |
| Ctrl WT vs. cKO | 16.70 | 3.111 to 30.29 | 0.0124 |
| Ctrl WT |  |  |  |
| AC vs. FC | -83.21 | -96.80 to -69.62 | <0.0001 |
| cKO |  |  |  |
| AC vs. FC | -81.35 | -95.05 to -67.65 | <0.0001 |

Statistics for Figure 4E

PV/vGAT co-localization

| **ANOVA table** | **SS (Type III)** | **DF** | **MS** | **F (DFn, DFd)** | **P value** | **% of total variation** |
| --- | --- | --- | --- | --- | --- | --- |
| Brain region x Genotype | 133.9 | 1 | 133.9 | F (1, 64) = 1.319 | P=0.2550 | 0.7827 |
| Brain region | 10064 | 1 | 10064 | F (1, 64) = 99.14 | P<0.0001 | 58.83 |
| Genotype | 452.7 | 1 | 452.7 | F (1, 64) = 4.459 | P=0.0386 | 2.646 |

| **Šídák's multiple comparisons test** | **Predicted (LS) mean diff.** | **95.00% CI of**  **diff.** | **Individual**  **P Value** |
| --- | --- | --- | --- |
| AuC |  |  |  |
| Ctrl WT vs. cKO | 2.356 | -5.572 to 10.28 | 0.7487 |
| FC |  |  |  |
| Ctrl WT vs. cKO | 7.974 | 0.05942 to 15.89 | 0.0480 |
| Ctrl WT |  |  |  |
| AC vs. FC | -27.16 | -34.96 to -19.36 | <0.0001 |
| cKO |  |  |  |
| AC vs. FC | -21.54 | -29.58 to -13.51 | <0.0001 |

Statistics for Figure 4F

PV puncta density

| **ANOVA table** | **SS (Type III)** | **DF** | **MS** | **F (DFn, DFd)** | **P value** | **% of total variation** |
| --- | --- | --- | --- | --- | --- | --- |
| Brain region x Genotype | 259.3 | 1 | 259.3 | F (1, 63) = 2.469 | P=0.1211 | 1.504 |
| Brain region | 9745 | 1 | 9745 | F (1, 63) = 92.81 | P<0.0001 | 56.51 |
| Genotype | 537.8 | 1 | 537.8 | F (1, 63) = 5.122 | P=0.0271 | 3.119 |

| **Šídák's multiple comparisons test** | **Predicted (LS) mean diff.** | **95.00% CI of**  **diff.** | **Individual**  **P Value** |
| --- | --- | --- | --- |
| AuC |  |  |  |
| Ctrl WT vs. cKO | 1.734 | -6.332 to 9.800 | 0.8587 |
| FC |  |  |  |
| Ctrl WT vs. cKO | 9.612 | 1.436 to 17.79 | 0.0180 |
| Ctrl WT |  |  |  |
| AC vs. FC | -28.09 | -36.03 to -20.15 | <0.0001 |
| cKO |  |  |  |
| AC vs. FC | -20.21 | -28.51 to -11.91 | <0.0001 |

Statistics for Figure S2A

| Gabrg2 mRNA in AuC | **Mean** | **SD** | **N** |
| --- | --- | --- | --- |
| Ctrl WT | 1.110 | 0.09707 | 5 |
| cKO | 0.8950 | 0.1137 | 4 |
| Statistics | Two-tailed, unpaired t-test | t=3.062, df=7, p= 0.0183  η²= 0.5726 |  |

| Gabrg2 mRNA in FC | **Mean** | **SD** | **N** |
| --- | --- | --- | --- |
| Ctrl WT | 1.034 | 0.03454 | 4 |
| cKO | 1.225 | 0.2826 | 4 |
| Statistics | Two-tailed, unpaired t-test | t=1.344, df=6, p= 0.2275  η²= 0.2315 |  |

Statistics for Figure S2B

| Gabra1 mRNA in AuC | **Mean** | **SD** | **N** |
| --- | --- | --- | --- |
| Ctrl WT | 1.009 | 0.1513 | 5 |
| cKO | 0.7900 | 0.1137 | 4 |
| Statistics | Two-tailed, unpaired t-test | t=2.389, df=7, p= 0.0483  η²= 0.4491 |  |

| Gabra1 mRNA in FC | **Mean** | **SD** | **N** |
| --- | --- | --- | --- |
| Ctrl WT | 1.009 | 0.1549 | 5 |
| cKO | 1.128 | 0.2892 | 4 |
| Statistics | Two-tailed, unpaired t-test | t=0.7962, df=7, p= 0.4521  η²= 0.08305 |  |

Statistics for Figure S2C

| Gabra3 mRNA in AuC | **Mean** | **SD** | **N** |
| --- | --- | --- | --- |
| Ctrl WT | 1.005 | 0.1157 | 5 |
| cKO | 1.020 | 0.2069 | 4 |
| Statistics | Two-tailed, unpaired t-test | t=0.1403, df=7, p=0.8923  η²=0.002806 |  |

| Gabra3 mRNA in FC | **Mean** | **SD** | **N** |
| --- | --- | --- | --- |
| Ctrl WT | 1.006 | 0.1299 | 5 |
| cKO | 0.9004 | 0.1800 | 4 |
| Statistics | Two-tailed, unpaired t-test | t=1.030, df=7, p= 0.3371  η²= 0.1317 |  |

Statistics for Figure S2D

| Gabra5 mRNA in AuC | **Mean** | **SD** | **N** |
| --- | --- | --- | --- |
| Ctrl WT | 1.024 | 0.2327 | 5 |
| cKO | 1.262 | 0.3424 | 4 |
| Statistics | Two-tailed, unpaired t-test | t=1.248, df=7, p= 0.2523  η²= 0.1819 |  |

| Gabra5 mRNA in FC | **Mean** | **SD** | **N** |
| --- | --- | --- | --- |
| Ctrl WT | 1.004 | 0.1087 | 5 |
| cKO | 0.9706 | 0.2355 | 4 |
| Statistics | Two-tailed, unpaired t-test | t=0.2885, df=7, p= 0.7813  η²= 0.01175 |  |

Statistics for Figure S2E

| GABAARγ2 protein in AuC | **Mean** | **SD** | **N** |
| --- | --- | --- | --- |
| Ctrl WT | 1.039 | 0.09588 | 4 |
| cKO | 1.073 | 0.1546 | 5 |
| Statistics | Two-tailed, unpaired t-test | t=0.3914, df=7, p= 0.7071  η²= 0.02142 |  |

| GABAARγ2 protein in FC | **Mean** | **SD** | **N** |
| --- | --- | --- | --- |
| Ctrl WT | 0.9726 | 0.06572 | 5 |
| cKO | 0.9810 | 0.2137 | 4 |
| Statistics | Two-tailed, unpaired t-test | t=0.07529, df=7, p= 0.9421  η²= 0.0008092 |  |

Statistics for Figure S2F

| GABAARα5 protein in AuC | **Mean** | **SD** | **N** |
| --- | --- | --- | --- |
| Ctrl WT | 0.9998 | 0.1930 | 4 |
| cKO | 1.226 | 0.2028 | 4 |
| Statistics | Two-tailed, unpaired t-test | t=1.616, df=6, p= 0.1571  η²= 0.3033 |  |

| GABAARα5 protein in FC | **Mean** | **SD** | **N** |
| --- | --- | --- | --- |
| Ctrl WT | 0.9245 | 0.2970 | 4 |
| cKO | 0.9165 | 0.4539 | 4 |
| Statistics | Two-tailed, unpaired t-test | t=0.02950, df=6, p= 0.9774  η²= 0.0001450 |  |

# Table S5

Statistics for Figure 5C

PV levels

| **ANOVA table** | **SS (Type III)** | **DF** | **MS** | **F (DFn, DFd)** | **P value** | **% of total variation** |
| --- | --- | --- | --- | --- | --- | --- |
| Genotype x Treatment | 9575 | 1 | 9575 | F (1, 52) = 5.794 | P=0.0197 | 7.451 |
| Genotype | 5878 | 1 | 5878 | F (1, 52) = 3.557 | P=0.0649 | 4.574 |
| Treatment | 31319 | 1 | 31319 | F (1, 52) = 18.95 | P<0.0001 | 24.37 |

| **Tukey's multiple comparisons test** | **Predicted (LS) mean diff.** | **95.00% CI of**  **diff.** | **Adjusted P Value** |
| --- | --- | --- | --- |
| Ctrl WT :Vehicle vs. Ctrl WT :SNAP | -21.36 | -59.51 to 16.78 | 0.4528 |
| Ctrl WT :Vehicle vs. cKO:Vehicle | 47.13 | 5.924 to 88.33 | 0.0190 |
| Ctrl WT :Vehicle vs. cKO:SNAP | -27.09 | -68.29 to 14.12 | 0.3115 |
| Ctrl WT :SNAP vs. cKO:Vehicle | 68.49 | 27.29 to 109.7 | 0.0003 |
| Ctrl WT :SNAP vs. cKO:SNAP | -5.721 | -46.92 to 35.48 | 0.9827 |
| cKO:Vehicle vs. cKO:SNAP | -74.21 | -118.3 to -30.16 | 0.0002 |

Statistics for Figure 5D

PV cell density

| **ANOVA table** | **SS (Type III)** | **DF** | **MS** | **F (DFn, DFd)** | **P value** | **% of total variation** |
| --- | --- | --- | --- | --- | --- | --- |
| Genotype x Treatment | 0.1490 | 1 | 0.1490 | F (1, 52) = 0.7172 | P=0.4009 | 1.218 |
| Genotype | 0.9763 | 1 | 0.9763 | F (1, 52) = 4.700 | P=0.0348 | 7.983 |
| Treatment | 0.2392 | 1 | 0.2392 | F (1, 52) = 1.152 | P=0.2882 | 1.956 |

| **Tukey's multiple comparisons test** | **Predicted (LS) mean diff.** | **95.00% CI of**  **diff.** | **Adjusted P Value** |
| --- | --- | --- | --- |
| Ctrl WT :Vehicle vs. Ctrl WT :SNAP | -0.2363 | -0.6640 to 0.1914 | 0.4648 |
| Ctrl WT :Vehicle vs. cKO:Vehicle | -0.3710 | -0.8330 to 0.09091 | 0.1566 |
| Ctrl WT :Vehicle vs. cKO:SNAP | -0.3989 | -0.8608 to 0.06307 | 0.1131 |
| Ctrl WT :SNAP vs. cKO:Vehicle | -0.1347 | -0.5967 to 0.3272 | 0.8658 |
| Ctrl WT :SNAP vs. cKO:SNAP | -0.1626 | -0.6245 to 0.2994 | 0.7867 |
| cKO:Vehicle vs. cKO:SNAP | -0.02784 | -0.5217 to 0.4660 | 0.9988 |

Statistics for Figure 5E

cFos cell density

| **ANOVA table** | **SS (Type III)** | **DF** | **MS** | **F (DFn, DFd)** | **P value** | **% of total variation** |
| --- | --- | --- | --- | --- | --- | --- |
| Genotype x Treatment | 89.11 | 1 | 89.11 | F (1, 51) = 8.039 | P=0.0065 | 12.83 |
| Genotype | 33.83 | 1 | 33.83 | F (1, 51) = 3.052 | P=0.0866 | 4.870 |
| Treatment | 0.2051 | 1 | 0.2051 | F (1, 51) = 0.01850 | P=0.8923 | 0.02952 |

| **Tukey's multiple comparisons test** | **Predicted (LS) mean diff.** | **95.00% CI of**  **diff.** | **Adjusted P Value** |
| --- | --- | --- | --- |
| Ctrl WT :Vehicle vs. Ctrl WT :SNAP | -2.706 | "-5.832 to 0.4204" | 0.1118 |
| Ctrl WT :Vehicle vs. cKO:Vehicle | -4.173 | "-7.549 to -0.7963" | 0.0098 |
| Ctrl WT :Vehicle vs. cKO:SNAP | -1.715 | "-5.178 to 1.748" | 0.5578 |
| Ctrl WT :SNAP vs. cKO:Vehicle | -1.467 | "-4.844 to 1.909" | 0.6582 |
| Ctrl WT :SNAP vs. cKO:SNAP | 0.9909 | "-2.472 to 4.454" | 0.8720 |
| cKO:Vehicle vs. cKO:SNAP | 2.458 | "-1.233 to 6.149" | 0.3001 |

Statistics for Figure S3A

PV levels in L1-4

| **ANOVA table** | **SS (Type III)** | **DF** | **MS** | **F (DFn, DFd)** | **P value** | **% of total variation** |
| --- | --- | --- | --- | --- | --- | --- |
| Genotype x Treatment | 4446 | 1 | 4446 | F (1, 52) = 6.160 | P=0.0163 | 8.417 |
| Genotype | 2317 | 1 | 2317 | F (1, 52) = 3.211 | P=0.0790 | 4.388 |
| Treatment | 10180 | 1 | 10180 | F (1, 52) = 14.11 | P=0.0004 | 19.28 |

| **Bonferroni's multiple comparisons test** | **Predicted (LS) mean diff.** | **95.00% CI of**  **diff.** | **Individual**  **P Value** |
| --- | --- | --- | --- |
| Ctrl WT |  |  |  |
| Vehicle vs. SNAP | -9.241 | -31.16 to 12.68 | 0.6702 |
| cKO |  |  |  |
| Vehicle vs. SNAP | -45.25 | -70.57 to -19.93 | 0.0003 |
| Vehicle |  |  |  |
| Ctrl WT vs. cKO | 31.00 | 7.323 to 54.68 | 0.0078 |
| SNAP |  |  |  |
| Ctrl WT vs. cKO | -5.006 | -28.69 to 18.67 | >0.9999 |

PV levels in L5-6

| **ANOVA table** | **SS (Type III)** | **DF** | **MS** | **F (DFn, DFd)** | **P value** | **% of total variation** |
| --- | --- | --- | --- | --- | --- | --- |
| Genotype x Treatment | 2386 | 1 | 2386 | F (1, 52) = 1.988 | P=0.1645 | 2.808 |
| Genotype | 1649 | 1 | 1649 | F (1, 52) = 1.375 | P=0.2464 | 1.942 |
| Treatment | 20073 | 1 | 20073 | F (1, 52) = 16.73 | P=0.0002 | 23.63 |

| **Bonferroni's multiple comparisons test** | **Predicted (LS) mean diff.** | **95.00% CI of**  **diff.** | **Individual**  **P Value** |
| --- | --- | --- | --- |
| Ctrl WT |  |  |  |
| Vehicle vs. SNAP | -25.07 | -53.34 to 3.198 | 0.0914 |
| cKO |  |  |  |
| Vehicle vs. SNAP | -51.45 | -84.09 to -18.81 | 0.0013 |
| Vehicle |  |  |  |
| Ctrl WT vs. cKO | 24.16 | -6.376 to 54.69 | 0.1471 |
| SNAP |  |  |  |
| Ctrl WT vs. cKO | -2.222 | -32.75 to 28.31 | >0.9999 |

Statistics for Figure S3B

Proportion of low PV and high PV cells

| **ANOVA table** | **SS (Type III)** | **DF** | **MS** | **F (DFn, DFd)** | **P value** | **% of total variation** |
| --- | --- | --- | --- | --- | --- | --- |
| PV expression x Genotype/  Treatment | 40150 | 3 | 13383 | F (3, 104) = 18.74 | P<0.0001 | 16.64 |
| PV expression | 116360 | 1 | 116360 | F (1, 104) = 162.9 | P<0.0001 | 48.22 |
| Genotype/  Treatment | 2.972e-027 | 3 | 9.905e-028 | F (3, 104) = 1.387e-030 | P>0.9999 | 1.231e-030 |

| **Tukey's multiple comparisons test** | **Predicted (LS) mean diff.** | **95.00% CI of**  **diff.** | **Adjusted P Value** |
| --- | --- | --- | --- |
| low PV |  |  |  |
| Ctrl WT vs. cKO | -30.93 | -57.58 to -4.280 | 0.0160 |
| Ctrl WT vs. Ctrl WT SNAP | 17.67 | -7.001 to 42.35 | 0.2473 |
| Ctrl WT vs. cKO SNAP | 18.00 | -8.648 to 44.65 | 0.2967 |
| cKO vs. Ctrl WT SNAP | 48.60 | 21.95 to 75.25 | <0.0001 |
| cKO vs. cKO SNAP | 48.93 | 20.44 to 77.42 | 0.0001 |
| Ctrl WT SNAP vs. cKO SNAP | 0.3300 | -26.32 to 26.98 | >0.9999 |
| high PV |  |  |  |
| Ctrl WT vs. cKO | 30.93 | 4.280 to 57.58 | 0.0160 |
| Ctrl WT vs. Ctrl WT SNAP | -17.67 | -42.35 to 7.001 | 0.2473 |
| Ctrl WT vs. cKO SNAP | -18.00 | -44.65 to 8.648 | 0.2967 |
| cKO vs. Ctrl WT SNAP | -48.60 | -75.25 to -21.95 | <0.0001 |
| cKO vs. cKO SNAP | -48.93 | -77.42 to -20.44 | 0.0001 |
| Ctrl WT SNAP vs. cKO SNAP | -0.3300 | -26.98 to 26.32 | >0.9999 |
| Ctrl WT |  |  |  |
| low PV vs. high PV | -62.76 | -81.50 to -44.02 | <0.0001 |
| cKO |  |  |  |
| low PV vs. high PV | -0.9000 | -22.54 to 20.74 | 0.9344 |
| Ctrl WT SNAP |  |  |  |
| low PV vs. high PV | -98.11 | -116.8 to -79.37 | <0.0001 |
| cKO SNAP |  |  |  |
| low PV vs. high PV | -98.77 | -120.4 to -77.13 | <0.0001 |

Statistics for Figure S3C

Proportion of cFos positive PV cells

| **ANOVA table** | **SS** | **DF** | **MS** | **F (DFn, DFd)** | **P value** | **% of total**  **variation** |
| --- | --- | --- | --- | --- | --- | --- |
| PV level | 98384 | 1 | 98384 | F (1, 52) = 116.6 | P<0.0001 | 44.41 |
| Treatment | 9777 | 1 | 9777 | F (1, 52) = 12.20 | P=0.0010 | 4.413 |
| Genotype | 10233 | 1 | 10233 | F (1, 52) = 12.77 | P=0.0008 | 4.619 |
| PV level x Treatment | 6557 | 1 | 6557 | F (1, 52) = 7.773 | P=0.0074 | 2.960 |
| PV level x Genotype | 24.93 | 1 | 24.93 | F (1, 52) = 0.02956 | P=0.8642 | 0.01125 |
| Treatment x Genotype | 9016 | 1 | 9016 | F (1, 52) = 11.25 | P=0.0015 | 4.070 |
| PV level x Treatment x  Genotype | 2923 | 1 | 2923 | F (1, 52) = 3.465 | P=0.0683 | 1.319 |

| **Bonferroni's multiple comparisons test** | **Predicted (LS) mean diff.** | **95.00% CI of**  **diff.** | **Adjusted P Value** |
| --- | --- | --- | --- |
| low PV:Ctrl WT vs. high PV:Ctrl WT | -55.71 | "-91.44 to -13.94" | 0.0019 |
| low PV:cKO vs. high PV:cKO | -33.15 | "-45.56 to 43.94" | >0.9999 |
| low PV:Ctrl WT SNAP vs. high PV:Ctrl WT SNAP | -65.98 | "-114.4 to -36.91" | <0.0001 |
| low PV:cKO SNAP vs. high PV:cKO SNAP | -84.72 | "-131.0 to -41.47" | <0.0001 |
| low PV:Ctrl WT vs. low PV:cKO | -48.72 | "-69.30 to -5.534" | 0.0101 |
| low PV:Ctrl WT SNAP vs. low PV:cKO SNAP | 8.185 | "-31.70 to 32.06" | >0.9999 |
| high PV:Ctrl WT vs. high PV:cKO | -26.17 | "-17.42 to 46.35" | >0.9999 |
| high PV:Ctrl WT SNAP vs. high PV:cKO SNAP | -10.55 | "-42.26 to 21.51" | >0.9999 |
| low PV:Ctrl WT vs. low PV:Ctrl WT SNAP | 5.889 | "-18.55 to 40.48" | >0.9999 |
| low PV:cKO vs. low PV:cKO SNAP | 62.80 | "14.48 to 82.65" | 0.0007 |
| high PV:Ctrl WT vs. high PV:Ctrl WT SNAP | -4.389 | "-41.53 to 17.51" | >0.9999 |
| high PV:cKO vs. high PV:cKO SNAP | 11.23 | "-70.93 to -2.758" | 0.0243 |

# Table S6

Statistics for Figure 6C

Resting EEG AuC Delta

|  | **Mean** | **SD** | **N** |
| --- | --- | --- | --- |
| WT | 217.0 | 189.9 | 43 |
| KO | 208.5 | 152.8 | 32 |
| Statistics | Two-tailed, unpaired t-test | t=0.2100, df=73, p=0.8343  η²=0.0006036 |  |

Resting EEG AuC Theta

|  | **Mean** | **SD** | **N** |
| --- | --- | --- | --- |
| WT | 105.1 | 65.16 | 43 |
| KO | 99.98 | 63.66 | 33 |
| Statistics | Two-tailed, unpaired t-test | t=0.3460, df=74, p=0.7303  η²= 0.001615 |  |

Resting EEG AuC Alpha

|  | **Mean** | **SD** | **N** |
| --- | --- | --- | --- |
| WT | 38.71 | 28.33 | 43 |
| KO | 33.58 | 24.02 | 32 |
| Statistics | Two-tailed, unpaired t-test | t=0.8256, df=73, p= 0.4117  η²= 0.009250 |  |

Resting EEG AuC Low gamma

|  | **Mean** | **SD** | **N** |
| --- | --- | --- | --- |
| WT | 4.533 | 1.517 | 43 |
| KO | 6.018 | 2.181 | 33 |
| Statistics | Two-tailed, unpaired t-test | t=3.500, df=74, p= 0.0008  η²= 0.1420 |  |

Resting EEG AuC High gamma

|  | **Mean** | **SD** | **N** |
| --- | --- | --- | --- |
| WT | 2.102 | 1.281 | 42 |
| KO | 2.438 | 1.037 | 33 |
| Statistics | Two-tailed, unpaired t-test | t=1.226, df=73, p= 0.2243  η²= 0.02016 |  |

Statistics for Figure 6D

Resting EEG FC Delta

|  | **Mean** | **SD** | **N** |
| --- | --- | --- | --- |
| WT | 366.2 | 264.0 | 43 |
| KO | 286.3 | 184.6 | 32 |
| Statistics | Two-tailed, unpaired t-test | t=1.464, df=73, p= 0.1474  η²= 0.02853 |  |

Resting EEG FC Theta

|  | **Mean** | **SD** | **N** |
| --- | --- | --- | --- |
| WT | 183.7 | 103.6 | 43 |
| KO | 141.8 | 88.29 | 33 |
| Statistics | Two-tailed, unpaired t-test | t=1.863, df=74, p= 0.0665  η²= 0.04479 |  |

Resting EEG FC Alpha

|  | **Mean** | **SD** | **N** |
| --- | --- | --- | --- |
| WT | 57.26 | 31.79 | 42 |
| KO | 43.51 | 27.04 | 33 |
| Statistics | Two-tailed, unpaired t-test | t=1.983, df=73, p= 0.0511  η²= 0.05112 |  |

Resting EEG FC Low gamma

|  | **Mean** | **SD** | **N** |
| --- | --- | --- | --- |
| WT | 4.719 | 1.550 | 43 |
| KO | 5.800 | 1.919 | 33 |
| Statistics | Two-tailed, unpaired t-test | t=2.718, df=74, p= 0.0082  η²= 0.09074 |  |

Resting EEG FC High gamma

|  | **Mean** | **SD** | **N** |
| --- | --- | --- | --- |
| WT | 1.911 | 1.103 | 42 |
| KO | 2.074 | 0.8781 | 33 |
| Statistics | Two-tailed, unpaired t-test | t=0.6929, df=73, p=0.4905  η²= 0.006535 |  |

Statistics for Figure 6E

Resting EEG AuC Delta

| **ANOVA table** | **SS (Type III)** | **DF** | **MS** | **F (DFn, DFd)** | **P value** | **% of total variation** |
| --- | --- | --- | --- | --- | --- | --- |
| Genotype x Treatment | 81025 | 1 | 81025 | F (1, 45) = 1.476 | P=0.2307 | 2.407 |
| Genotype | 32309 | 1 | 32309 | F (1, 45) = 0.5886 | P=0.4470 | 0.9596 |
| Treatment | 611870 | 1 | 611870 | F (1, 45) = 11.15 | P=0.0017 | 18.17 |

| **Šídák's multiple comparisons test** | **Predicted (LS) mean diff.** | **95.00% CI of**  **diff.** | **Individual**  **P Value** |
| --- | --- | --- | --- |
| WT :Vehicle vs. WT :SNAP | 330.8 | 84.07 to 577.5 | 0.0036 |
| WT :Vehicle vs. KO:Vehicle | 144.0 | -85.74 to 373.7 | 0.4375 |
| WT :Vehicle vs. KO:SNAP | 298.3 | -5.640 to 602.2 | 0.0569 |
| WT :SNAP vs. KO:Vehicle | -186.8 | -446.6 to 72.95 | 0.2831 |
| WT :SNAP vs. KO:SNAP | -32.53 | -359.7 to 294.7 | >0.9999 |
| KO:Vehicle vs. KO:SNAP | 154.3 | -160.3 to 468.9 | 0.7046 |

Resting EEG AuC Theta

| **ANOVA table** | **SS (Type III)** | **DF** | **MS** | **F (DFn, DFd)** | **P value** | **% of total variation** |
| --- | --- | --- | --- | --- | --- | --- |
| Genotype x Treatment | 2946 | 1 | 2946 | F (1, 45) = 0.7603 | P=0.3879 | 1.478 |
| Genotype | 1036 | 1 | 1036 | F (1, 45) = 0.2674 | P=0.6076 | 0.5199 |
| Treatment | 15655 | 1 | 15655 | F (1, 45) = 4.041 | P=0.0504 | 7.856 |

| **Šídák's multiple comparisons test** | **Predicted (LS) mean diff.** | **95.00% CI of**  **diff.** | **Individual**  **P Value** |
| --- | --- | --- | --- |
| WT :Vehicle vs. WT :SNAP | 55.62 | -9.927 to 121.2 | 0.1360 |
| WT :Vehicle vs. KO:Vehicle | 26.81 | -34.23 to 87.85 | 0.7966 |
| WT :Vehicle vs. KO:SNAP | 48.78 | -31.97 to 129.5 | 0.4805 |
| WT :SNAP vs. KO:Vehicle | -28.82 | -97.83 to 40.20 | 0.8313 |
| WT :SNAP vs. KO:SNAP | -6.848 | -93.78 to 80.08 | >0.9999 |
| KO:Vehicle vs. KO:SNAP | 21.97 | -61.61 to 105.5 | 0.9786 |

Resting EEG AuC Alpha

| **ANOVA table** | **SS (Type III)** | **DF** | **MS** | **F (DFn, DFd)** | **P value** | **% of total variation** |
| --- | --- | --- | --- | --- | --- | --- |
| Genotype x Treatment | 75.15 | 1 | 75.15 | F (1, 45) = 0.1180 | P=0.7328 | 0.2364 |
| Genotype | 198.8 | 1 | 198.8 | F (1, 45) = 0.3123 | P=0.5791 | 0.6256 |
| Treatment | 2489 | 1 | 2489 | F (1, 45) = 3.909 | P=0.0542 | 7.831 |

Resting EEG AuC Low Gamma

| **ANOVA table** | **SS (Type III)** | **DF** | **MS** | **F (DFn, DFd)** | **P value** | **% of total variation** |
| --- | --- | --- | --- | --- | --- | --- |
| Genotype x Treatment | 0.9061 | 1 | 0.9061 | F (1, 44) = 0.3114 | P=0.5797 | 0.5508 |
| Genotype | 7.341 | 1 | 7.341 | F (1, 44) = 2.523 | P=0.1194 | 4.463 |
| Treatment | 31.48 | 1 | 31.48 | F (1, 44) = 10.82 | P=0.0020 | 19.14 |

| **Šídák's multiple comparisons test** | **Predicted (LS) mean diff.** | **95.00% CI of**  **diff.** | **Individual**  **P Value** |
| --- | --- | --- | --- |
| WT :Vehicle vs. WT :SNAP | -1.455 | -3.253 to 0.3437 | 0.1723 |
| WT :Vehicle vs. KO:Vehicle | -0.5488 | -2.259 to 1.162 | 0.9440 |
| WT :Vehicle vs. KO:SNAP | -2.598 | -4.813 to -0.3829 | 0.0140 |
| WT :SNAP vs. KO:Vehicle | 0.9059 | -1.019 to 2.831 | 0.7411 |
| WT :SNAP vs. KO:SNAP | -1.143 | -3.528 to 1.242 | 0.7248 |
| KO:Vehicle vs. KO:SNAP | -2.049 | -4.369 to 0.2700 | 0.1091 |

Resting EEG AuC High Gamma

| **ANOVA table** | **SS (Type III)** | **DF** | **MS** | **F (DFn, DFd)** | **P value** | **% of total variation** |
| --- | --- | --- | --- | --- | --- | --- |
| Genotype x Treatment | 6.856 | 1 | 6.856 | F (1, 44) = 2.776 | P=0.1028 | 4.743 |
| Genotype | 3.025 | 1 | 3.025 | F (1, 44) = 1.225 | P=0.2745 | 2.092 |
| Treatment | 34.07 | 1 | 34.07 | F (1, 44) = 13.79 | P=0.0006 | 23.57 |

| **Šídák's multiple comparisons test** | **Predicted (LS) mean diff.** | **95.00% CI of**  **diff.** | **Individual**  **P Value** |
| --- | --- | --- | --- |
| WT :Vehicle vs. WT :SNAP | -1.005 | -2.662 to 0.6519 | 0.4751 |
| WT :Vehicle vs. KO:Vehicle | 0.2746 | -1.301 to 1.850 | 0.9976 |
| WT :Vehicle vs. KO:SNAP | -2.366 | -4.407 to -0.3248 | 0.0155 |
| WT :SNAP vs. KO:Vehicle | 1.280 | -0.4941 to 3.053 | 0.2794 |
| WT :SNAP vs. KO:SNAP | -1.361 | -3.558 to 0.8365 | 0.4509 |
| KO:Vehicle vs. KO:SNAP | -2.640 | -4.777 to -0.5035 | 0.0085 |

Statistics for Figure 6F

Resting EEG FC Delta

| **ANOVA table** | **SS (Type III)** | **DF** | **MS** | **F (DFn, DFd)** | **P value** | **% of total variation** |
| --- | --- | --- | --- | --- | --- | --- |
| Genotype x Treatment | 97603 | 1 | 97603 | F (1, 45) = 1.381 | P=0.2461 | 2.732 |
| Genotype | 4367 | 1 | 4367 | F (1, 45) = 0.06180 | P=0.8048 | 0.1222 |
| Treatment | 189601 | 1 | 189601 | F (1, 45) = 2.683 | P=0.1084 | 5.306 |

Resting EEG FC Theta

| **ANOVA table** | **SS (Type III)** | **DF** | **MS** | **F (DFn, DFd)** | **P value** | **% of total variation** |
| --- | --- | --- | --- | --- | --- | --- |
| Genotype x Treatment | 12900 | 1 | 12900 | F (1, 45) = 1.743 | P=0.1935 | 3.626 |
| Genotype | 2760 | 1 | 2760 | F (1, 45) = 0.3729 | P=0.5445 | 0.7758 |
| Treatment | 1487 | 1 | 1487 | F (1, 45) = 0.2008 | P=0.6562 | 0.4178 |

Resting EEG FC Alpha

| **ANOVA table** | **SS (Type III)** | **DF** | **MS** | **F (DFn, DFd)** | **P value** | **% of total variation** |
| --- | --- | --- | --- | --- | --- | --- |
| Genotype x Treatment | 89.03 | 1 | 89.03 | F (1, 44) = 0.1326 | P=0.7175 | 0.2907 |
| Genotype | 513.9 | 1 | 513.9 | F (1, 44) = 0.7652 | P=0.3865 | 1.678 |
| Treatment | 214.5 | 1 | 214.5 | F (1, 44) = 0.3194 | P=0.5749 | 0.7004 |

Resting EEG FC Low Gamma

| **ANOVA table** | **SS (Type III)** | **DF** | **MS** | **F (DFn, DFd)** | **P value** | **% of total variation** |
| --- | --- | --- | --- | --- | --- | --- |
| Genotype x Treatment | 0.003391 | 1 | 0.003391 | F (1, 45) = 0.001971 | P=0.9648 | 0.002845 |
| Genotype | 3.180 | 1 | 3.180 | F (1, 45) = 1.848 | P=0.1808 | 2.668 |
| Treatment | 37.34 | 1 | 37.34 | F (1, 45) = 21.70 | P<0.0001 | 31.33 |

| **Šídák's multiple comparisons test** | **Predicted (LS) mean diff.** | **95.00% CI of**  **diff.** | **Individual**  **P Value** |
| --- | --- | --- | --- |
| WT :Vehicle vs. WT :SNAP | -1.913 | -3.294 to -0.5312 | 0.0025 |
| WT :Vehicle vs. KO:Vehicle | -0.5710 | -1.857 to 0.7154 | 0.7888 |
| WT :Vehicle vs. KO:SNAP | -2.447 | -4.149 to -0.7459 | 0.0016 |
| WT :SNAP vs. KO:Vehicle | 1.342 | -0.1127 to 2.796 | 0.0848 |
| WT :SNAP vs. KO:SNAP | -0.5348 | -2.367 to 1.297 | 0.9642 |
| KO:Vehicle vs. KO:SNAP | -1.877 | -3.638 to -0.1152 | 0.0313 |

Resting EEG FC High Gamma

| **ANOVA table** | **SS (Type III)** | **DF** | **MS** | **F (DFn, DFd)** | **P value** | **% of total variation** |
| --- | --- | --- | --- | --- | --- | --- |
| Genotype x Treatment | 0.07274 | 1 | 0.07274 | F (1, 44) = 0.09811 | P=0.7556 | 0.1300 |
| Genotype | 0.5640 | 1 | 0.5640 | F (1, 44) = 0.7607 | P=0.3878 | 1.008 |
| Treatment | 22.57 | 1 | 22.57 | F (1, 44) = 30.44 | P<0.0001 | 40.34 |

| **Šídák's multiple comparisons test** | **Predicted (LS) mean diff.** | **95.00% CI of**  **diff.** | **Individual**  **P Value** |
| --- | --- | --- | --- |
| WT :Vehicle vs. WT :SNAP | -1.395 | -2.313 to -0.4775 | 0.0008 |
| WT :Vehicle vs. KO:Vehicle | -0.1499 | -1.006 to 0.7062 | 0.9975 |
| WT :Vehicle vs. KO:SNAP | -1.713 | -2.839 to -0.5868 | 0.0008 |
| WT :SNAP vs. KO:Vehicle | 1.245 | 0.2898 to 2.201 | 0.0050 |
| WT :SNAP vs. KO:SNAP | -0.3178 | -1.522 to 0.8859 | 0.9781 |
| KO:Vehicle vs. KO:SNAP | -1.563 | -2.721 to -0.4059 | 0.0034 |

Statistics for Figure S4C

Resting EEG AuC Delta

| Mixed-effects model (REML) | Matching: Across row |  |
| --- | --- | --- |
| Assume sphericity? | Yes |  |
| Alpha | 0.05 |  |
| Fixed effects (type III) | P value | F (DFn, DFd) |
| Genotype | 0.0843 | F (1, 25) = 3.231 |
| Treatment | 0.1207 | F (1, 20) = 2.628 |
| Genotype x Treatment | 0.6578 | F (1, 20) = 0.2021 |
| Random effects | SD | Variance |
| Genotype | 55.24 | 3051 |
| Residual | 58.23 | 3391 |

Resting EEG AuC Theta

| Mixed-effects model (REML) | Matching: Across row |  |
| --- | --- | --- |
| Assume sphericity? | Yes |  |
| Alpha | 0.05 |  |
| Fixed effects (type III) | P value | F (DFn, DFd) |
| Genotype | 0.7406 | "F (1, 25) = 0.1121" |
| Treatment | 0.0378 | "F (1, 20) = 4.945" |
| Genotype x Treatment | 0.5456 | "F (1, 20) = 0.3780" |
| Random effects | SD | Variance |
| Genotype | 25.43 | 646.7 |
| Residual | 33.45 | 1119 |

Resting EEG AuC Alpha

| Mixed-effects model (REML) | Matching: Across row |  |
| --- | --- | --- |
| Assume sphericity? | Yes |  |
| Alpha | 0.05 |  |
| Fixed effects (type III) | P value | F (DFn, DFd) |
| Genotype | 0.4570 | "F (1, 25) = 0.5709" |
| Treatment | 0.0393 | "F (1, 19) = 4.899" |
| Genotype x Treatment | 0.7191 | "F (1, 19) = 0.1332" |
| Random effects | SD | Variance |
| Genotype | 11.63 | 135.2 |
| Residual | 9.952 | 99.04 |

Resting EEG AuC Beta

| Mixed-effects model (REML) | Matching: Across row |  |
| --- | --- | --- |
| Assume sphericity? | Yes |  |
| Alpha | 0.05 |  |
| Fixed effects (type III) | P value | F (DFn, DFd) |
| Genotype | 0.1904 | "F (1, 25) = 1.812" |
| Treatment | 0.4304 | "F (1, 21) = 0.6464" |
| Genotype x Treatment | 0.2858 | "F (1, 21) = 1.200" |
| Random effects | SD | Variance |
| Genotype | 9.525 | 90.72 |
| Residual | 8.872 | 78.72 |

Resting EEG AuC Gamma

| Mixed-effects model (REML) | Matching: Across row |  |
| --- | --- | --- |
| Assume sphericity? | Yes |  |
| Alpha | 0.05 |  |
| Fixed effects (type III) | P value | F (DFn, DFd) |
| Genotype | 0.5307 | "F (1, 25) = 0.4042" |
| Treatment | 0.7923 | "F (1, 22) = 0.07102" |
| Genotype x Treatment | 0.6802 | "F (1, 22) = 0.1744" |
| Random effects | SD | Variance |
| Genotype | 0.8000 | 0.6400 |
| Residual | 0.4786 | 0.2290 |

Resting EEG AuC Low Gamma

| Mixed-effects model (REML) | Matching: Across row |  |
| --- | --- | --- |
| Assume sphericity? | Yes |  |
| Alpha | 0.05 |  |
| Fixed effects (type III) | P value | F (DFn, DFd) |
| Genotype | 0.7608 | "F (1, 25) = 0.09476" |
| Treatment | 0.5586 | "F (1, 22) = 0.3528" |
| Genotype x Treatment | 0.7120 | "F (1, 22) = 0.1399" |
| Random effects | SD | Variance |
| Genotype | 1.773 | 3.144 |
| Residual | 0.9417 | 0.8868 |

Resting EEG AuC High Gamma

| Mixed-effects model (REML) | Matching: Across row |  |
| --- | --- | --- |
| Assume sphericity? | Yes |  |
| Alpha | 0.05 |  |
| Fixed effects (type III) | P value | F (DFn, DFd) |
| Genotype | 0.1646 | "F (1, 25) = 2.050" |
| Treatment | 0.0137 | "F (1, 23) = 7.127" |
| Genotype x Treatment | 0.7191 | "F (1, 23) = 0.1325" |
| Random effects | SD | Variance |
| Genotype | 0.2998 | 0.08988 |
| Residual | 0.2411 | 0.05813 |

| **Bonferroni's multiple comparisons test** | **Predicted (LS) mean diff.** | **95.00% CI of**  **diff.** | **Individual**  **P Value** |
| --- | --- | --- | --- |
| Ctrl WT |  |  |  |
| Untreated vs. SNAP | -0.1562 | -0.3889 to 0.07649 | 0.2422 |
| cKO |  |  |  |
| Untreated  vs. SNAP | -0.2055 | -0.4323 to 0.02123 | 0.0806 |
| Untreated |  |  |  |
| Ctrl WT vs. cKO | -0.1670 | -0.5099 to 0.1758 | 0.5305 |
| SNAP |  |  |  |
| Ctrl WT vs. cKO | -0.2164 | -0.5678 to 0.1351 | 0.3215 |

Statistics for Figure S4D

Resting EEG FC Delta

| Mixed-effects model (REML) | Matching: Across row |  |
| --- | --- | --- |
| Assume sphericity? | Yes |  |
| Alpha | 0.05 |  |
| Fixed effects (type III) | P value | F (DFn, DFd) |
| Genotype | 0.5610 | "F (1, 25) = 0.3472" |
| Treatment | 0.0002 | "F (1, 20) = 19.78" |
| Genotype x Treatment | 0.7514 | "F (1, 20) = 0.1032" |
| Random effects | SD | Variance |
| Genotype | 69.86 | 4880 |
| Residual | 37.41 | 1399 |

| **Bonferroni's multiple comparisons test** | **Predicted (LS) mean diff.** | **95.00% CI of**  **diff.** | **Individual**  **P Value** |
| --- | --- | --- | --- |
| Ctrl WT |  |  |  |
| Untreated vs. SNAP | 53.19 | 15.01 to 91.38 | 0.0060 |
| cKO |  |  |  |
| Untreated  vs. SNAP | 46.03 | 7.761 to 84.29 | 0.0171 |
| Untreated |  |  |  |
| Ctrl WT vs. cKO | -13.57 | -84.95 to 57.81 | >0.9999 |
| SNAP |  |  |  |
| Ctrl WT vs. cKO | -20.74 | -93.92 to 52.45 | >0.9999 |

Resting EEG FC Theta

| Mixed-effects model (REML) | Matching: Across row |  |
| --- | --- | --- |
| Assume sphericity? | Yes |  |
| Alpha | 0.05 |  |
| Fixed effects (type III) | P value | F (DFn, DFd) |
| Genotype | 0.4256 | "F (1, 25) = 0.6562" |
| Treatment | 0.0977 | "F (1, 21) = 3.003" |
| Genotype x Treatment | 0.5050 | "F (1, 21) = 0.4600" |
| Random effects | SD | Variance |
| Genotype | 33.71 | 1136 |
| Residual | 45.90 | 2106 |

Resting EEG FC Alpha

| Mixed-effects model (REML) | Matching: Across row |  |
| --- | --- | --- |
| Assume sphericity? | Yes |  |
| Alpha | 0.05 |  |
| Fixed effects (type III) | P value | F (DFn, DFd) |
| Genotype | 0.8962 | "F (1, 25) = 0.01736" |
| Treatment | 0.1632 | "F (1, 21) = 2.089" |
| Genotype x Treatment | 0.9586 | "F (1, 21) = 0.002764" |
| Random effects | SD | Variance |
| Genotype | 10.91 | 119.0 |
| Residual | 13.60 | 184.9 |

Resting EEG FC Beta

| Mixed-effects model (REML) | Matching: Across row |  |
| --- | --- | --- |
| Assume sphericity? | Yes |  |
| Alpha | 0.05 |  |
| Fixed effects (type III) | P value | F (DFn, DFd) |
| Genotype | 0.2470 | "F (1, 25) = 1.405" |
| Treatment | 0.8721 | "F (1, 20) = 0.02658" |
| Genotype x Treatment | 0.7111 | "F (1, 20) = 0.1412" |
| Random effects | SD | Variance |
| Genotype | 8.561 | 73.29 |
| Residual | 6.863 | 47.10 |

Resting EEG FC Gamma

| Mixed-effects model (REML) | Matching: Across row |  |
| --- | --- | --- |
| Assume sphericity? | Yes |  |
| Alpha | 0.05 |  |
| Fixed effects (type III) | P value | F (DFn, DFd) |
| Genotype | 0.0133 | "F (1, 25) = 7.092" |
| Treatment | 0.9497 | "F (1, 22) = 0.004066" |
| Genotype x Treatment | 0.3389 | "F (1, 22) = 0.9557" |
| Random effects | SD | Variance |
| Genotype | 0.1806 | 0.03263 |
| Residual | 0.6077 | 0.3693 |

| **Bonferroni's multiple comparisons test** | **Predicted (LS) mean diff.** | **95.00% CI of**  **diff.** | **Individual**  **P Value** |
| --- | --- | --- | --- |
| Ctrl WT |  |  |  |
| Untreated vs. SNAP | 0.1784 | -0.4135 to 0.7702 | 0.9523 |
| cKO |  |  |  |
| Untreated  vs. SNAP | -0.1565 | -0.7299 to 0.4168 | >0.9999 |
| Untreated |  |  |  |
| Ctrl WT vs. cKO | -0.3249 | -0.8903 to 0.2405 | 0.3796 |
| SNAP |  |  |  |
| Ctrl WT vs. cKO | -0.6597 | -1.261 to -0.05861 | 0.0288 |

Resting EEG FC Low Gamma

| Mixed-effects model (REML) | Matching: Across row |  |
| --- | --- | --- |
| Assume sphericity? | Yes |  |
| Alpha | 0.05 |  |
| Fixed effects (type III) | P value | F (DFn, DFd) |
| Genotype | 0.0669 | "F (1, 25) = 3.671" |
| Treatment | 0.2863 | "F (1, 22) = 1.194" |
| Genotype x Treatment | 0.3482 | "F (1, 22) = 0.9187" |
| Random effects | SD | Variance |
| Genotype | 0.7075 | 0.5006 |
| Residual | 1.056 | 1.116 |

Resting EEG FC High Gamma

| Mixed-effects model (REML) | Matching: Across row |  |
| --- | --- | --- |
| Assume sphericity? | Yes |  |
| Alpha | 0.05 |  |
| Fixed effects (type III) | P value | F (DFn, DFd) |
| Genotype | 0.0075 | "F (1, 25) = 8.451" |
| Treatment | 0.0666 | "F (1, 23) = 3.709" |
| Genotype x Treatment | 0.3527 | "F (1, 23) = 0.9000" |
| Random effects | SD | Variance |
| Genotype | 0.05590 | 0.003125 |
| Residual | 0.4032 | 0.1625 |

| **Bonferroni's multiple comparisons test** | **Predicted (LS) mean diff.** | **95.00% CI of**  **diff.** | **Individual**  **P Value** |
| --- | --- | --- | --- |
| Ctrl WT |  |  |  |
| Untreated vs. SNAP | -0.1095 | -0.4901 to 0.2711 | 0.9945 |
| cKO |  |  |  |
| Untreated  vs. SNAP | -0.3220 | -0.7012 to 0.05716 | 0.1068 |
| Untreated |  |  |  |
| Ctrl WT vs. cKO | -0.2254 | -0.5881 to 0.1374 | 0.3142 |
| SNAP |  |  |  |
| Ctrl WT vs. cKO | -0.4379 | -0.8149 to -0.06088 | 0.0197 |

Statistics for Figure S4E

Resting EEG Power Coupling A1A2

| Mixed-effects model (REML) | Matching: Across row |  |
| --- | --- | --- |
| Assume sphericity? | Yes |  |
| Alpha | 0.05 |  |
| Fixed effects (type III) | P value | F (DFn, DFd) |
| Genotype | 0.1087 | "F (1, 24) = 2.775" |
| Treatment | 0.5037 | "F (1, 23) = 0.4614" |
| Genotype x Treatment | 0.7014 | "F (1, 23) = 0.1507" |
| Random effects | SD | Variance |
| Genotype | 0.1160 | 0.01347 |
| Residual | 0.2059 | 0.04241 |

Resting EEG Power Coupling A1G1

| Mixed-effects model (REML) | Matching: Across row |  |
| --- | --- | --- |
| Assume sphericity? | Yes |  |
| Alpha | 0.05 |  |
| Fixed effects (type III) | P value | F (DFn, DFd) |
| Genotype | 0.1943 | "F (1, 48) = 1.733" |
| Treatment | 0.6402 | "F (1, 48) = 0.2212" |
| Genotype x Treatment | 0.1113 | "F (1, 48) = 2.632" |
| Random effects | SD | Variance |
| Genotype | 0.000 | 0.000 |
| Residual | 0.2004 | 0.04017 |

Resting EEG Power Coupling A1G2

| Mixed-effects model (REML) | Matching: Across row |  |
| --- | --- | --- |
| Assume sphericity? | Yes |  |
| Alpha | 0.05 |  |
| Fixed effects (type III) | P value | F (DFn, DFd) |
| Genotype | 0.2120 | "F (1, 47) = 1.601" |
| Treatment | 0.9224 | "F (1, 47) = 0.009601" |
| Genotype x Treatment | 0.1856 | "F (1, 47) = 1.805" |
| Random effects | SD | Variance |
| Genotype | 0.000 | 0.000 |
| Residual | 0.1923 | 0.03699 |

Resting EEG Power Coupling A2G1

| Mixed-effects model (REML) | Matching: Across row |  |
| --- | --- | --- |
| Assume sphericity? | Yes |  |
| Alpha | 0.05 |  |
| Fixed effects (type III) | P value | F (DFn, DFd) |
| Genotype | 0.7139 | "F (1, 48) = 0.1360" |
| Treatment | 0.6980 | "F (1, 48) = 0.1523" |
| Genotype x Treatment | 0.9433 | "F (1, 48) = 0.005108" |
| Random effects | SD | Variance |
| Genotype | 0.000 | 0.000 |
| Residual | 0.2304 | 0.05311 |

Resting EEG Power Coupling A2G2

| Mixed-effects model (REML) | Matching: Across row |  |
| --- | --- | --- |
| Assume sphericity? | Yes |  |
| Alpha | 0.05 |  |
| Fixed effects (type III) | P value | F (DFn, DFd) |
| Genotype | 0.7856 | "F (1, 47) = 0.07482" |
| Treatment | 0.7915 | "F (1, 47) = 0.07068" |
| Genotype x Treatment | 0.9768 | "F (1, 47) = 0.0008511" |
| Random effects | SD | Variance |
| Genotype | 0.000 | 0.000 |
| Residual | 0.2056 | 0.04229 |

Statistics for Figure S4F

Resting EEG Power Coupling D1D2

| Mixed-effects model (REML) | Matching: Across row |  |
| --- | --- | --- |
| Assume sphericity? | Yes |  |
| Alpha | 0.05 |  |
| Fixed effects (type III) | P value | F (DFn, DFd) |
| Genotype | 0.5810 | "F (1, 24) = 0.3130" |
| Treatment | 0.0136 | "F (1, 23) = 7.149" |
| Genotype x Treatment | 0.6674 | "F (1, 23) = 0.1894" |
| Random effects | SD | Variance |
| Genotype | 0.1370 | 0.01876 |
| Residual | 0.1616 | 0.02612 |

| **Bonferroni's multiple comparisons test** | **Predicted (LS) mean diff.** | **95.00% CI of**  **diff.** | **Individual**  **P Value** |
| --- | --- | --- | --- |
| Ctrl WT |  |  |  |
| Untreated vs. SNAP | 0.1414 | -0.01503 to 0.2978 | 0.0816 |
| cKO |  |  |  |
| Untreated vs. SNAP | 0.1018 | -0.05019 to 0.2538 | 0.2438 |
| Untreated |  |  |  |
| Ctrl WT vs. cKO | -0.01958 | -0.2120 to 0.1728 | >0.9999 |
| SNAP |  |  |  |
| Ctrl WT vs. cKO | -0.05917 | -0.2548 to 0.1365 | 0.9745 |

Resting EEG Power Coupling D1G1

| Mixed-effects model (REML) | Matching: Across row |  |
| --- | --- | --- |
| Assume sphericity? | Yes |  |
| Alpha | 0.05 |  |
| Fixed effects (type III) | P value | F (DFn, DFd) |
| Genotype | 0.1751 | "F (1, 25) = 1.948" |
| Treatment | 0.0729 | "F (1, 22) = 3.548" |
| Genotype x Treatment | 0.5842 | "F (1, 22) = 0.3085" |
| Random effects | SD | Variance |
| Genotype | 0.06846 | 0.004686 |
| Residual | 0.2034 | 0.04138 |

Resting EEG Power Coupling D1G2

| Mixed-effects model (REML) | Matching: Across row |  |
| --- | --- | --- |
| Assume sphericity? | Yes |  |
| Alpha | 0.05 |  |
| Fixed effects (type III) | P value | F (DFn, DFd) |
| Genotype | 0.2647 | "F (1, 25) = 1.302" |
| Treatment | 0.0349 | "F (1, 22) = 5.053" |
| Genotype x Treatment | 0.3451 | "F (1, 22) = 0.9309" |
| Random effects | SD | Variance |
| Genotype | 0.09433 | 0.008898 |
| Residual | 0.1970 | 0.03880 |

| **Bonferroni's multiple comparisons test** | **Predicted (LS) mean diff.** | **95.00% CI of**  **diff.** | **Individual**  **P Value** |
| --- | --- | --- | --- |
| Ctrl WT |  |  |  |
| Untreated vs. SNAP | -0.07124 | -0.2631 to 0.1206 | 0.7627 |
| cKO |  |  |  |
| Untreated vs. SNAP | -0.1784 | -0.3642 to 0.007480 | 0.0615 |
| Untreated |  |  |  |
| Ctrl WT vs. cKO | 0.1292 | -0.06904 to 0.3274 | 0.2759 |
| SNAP |  |  |  |
| Ctrl WT vs. cKO | 0.02206 | -0.1801 to 0.2243 | >0.9999 |

Resting EEG Power Coupling D2G1

| Mixed-effects model (REML) | Matching: Across row |  |
| --- | --- | --- |
| Assume sphericity? | Yes |  |
| Alpha | 0.05 |  |
| Fixed effects (type III) | P value | F (DFn, DFd) |
| Genotype | 0.3058 | "F (1, 48) = 1.071" |
| Treatment | 0.3165 | "F (1, 48) = 1.024" |
| Genotype x Treatment | 0.2989 | "F (1, 48) = 1.103" |
| Random effects | SD | Variance |
| Genotype | 0.000 | 0.000 |
| Residual | 0.2166 | 0.04692 |

Resting EEG Power Coupling D2G2

| Mixed-effects model (REML) | Matching: Across row |  |
| --- | --- | --- |
| Assume sphericity? | Yes |  |
| Alpha | 0.05 |  |
| Fixed effects (type III) | P value | F (DFn, DFd) |
| Genotype | 0.2274 | "F (1, 48) = 1.495" |
| Treatment | 0.0618 | "F (1, 48) = 3.659" |
| Genotype x Treatment | 0.5245 | "F (1, 48) = 0.4109" |
| Random effects | SD | Variance |
| Genotype | 0.000 | 0.000 |
| Residual | 0.2611 | 0.06818 |

Statistics for Figure S4G

Resting EEG Power Coupling A1A2

| **ANOVA table** | **SS (Type III)** | **DF** | **MS** | **F (DFn, DFd)** | **P value** | **% of total variation** |
| --- | --- | --- | --- | --- | --- | --- |
| Genotype x Treatment | 0.003672 | 2 | 0.001836 | F (2, 119) = 0.03704 | P=0.9636 | 0.1820 |
| Genotype | 0.003661 | 1 | 0.003661 | F (1, 119) = 0.07386 | P=0.7863 | 0.1936 |
| Treatment | 0.8716 | 2 | 0.4358 | F (2, 119) = 8.793 | P=0.0003 | 13.27 |

| **Bonferroni's multiple comparisons test** | **Predicted (LS) mean diff.** | **95.00% CI of**  **diff.** | **Individual**  **P Value** |
| --- | --- | --- | --- |
| WT :Pre vs. WT :Vehicle | -0.05028 | -0.2375 to 0.1369 | >0.9999 |
| WT :Pre vs. WT :SNAP | 0.2271 | 0.001722 to 0.4524 | 0.0466 |
| WT :Pre vs. KO:Pre | -0.02803 | -0.1824 to 0.1263 | >0.9999 |
| WT :Pre vs. KO:Vehicle | -0.05489 | -0.2601 to 0.1503 | >0.9999 |
| WT :Pre vs. KO:SNAP | 0.2196 | -0.07100 to 0.5103 | 0.3809 |
| WT :Vehicle vs. WT :SNAP | 0.2773 | 0.02211 to 0.5326 | 0.0221 |
| WT :Vehicle vs. KO:Pre | 0.02226 | -0.1732 to 0.2177 | >0.9999 |
| WT :Vehicle vs. KO:Vehicle | -0.004602 | -0.2422 to 0.2330 | >0.9999 |
| WT :Vehicle vs. KO:SNAP | 0.2699 | -0.04446 to 0.5843 | 0.1701 |
| WT :SNAP vs. KO:Pre | -0.2551 | -0.4873 to -0.02290 | 0.0197 |
| WT :SNAP vs. KO:Vehicle | -0.2819 | -0.5506 to -0.01324 | 0.0316 |
| WT :SNAP vs. KO:SNAP | -0.007418 | -0.3459 to 0.3310 | >0.9999 |
| KO:Pre vs. KO:Vehicle | -0.02686 | -0.2396 to 0.1859 | >0.9999 |
| KO:Pre vs. KO:SNAP | 0.2477 | -0.04831 to 0.5436 | 0.2031 |
| KO:Vehicle vs. KO:SNAP | 0.2745 | -0.05089 to 0.5999 | 0.1922 |

Resting EEG Power Coupling A1G1

| **ANOVA table** | **SS (Type III)** | **DF** | **MS** | **F (DFn, DFd)** | **P value** | **% of total variation** |
| --- | --- | --- | --- | --- | --- | --- |
| Genotype x Treatment | 0.2045 | 2 | 0.1023 | F (2, 119) = 1.801 | P=0.1696 | 2.669 |
| Genotype | 0.04887 | 1 | 0.04887 | F (1, 119) = 0.8606 | P=0.3554 | 0.6377 |
| Treatment | 0.2817 | 2 | 0.1409 | F (2, 119) = 2.481 | P=0.0880 | 3.677 |

Resting EEG Power Coupling A1G2

| **ANOVA table** | **SS (Type III)** | **DF** | **MS** | **F (DFn, DFd)** | **P value** | **% of total variation** |
| --- | --- | --- | --- | --- | --- | --- |
| Genotype x Treatment | 0.1718 | 2 | 0.08592 | F (2, 119) = 1.442 | P=0.2406 | 2.260 |
| Genotype | 3.123e-006 | 1 | 3.123e-006 | F (1, 119) = 5.240e-005 | P=0.9942 | 4.107e-005 |
| Treatment | 0.1848 | 2 | 0.09242 | F (2, 119) = 1.551 | P=0.2164 | 2.431 |

Resting EEG Power Coupling A2G1

| **ANOVA table** | **SS (Type III)** | **DF** | **MS** | **F (DFn, DFd)** | **P value** | **% of total variation** |
| --- | --- | --- | --- | --- | --- | --- |
| Genotype x Treatment | 0.03262 | 2 | 0.01631 | F (2, 119) = 0.3972 | P=0.6731 | 0.5818 |
| Genotype | 0.1671 | 1 | 0.1671 | F (1, 119) = 4.070 | P=0.0459 | 2.981 |
| Treatment | 0.2767 | 2 | 0.1384 | F (2, 119) = 3.370 | P=0.0377 | 4.936 |

| **Bonferroni's multiple comparisons test** | **Predicted (LS) mean diff.** | **95.00% CI of**  **diff.** | **Individual**  **P Value** |
| --- | --- | --- | --- |
| WT :Pre vs. WT :Vehicle | -0.1223 | -0.2927 to 0.04814 | 0.5044 |
| WT :Pre vs. WT :SNAP | -0.1437 | -0.3488 to 0.06141 | 0.5694 |
| WT :Pre vs. KO:Pre | -0.1366 | -0.2771 to 0.003877 | 0.0642 |
| WT :Pre vs. KO:Vehicle | -0.2092 | -0.3960 to -0.02241 | 0.0160 |
| WT :Pre vs. KO:SNAP | -0.1908 | -0.4553 to 0.07378 | 0.4915 |
| WT :Vehicle vs. WT :SNAP | -0.02142 | -0.2537 to 0.2109 | >0.9999 |
| WT :Vehicle vs. KO:Pre | -0.01433 | -0.1922 to 0.1635 | >0.9999 |
| WT :Vehicle vs. KO:Vehicle | -0.08693 | -0.3032 to 0.1294 | >0.9999 |
| WT :Vehicle vs. KO:SNAP | -0.06848 | -0.3546 to 0.2177 | >0.9999 |
| WT :SNAP vs. KO:Pre | 0.007086 | -0.2042 to 0.2184 | >0.9999 |
| WT :SNAP vs. KO:Vehicle | -0.06551 | -0.3101 to 0.1791 | >0.9999 |
| WT :SNAP vs. KO:SNAP | -0.04707 | -0.3551 to 0.2610 | >0.9999 |
| KO:Pre vs. KO:Vehicle | -0.07259 | -0.2662 to 0.1210 | >0.9999 |
| KO:Pre vs. KO:SNAP | -0.05415 | -0.3236 to 0.2152 | >0.9999 |
| KO:Vehicle vs. KO:SNAP | 0.01844 | -0.2777 to 0.3146 | >0.9999 |

Resting EEG Power Coupling A2G2

| **ANOVA table** | **SS (Type III)** | **DF** | **MS** | **F (DFn, DFd)** | **P value** | **% of total variation** |
| --- | --- | --- | --- | --- | --- | --- |
| Genotype x Treatment | 0.02268 | 2 | 0.01134 | F (2, 119) = 0.2215 | P=0.8017 | 0.3315 |
| Genotype | 0.1819 | 1 | 0.1819 | F (1, 119) = 3.553 | P=0.0619 | 2.659 |
| Treatment | 0.3510 | 2 | 0.1755 | F (2, 119) = 3.428 | P=0.0357 | 5.131 |

| **Bonferroni's multiple comparisons test** | **Predicted (LS) mean diff.** | **95.00% CI of**  **diff.** | **Individual**  **P Value** |
| --- | --- | --- | --- |
| WT :Pre vs. WT :Vehicle | -0.1547 | -0.3450 to 0.03557 | 0.2452 |
| WT :Pre vs. WT :SNAP | -0.08529 | -0.3143 to 0.1437 | >0.9999 |
| WT :Pre vs. KO:Pre | -0.1280 | -0.2849 to 0.02889 | 0.2399 |
| WT :Pre vs. KO:Vehicle | -0.2210 | -0.4296 to -0.01242 | 0.0287 |
| WT :Pre vs. KO:SNAP | -0.1734 | -0.4688 to 0.1220 | >0.9999 |
| WT :Vehicle vs. WT :SNAP | 0.06944 | -0.1900 to 0.3289 | >0.9999 |
| WT :Vehicle vs. KO:Pre | 0.02674 | -0.1719 to 0.2254 | >0.9999 |
| WT :Vehicle vs. KO:Vehicle | -0.06627 | -0.3078 to 0.1753 | >0.9999 |
| WT :Vehicle vs. KO:SNAP | -0.01864 | -0.3382 to 0.3009 | >0.9999 |
| WT :SNAP vs. KO:Pre | -0.04270 | -0.2787 to 0.1933 | >0.9999 |
| WT :SNAP vs. KO:Vehicle | -0.1357 | -0.4088 to 0.1374 | >0.9999 |
| WT :SNAP vs. KO:SNAP | -0.08808 | -0.4321 to 0.2559 | >0.9999 |
| KO:Pre vs. KO:Vehicle | -0.09301 | -0.3092 to 0.1232 | >0.9999 |
| KO:Pre vs. KO:SNAP | -0.04538 | -0.3462 to 0.2555 | >0.9999 |
| KO:Vehicle vs. KO:SNAP | 0.04763 | -0.2831 to 0.3784 | >0.9999 |

Statistics for Figure S4H

Resting EEG Power Coupling D1D2

| **ANOVA table** | **SS (Type III)** | **DF** | **MS** | **F (DFn, DFd)** | **P value** | **% of total variation** |
| --- | --- | --- | --- | --- | --- | --- |
| Genotype x Treatment | 0.003220 | 2 | 0.001610 | F (2, 119) = 0.02772 | P=0.9727 | 0.04304 |
| Genotype | 0.03863 | 1 | 0.03863 | F (1, 119) = 0.6651 | P=0.4164 | 0.5163 |
| Treatment | 0.4592 | 2 | 0.2296 | F (2, 119) = 3.954 | P=0.0218 | 6.139 |

| **Bonferroni's multiple comparisons test** | **Predicted (LS) mean diff.** | **95.00% CI of**  **diff.** | **Individual**  **P Value** |
| --- | --- | --- | --- |
| WT :Pre vs. WT :Vehicle | -0.05762 | -0.2603 to 0.1450 | >0.9999 |
| WT :Pre vs. WT :SNAP | 0.1644 | -0.07953 to 0.4083 | 0.6862 |
| WT :Pre vs. KO:Pre | -0.03052 | -0.1976 to 0.1365 | >0.9999 |
| WT :Pre vs. KO:Vehicle | -0.09507 | -0.3172 to 0.1271 | >0.9999 |
| WT :Pre vs. KO:SNAP | 0.1023 | -0.2123 to 0.4169 | >0.9999 |
| WT :Vehicle vs. WT :SNAP | 0.2220 | -0.05427 to 0.4983 | 0.2642 |
| WT :Vehicle vs. KO:Pre | 0.02710 | -0.1844 to 0.2386 | >0.9999 |
| WT :Vehicle vs. KO:Vehicle | -0.03745 | -0.2947 to 0.2198 | >0.9999 |
| WT :Vehicle vs. KO:SNAP | 0.1599 | -0.1804 to 0.5002 | >0.9999 |
| WT :SNAP vs. KO:Pre | -0.1949 | -0.4463 to 0.05643 | 0.3281 |
| WT :SNAP vs. KO:Vehicle | -0.2595 | -0.5503 to 0.03141 | 0.1289 |
| WT :SNAP vs. KO:SNAP | -0.06211 | -0.4285 to 0.3043 | >0.9999 |
| KO:Pre vs. KO:Vehicle | -0.06455 | -0.2948 to 0.1657 | >0.9999 |
| KO:Pre vs. KO:SNAP | 0.1328 | -0.1876 to 0.4532 | >0.9999 |
| KO:Vehicle vs. KO:SNAP | 0.1974 | -0.1549 to 0.5496 | >0.9999 |

Resting EEG Power Coupling D1G1

| **ANOVA table** | **SS (Type III)** | **DF** | **MS** | **F (DFn, DFd)** | **P value** | **% of total variation** |
| --- | --- | --- | --- | --- | --- | --- |
| Genotype x Treatment | 0.1883 | 2 | 0.09415 | F (2, 119) = 1.339 | P=0.2659 | 1.993 |
| Genotype | 0.006596 | 1 | 0.006596 | F (1, 119) = 0.09383 | P=0.7599 | 0.06982 |
| Treatment | 0.7670 | 2 | 0.3835 | F (2, 119) = 5.455 | P=0.0054 | 8.119 |

| **Bonferroni's multiple comparisons test** | **Predicted (LS) mean diff.** | **95.00% CI of**  **diff.** | **Individual**  **P Value** |
| --- | --- | --- | --- |
| WT :Pre vs. WT :Vehicle | -0.2650 | -0.4879 to -0.04199 | 0.0080 |
| WT :Pre vs. WT :SNAP | -0.1357 | -0.4040 to 0.1327 | >0.9999 |
| WT :Pre vs. KO:Pre | -0.09092 | -0.2747 to 0.09288 | >0.9999 |
| WT :Pre vs. KO:Vehicle | -0.1980 | -0.4424 to 0.04636 | 0.2506 |
| WT :Pre vs. KO:SNAP | -0.05791 | -0.4040 to 0.2882 | >0.9999 |
| WT :Vehicle vs. WT :SNAP | 0.1293 | -0.1747 to 0.4333 | >0.9999 |
| WT :Vehicle vs. KO:Pre | 0.1740 | -0.05869 to 0.4068 | 0.4040 |
| WT :Vehicle vs. KO:Vehicle | 0.06693 | -0.2161 to 0.3500 | >0.9999 |
| WT :Vehicle vs. KO:SNAP | 0.2070 | -0.1674 to 0.5815 | >0.9999 |
| WT :SNAP vs. KO:Pre | 0.04474 | -0.2318 to 0.3213 | >0.9999 |
| WT :SNAP vs. KO:Vehicle | -0.06237 | -0.3824 to 0.2576 | >0.9999 |
| WT :SNAP vs. KO:SNAP | 0.07775 | -0.3253 to 0.4808 | >0.9999 |
| KO:Pre vs. KO:Vehicle | -0.1071 | -0.3604 to 0.1462 | >0.9999 |
| KO:Pre vs. KO:SNAP | 0.03301 | -0.3195 to 0.3855 | >0.9999 |
| KO:Vehicle vs. KO:SNAP | 0.1401 | -0.2474 to 0.5277 | >0.9999 |

Resting EEG Power Coupling D1G2

| **ANOVA table** | **SS (Type III)** | **DF** | **MS** | **F (DFn, DFd)** | **P value** | **% of total variation** |
| --- | --- | --- | --- | --- | --- | --- |
| Genotype x Treatment | 0.1985 | 2 | 0.09927 | F (2, 119) = 1.344 | P=0.2648 | 1.949 |
| Genotype | 0.02617 | 1 | 0.02617 | F (1, 119) = 0.3542 | P=0.5529 | 0.2569 |
| Treatment | 1.068 | 2 | 0.5340 | F (2, 119) = 7.229 | P=0.0011 | 10.49 |

| **Bonferroni's multiple comparisons test** | **Predicted (LS) mean diff.** | **95.00% CI of**  **diff.** | **Individual**  **P Value** |
| --- | --- | --- | --- |
| WT :Pre vs. WT :Vehicle | -0.2650 | -0.4879 to -0.04199 | 0.0080 |
| WT :Pre vs. WT :SNAP | -0.1357 | -0.4040 to 0.1327 | >0.9999 |
| WT :Pre vs. KO:Pre | -0.09092 | -0.2747 to 0.09288 | >0.9999 |
| WT :Pre vs. KO:Vehicle | -0.1980 | -0.4424 to 0.04636 | 0.2506 |
| WT :Pre vs. KO:SNAP | -0.05791 | -0.4040 to 0.2882 | >0.9999 |
| WT :Vehicle vs. WT :SNAP | 0.1293 | -0.1747 to 0.4333 | >0.9999 |
| WT :Vehicle vs. KO:Pre | 0.1740 | -0.05869 to 0.4068 | 0.4040 |
| WT :Vehicle vs. KO:Vehicle | 0.06693 | -0.2161 to 0.3500 | >0.9999 |
| WT :Vehicle vs. KO:SNAP | 0.2070 | -0.1674 to 0.5815 | >0.9999 |
| WT :SNAP vs. KO:Pre | 0.04474 | -0.2318 to 0.3213 | >0.9999 |
| WT :SNAP vs. KO:Vehicle | -0.06237 | -0.3824 to 0.2576 | >0.9999 |
| WT :SNAP vs. KO:SNAP | 0.07775 | -0.3253 to 0.4808 | >0.9999 |
| KO:Pre vs. KO:Vehicle | -0.1071 | -0.3604 to 0.1462 | >0.9999 |
| KO:Pre vs. KO:SNAP | 0.03301 | -0.3195 to 0.3855 | >0.9999 |
| KO:Vehicle vs. KO:SNAP | 0.1401 | -0.2474 to 0.5277 | >0.9999 |

Resting EEG Power Coupling D2G1

| **ANOVA table** | **SS (Type III)** | **DF** | **MS** | **F (DFn, DFd)** | **P value** | **% of total variation** |
| --- | --- | --- | --- | --- | --- | --- |
| Genotype x Treatment | 0.09283 | 2 | 0.04641 | F (2, 119) = 0.8180 | P=0.4438 | 1.212 |
| Genotype | 0.01098 | 1 | 0.01098 | F (1, 119) = 0.1936 | P=0.6608 | 0.1434 |
| Treatment | 0.6520 | 2 | 0.3260 | F (2, 119) = 5.746 | P=0.0041 | 8.511 |

| **Bonferroni's multiple comparisons test** | **Predicted (LS) mean diff.** | **95.00% CI of**  **diff.** | **Individual**  **P Value** |
| --- | --- | --- | --- |
| WT :Pre vs. WT :Vehicle | -0.2209 | -0.4212 to -0.02054 | 0.0190 |
| WT :Pre vs. WT :SNAP | -0.1066 | -0.3477 to 0.1345 | >0.9999 |
| WT :Pre vs. KO:Pre | -0.1015 | -0.2666 to 0.06368 | >0.9999 |
| WT :Pre vs. KO:Vehicle | -0.2232 | -0.4427 to -0.003603 | 0.0430 |
| WT :Pre vs. KO:SNAP | -0.07224 | -0.3832 to 0.2387 | >0.9999 |
| WT :Vehicle vs. WT :SNAP | 0.1142 | -0.1589 to 0.3873 | >0.9999 |
| WT :Vehicle vs. KO:Pre | 0.1194 | -0.08968 to 0.3285 | >0.9999 |
| WT :Vehicle vs. KO:Vehicle | -0.002306 | -0.2566 to 0.2520 | >0.9999 |
| WT :Vehicle vs. KO:SNAP | 0.1486 | -0.1878 to 0.4850 | >0.9999 |
| WT :SNAP vs. KO:Pre | 0.005180 | -0.2433 to 0.2536 | >0.9999 |
| WT :SNAP vs. KO:Vehicle | -0.1165 | -0.4040 to 0.1710 | >0.9999 |
| WT :SNAP vs. KO:SNAP | 0.03440 | -0.3278 to 0.3965 | >0.9999 |
| KO:Pre vs. KO:Vehicle | -0.1217 | -0.3493 to 0.1059 | >0.9999 |
| KO:Pre vs. KO:SNAP | 0.02922 | -0.2875 to 0.3459 | >0.9999 |
| KO:Vehicle vs. KO:SNAP | 0.1401 | -0.2474 to 0.5277 | >0.9999 |

Resting EEG Power Coupling D2G2

| **ANOVA table** | **SS (Type III)** | **DF** | **MS** | **F (DFn, DFd)** | **P value** | **% of total variation** |
| --- | --- | --- | --- | --- | --- | --- |
| Genotype x Treatment | 0.1104 | 2 | 0.05522 | F (2, 119) = 0.8515 | P=0.4294 | 1.236 |
| Genotype | 0.01440 | 1 | 0.01440 | F (1, 119) = 0.2220 | P=0.6384 | 0.1612 |
| Treatment | 0.9087 | 2 | 0.4543 | F (2, 119) = 7.005 | P=0.0013 | 10.17 |

| **Bonferroni's multiple comparisons test** | **Predicted (LS) mean diff.** | **95.00% CI of**  **diff.** | **Individual**  **P Value** |
| --- | --- | --- | --- |
| WT :Pre vs. WT :Vehicle | -0.2602 | -0.4744 to -0.04607 | 0.0061 |
| WT :Pre vs. WT :SNAP | -0.07270 | -0.3305 to 0.1851 | >0.9999 |
| WT :Pre vs. KO:Pre | -0.1093 | -0.2858 to 0.06728 | 0.9932 |
| WT :Pre vs. KO:Vehicle | -0.2467 | -0.4815 to -0.01198 | 0.0311 |
| WT :Pre vs. KO:SNAP | -0.05637 | -0.3888 to 0.2761 | >0.9999 |
| WT :Vehicle vs. WT :SNAP | 0.1875 | -0.1044 to 0.4795 | 0.8508 |
| WT :Vehicle vs. KO:Pre | 0.1510 | -0.07257 to 0.3745 | 0.6795 |
| WT :Vehicle vs. KO:Vehicle | 0.01352 | -0.2583 to 0.2854 | >0.9999 |
| WT :Vehicle vs. KO:SNAP | 0.2039 | -0.1558 to 0.5635 | >0.9999 |
| WT :SNAP vs. KO:Pre | -0.03657 | -0.3022 to 0.2290 | >0.9999 |
| WT :SNAP vs. KO:Vehicle | -0.1740 | -0.4814 to 0.1334 | >0.9999 |
| WT :SNAP vs. KO:SNAP | 0.01634 | -0.3708 to 0.4035 | >0.9999 |
| KO:Pre vs. KO:Vehicle | -0.1375 | -0.3808 to 0.1059 | >0.9999 |
| KO:Pre vs. KO:SNAP | 0.05290 | -0.2857 to 0.3915 | >0.9999 |
| KO:Vehicle vs. KO:SNAP | 0.1904 | -0.1819 to 0.5626 | >0.9999 |

# Table S7

Statistics for Figure S7A-S7L

| **Frequency** | **Factor** | **ANOVA results** | **p-value** |
| --- | --- | --- | --- |
| **AuC** | | | |
| P1 amplitude | Genotype | "F (1, 20) = 0.03025" | 0.8637 |
|  | Treatment | "F (1, 17) = 0.6828" | 0.4201 |
|  | Genotype x Treatment | "F (1, 17) = 0.2966" | 0.5931 |
| P1 latency | Genotype | "F (1, 20) = 0.2562" | 0.6183 |
|  | Treatment | "F (1, 17) = 0.6882" | 0.4183 |
|  | Genotype x Treatment | "F (1, 17) = 1.163" | 0.2959 |
| N1 amplitude | Genotype | "F (1, 20) = 0.06962" | 0.7946 |
|  | Treatment | "F (1, 17) = 0.3064" | 0.5871 |
|  | Genotype x Treatment | "F (1, 17) = 0.01605" | 0.9007 |
| N1 latency | Genotype | "F (1, 20) = 0.2261" | 0.6396 |
|  | Treatment | "F (1, 17) = 0.003827" | 0.9514 |
|  | Genotype x Treatment | "F (1, 17) = 0.08865" | 0.7695 |
| P2 amplitude | Genotype | "F (1, 20) = 0.2682" | 0.6102 |
|  | Treatment | "F (1, 17) = 0.004136" | 0.9495 |
|  | Genotype x Treatment | "F (1, 17) = 0.01481" | 0.9046 |
| P2 latency | Genotype | "F (1, 20) = 0.7012" | 0.4123 |
|  | Treatment | "F (1, 17) = 5.268" | 0.0347 |
|  | Genotype x Treatment | "F (1, 17) = 0.002633" | 0.9597 |
| **FC** | | | |
| P1 amplitude | Genotype | "F (1, 20) = 1.378" | 0.2543 |
|  | Treatment | "F (1, 17) = 1.895" | 0.1865 |
|  | Genotype x Treatment | "F (1, 17) = 1.445" | 0.2457 |
| P1 latency | Genotype | "F (1, 36) = 0.009201" | 0.9241 |
|  | Treatment | "F (1, 36) = 4.259" | 0.0463 |
|  | Genotype x Treatment | "F (1, 36) = 0.1509" | 0.6999 |
| N1 amplitude | Genotype | "F (1, 20) = 0.04591" | 0.8325 |
|  | Treatment | "F (1, 17) = 0.3190" | 0.5796 |
|  | Genotype x Treatment | "F (1, 17) = 1.129" | 0.3028 |
| N1 latency | Genotype | "F (1, 20) = 0.01005" | 0.9211 |
|  | Treatment | "F (1, 17) = 0.005249" | 0.9431 |
|  | Genotype x Treatment | "F (1, 17) = 1.326" | 0.2654 |
| P2 amplitude | Genotype | "F (1, 20) = 0.2456" | 0.6256 |
|  | Treatment | "F (1, 17) = 0.6518" | 0.4306 |
|  | Genotype x Treatment | "F (1, 17) = 0.1529" | 0.7007 |
| P2 latency | Genotype | "F (1, 20) = 0.06216" | 0.8057 |
|  | Treatment | "F (1, 17) = 0.7778" | 0.3901 |
|  | Genotype x Treatment | "F (1, 17) = 0.1582" | 0.6958 |

# Table S8

Statistics for Figure 8B

Locomotor activity in whole arena untreated

| Locomotor activity | **Mean** | **SD** | **N** |
| --- | --- | --- | --- |
| Ctrl WT | 15.92 | 3.896 | 30 |
| cKO | 18.80 | 3.664 | 21 |
| Statistics | Two-tailed, unpaired t-test | t=2.665, df=49, p=0.0104  η²=0.1266 |  |

Statistics for Figure 8C

Locomotor activity in whole arena treated

| Locomotor activity | **Mean** | **SD** | **N** |
| --- | --- | --- | --- |
| cKO Veh | 14.19 | 2.098 | 13 |
| cKO SNAP | 16.08 | 8.800 | 12 |
| Statistics | Two-tailed, unpaired t-test | t=0.7532, df=23, p=0.4590  η²=0.02407 |  |

Statistics for Figure 8D

Locomotor activity in thigmotaxis untreated

| Locomotor activity | **Mean** | **SD** | **N** |
| --- | --- | --- | --- |
| Ctrl WT | 14.22 | 3.924 | 30 |
| cKO | 17.89 | 3.853 | 21 |
| Statistics | Two-tailed, unpaired t-test | t=3.311, df=49, p=0.0018  η²=0.1828 |  |

Statistics for Figure 8E

Locomotor activity in thigmotaxis treated

| Locomotor activity | **Mean** | **SD** | **N** |
| --- | --- | --- | --- |
| cKO Veh | 13.08 | 2.117 | 13 |
| cKO SNAP | 13.87 | 7.077 | 12 |
| Statistics | Two-tailed, unpaired t-test | t=0.3829, df=23, p=0.7053  η²=0.006332 |  |

Statistics for Figure 8F

Exploratory behavior - untreated

| **ANOVA table** | **SS (Type III)** | **DF** | **MS** | **F (DFn, DFd)** | **P value** | **% of total variation** |
| --- | --- | --- | --- | --- | --- | --- |
| Preference x Genotype | 315.0 | 1 | 315.0 | F (1, 142) = 2.112 | P=0.1483 | 1.363 |
| Preference | 1818 | 1 | 1818 | F (1, 142) = 12.19 | P=0.0006 | 7.868 |
| Genotype | 1.463 | 1 | 1.463 | F (1, 142) = 0.009810 | P=0.9212 | 0.006333 |

| **Šídák's multiple comparisons test** | **Predicted (LS) mean diff.** | **95.00% CI of**  **diff.** | **Individual**  **P Value** |
| --- | --- | --- | --- |
| Thigmotaxis |  |  |  |
| cKO vs. Ctrl WT | -2.782 | -9.348 to 3.784 | 0.5642 |
| Open Field |  |  |  |
| cKO vs. Ctrl WT | 3.189 | -3.377 to 9.755 | 0.4732 |
| Ctrl WT |  |  |  |
| Open Field vs. Thigmotaxis | -10.16 | -17.28 to -3.030 | 0.0032 |
| cKO |  |  |  |
| Open Field vs. Thigmotaxis | -4.186 | -10.14 to 1.767 | 0.2153 |

Statistics for Figure 8G

Exploratory behavior - treated

| **ANOVA table** | **SS (Type III)** | **DF** | **MS** | **F (DFn, DFd)** | **P value** | **% of total variation** |
| --- | --- | --- | --- | --- | --- | --- |
| Preference x Genotype | 4875 | 1 | 4875 | F (1, 46) = 28.56 | P<0.0001 | 27.56 |
| Preference | 5207 | 1 | 5207 | F (1, 46) = 30.50 | P<0.0001 | 29.44 |
| Genotype | 141.8 | 1 | 141.8 | F (1, 46) = 0.8306 | P=0.3668 | 0.8016 |

| **Šídák's multiple comparisons test** | **Predicted (LS) mean diff.** | **95.00% CI of**  **diff.** | **Individual**  **P Value** |
| --- | --- | --- | --- |
| Thigmotaxis |  |  |  |
| cKO SNAP vs. cKO Vehicle | 16.39 | 4.302 to 28.49 | 0.0060 |
| Open Field |  |  |  |
| cKO SNAP vs. cKO Vehicle | -23.14 | -35.23 to -11.04 | 0.0001 |
| cKO Vehicle |  |  |  |
| Open Field vs. Thigmotaxis | -0.6615 | -12.51 to 11.19 | 0.9896 |
| cKO SNAP |  |  |  |
| Open Field vs. Thigmotaxis | -40.19 | -52.52 to -27.86 | <0.0001 |

# Western Blots

FMRP blot for Figure 1D


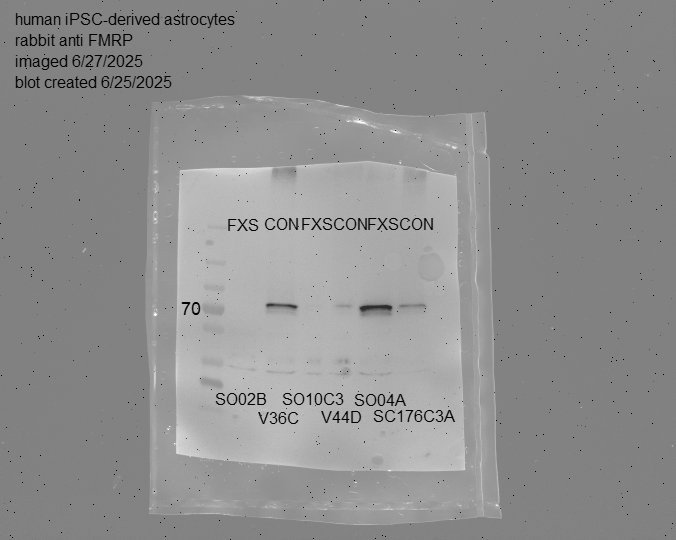


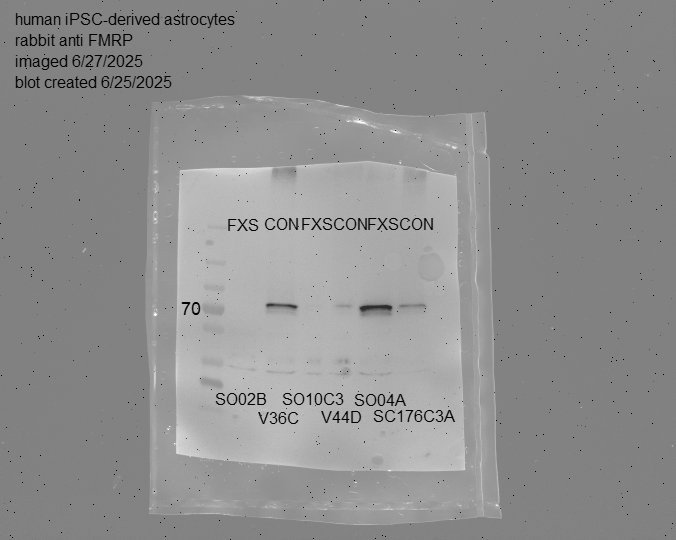


Beta-actin blot for Figure 1D, 1I


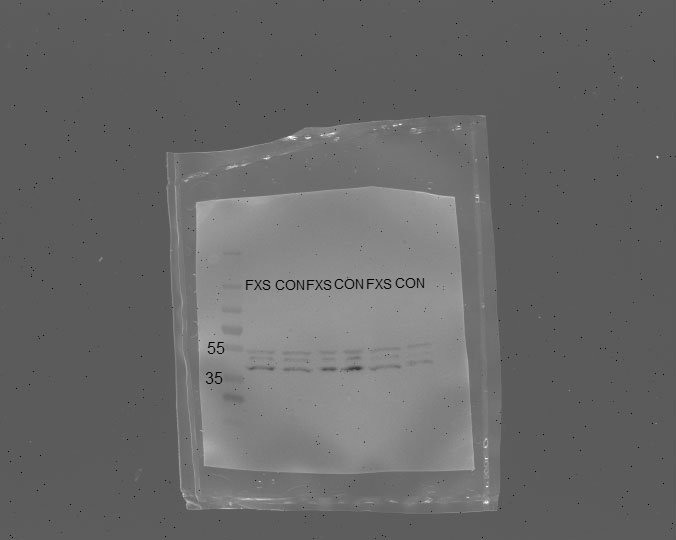


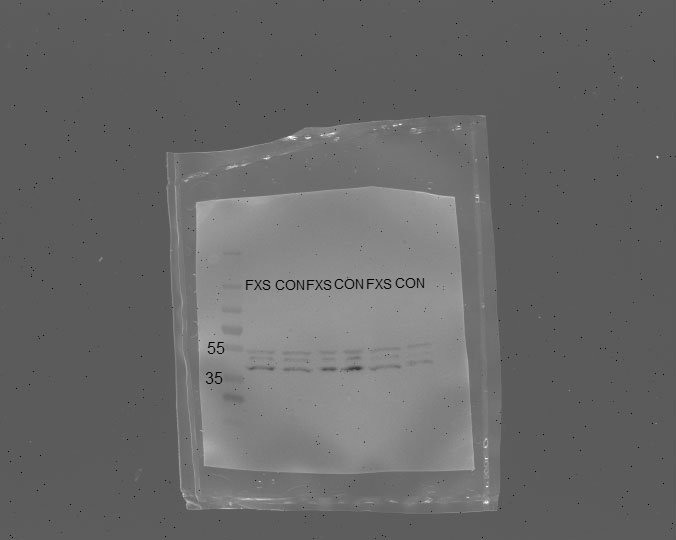


GAD65/67 blots for Figure 1I


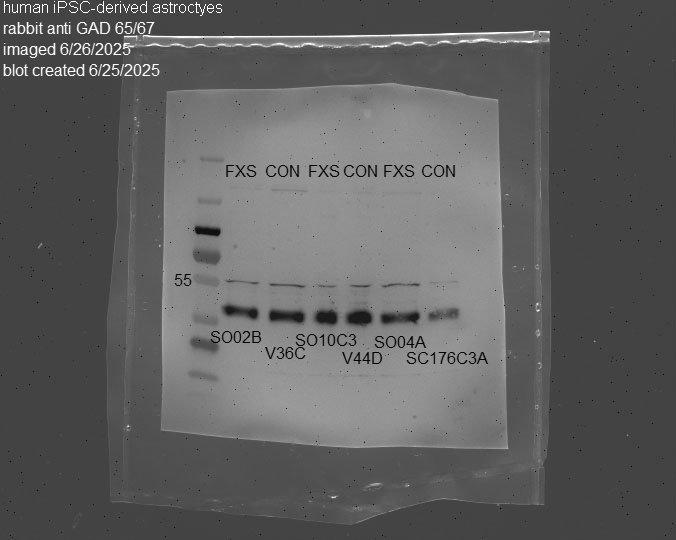


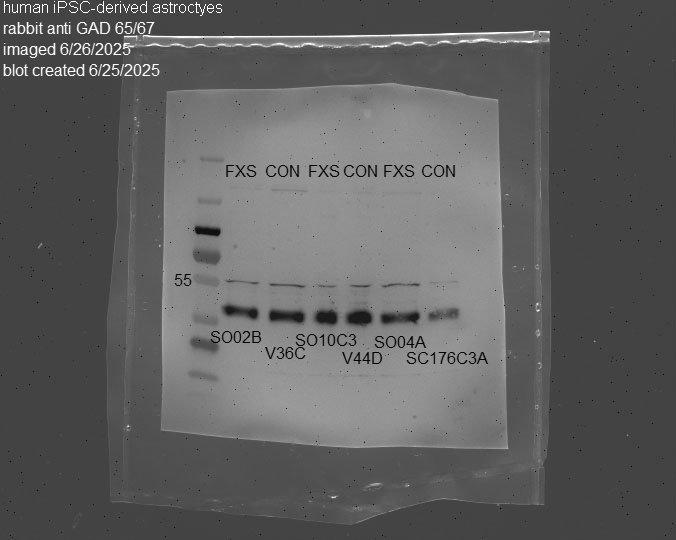


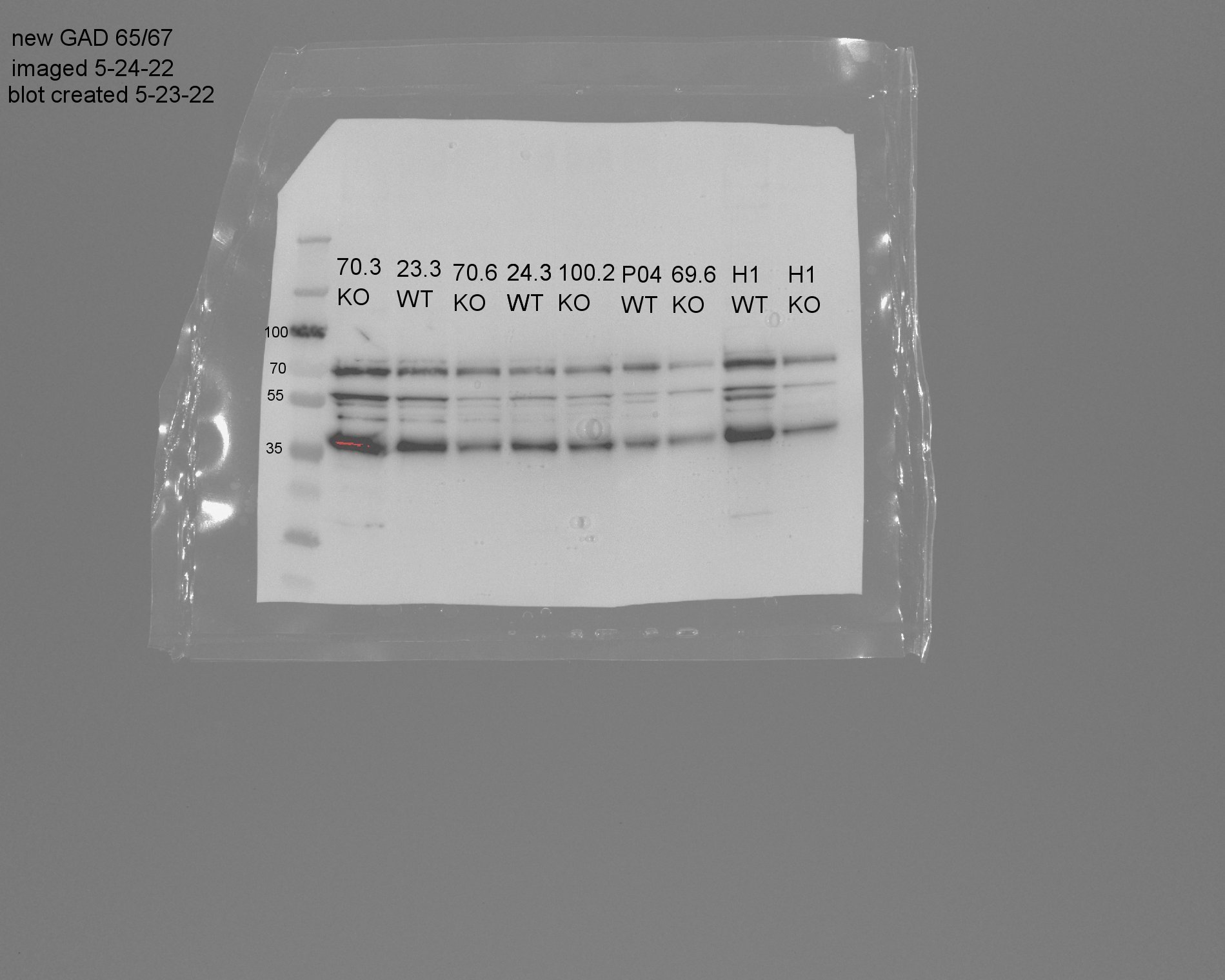


Beta-actin blot for Figure 1I


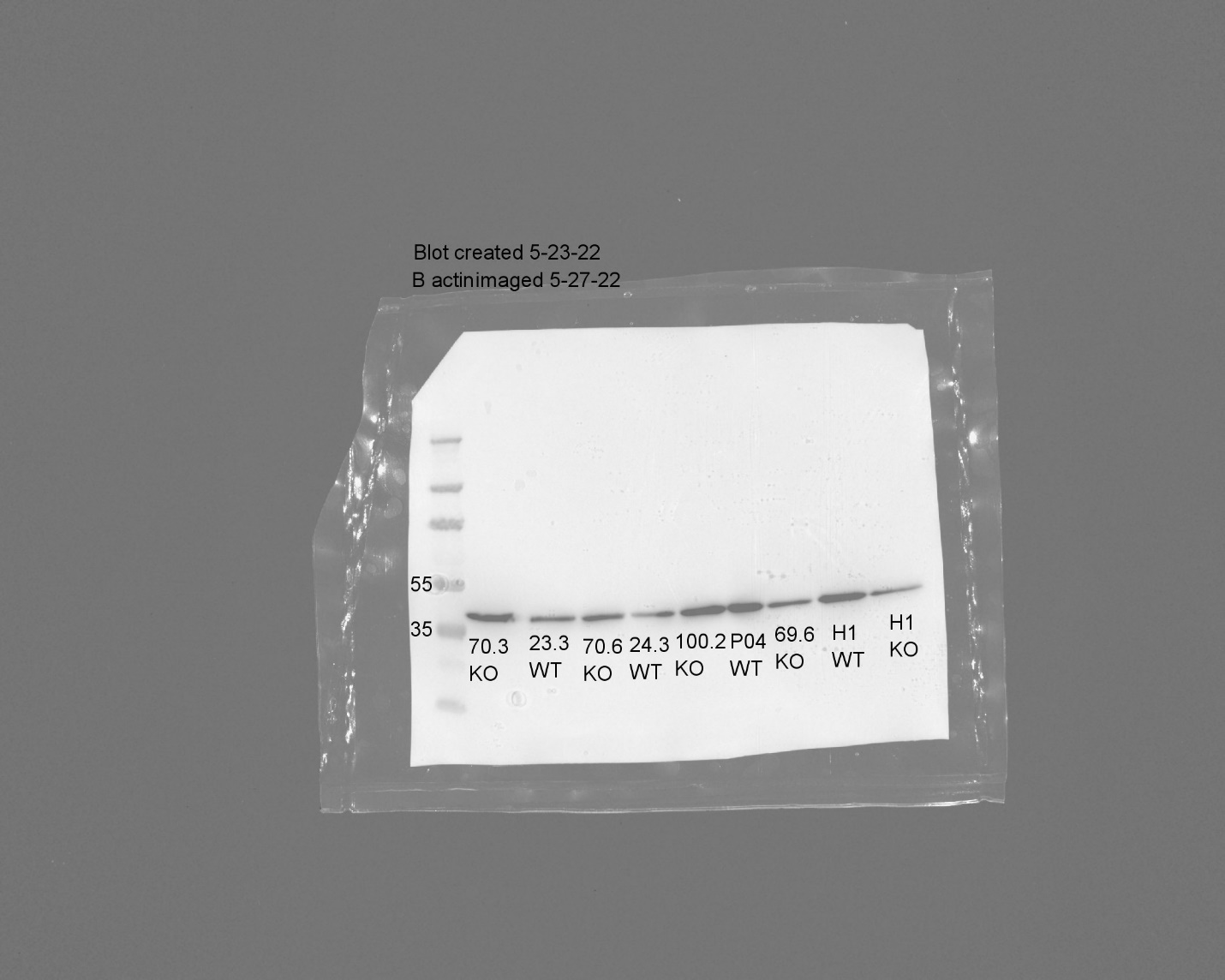


GAD 65/67 blots for Figure 2B
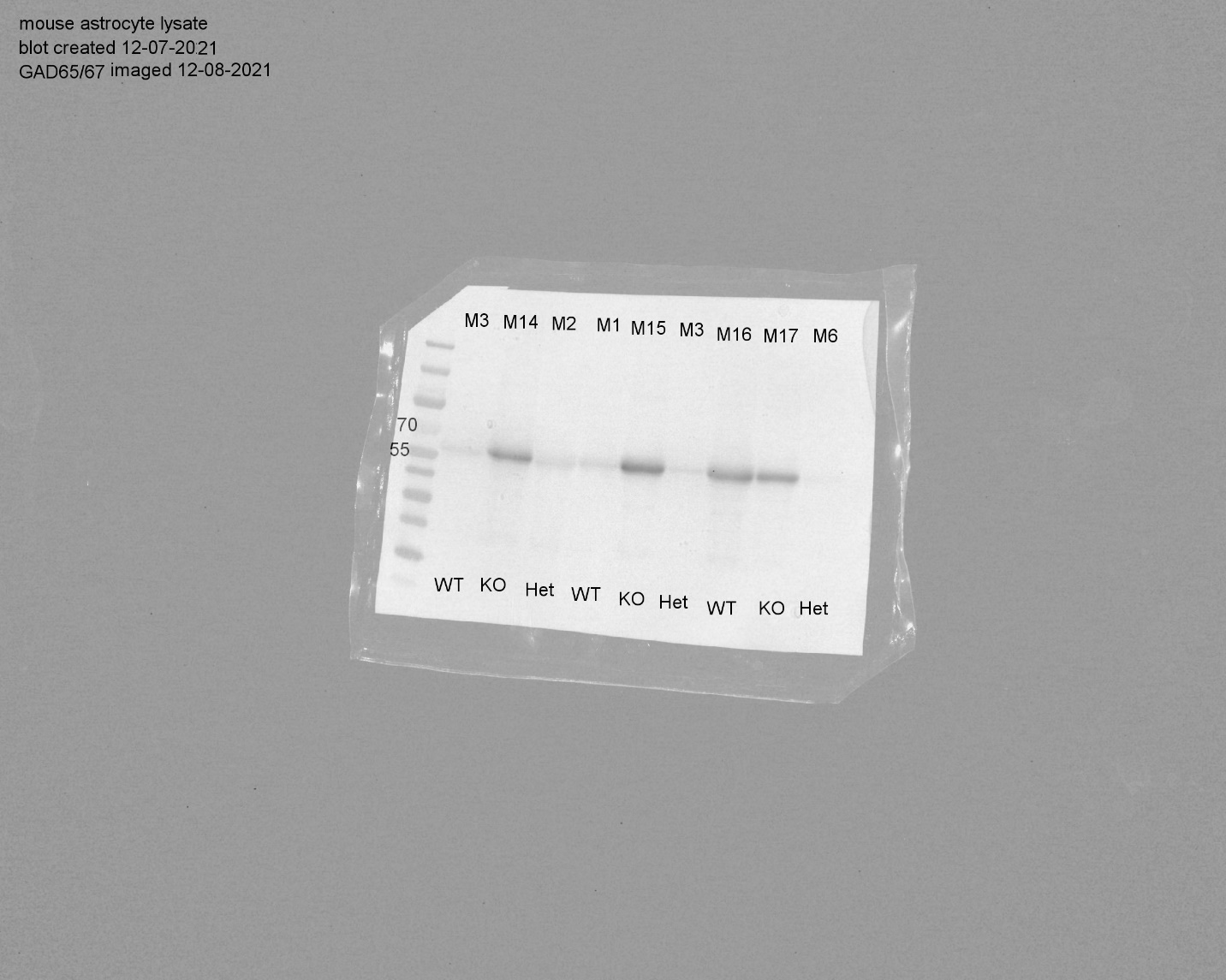

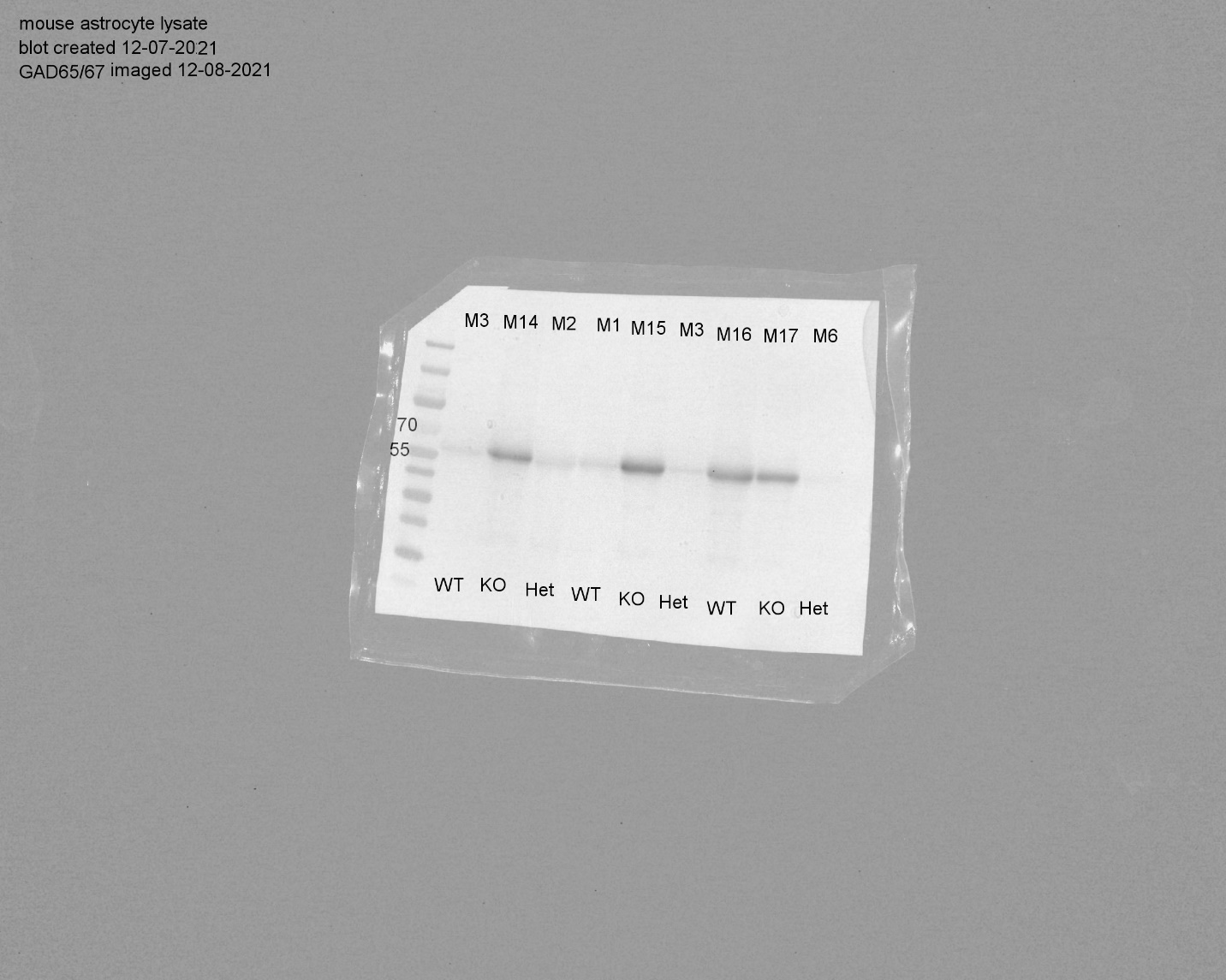


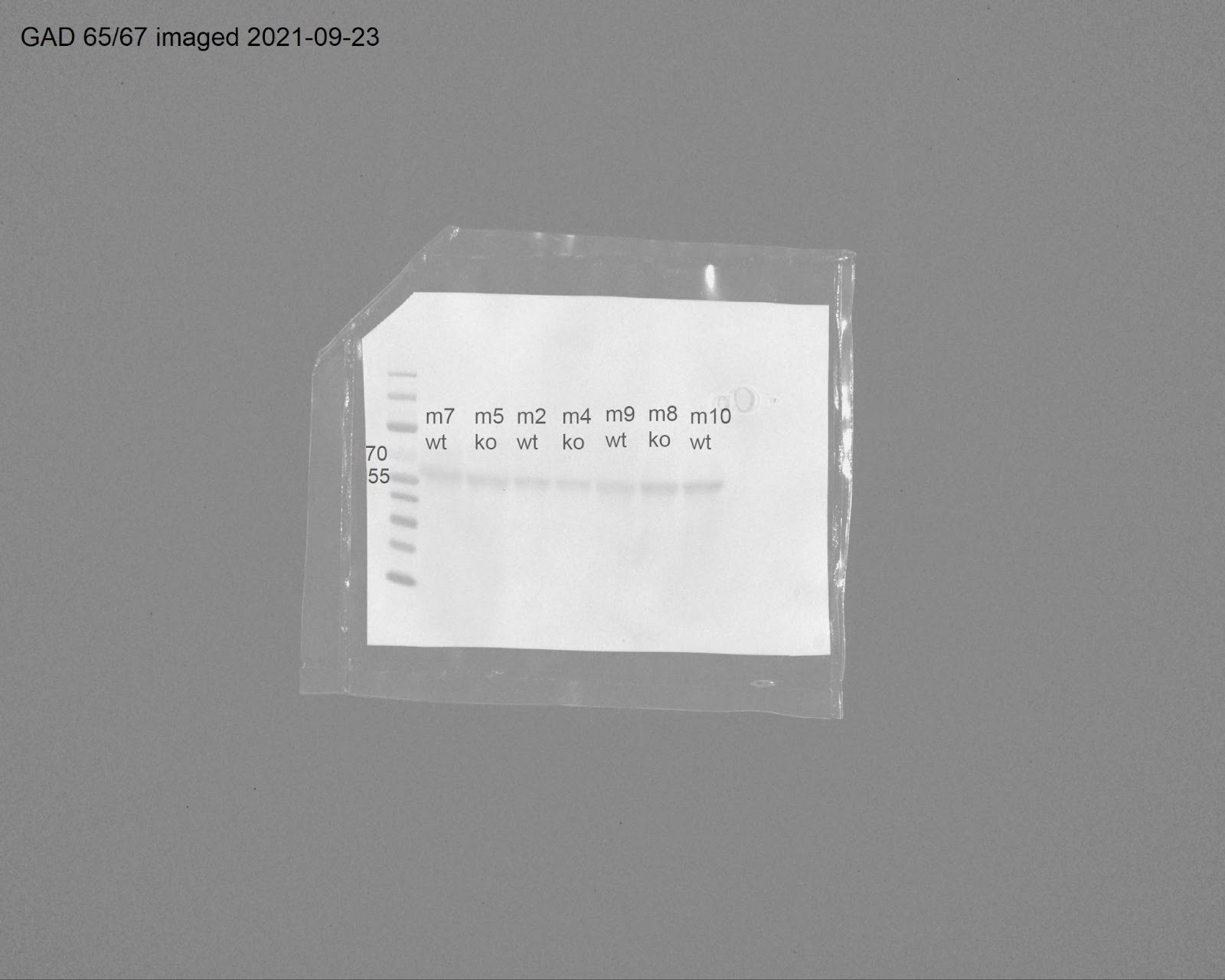


Beta-actin blots for Figure 2B


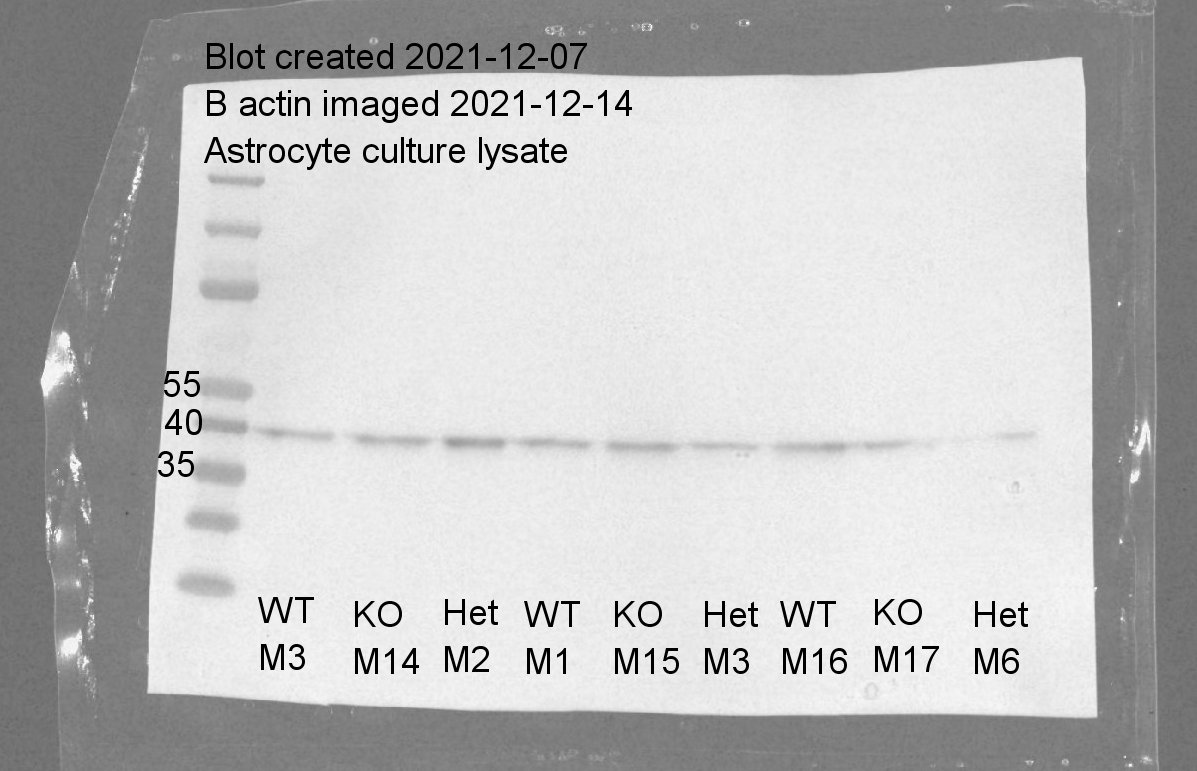


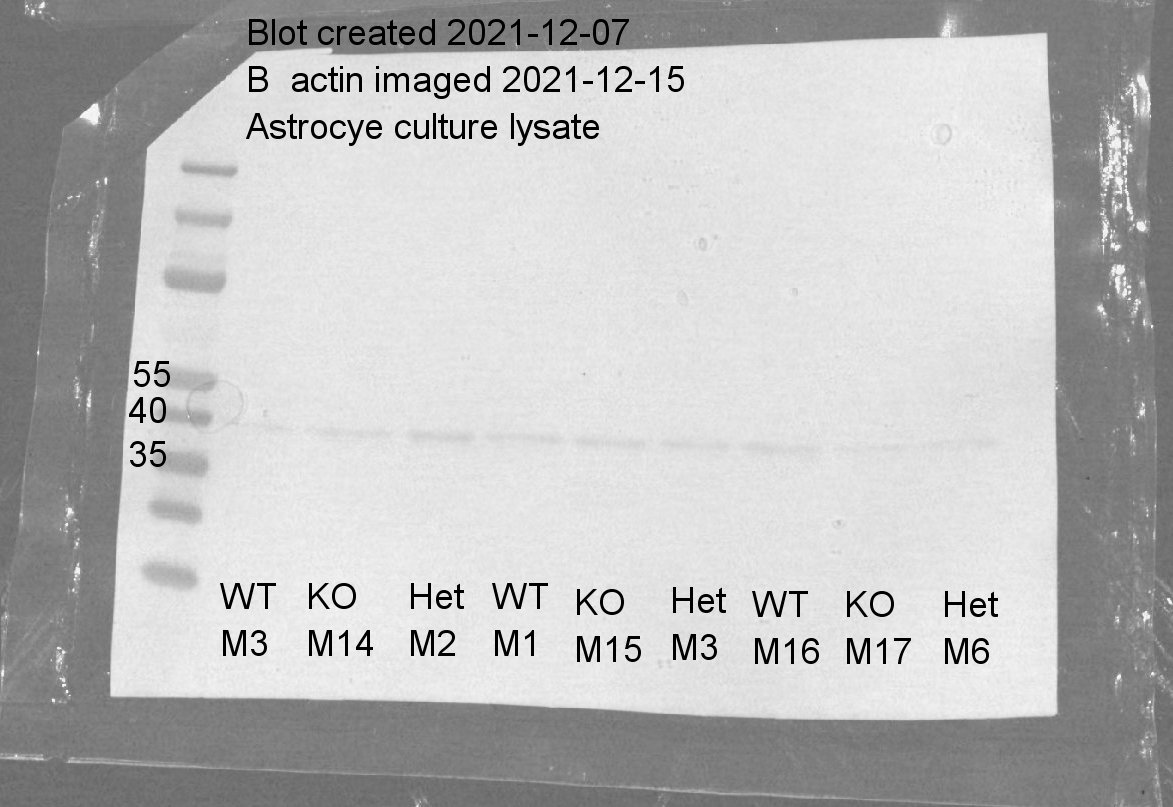

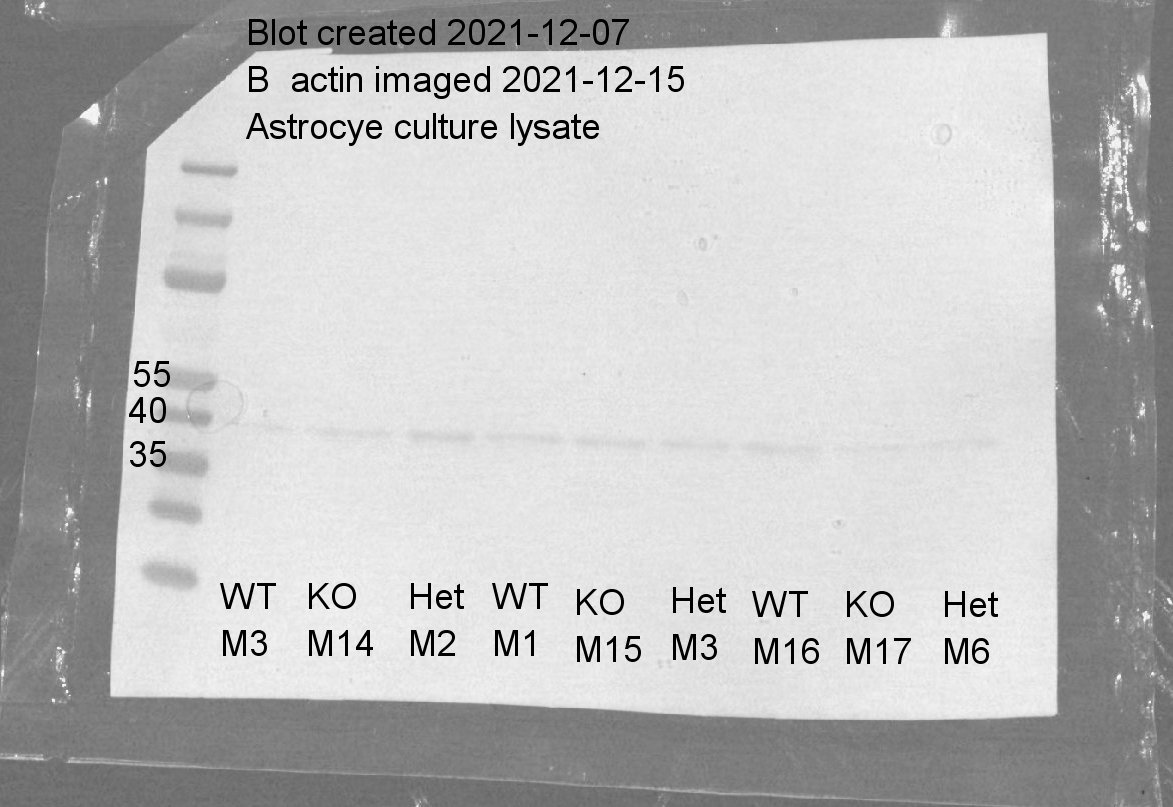


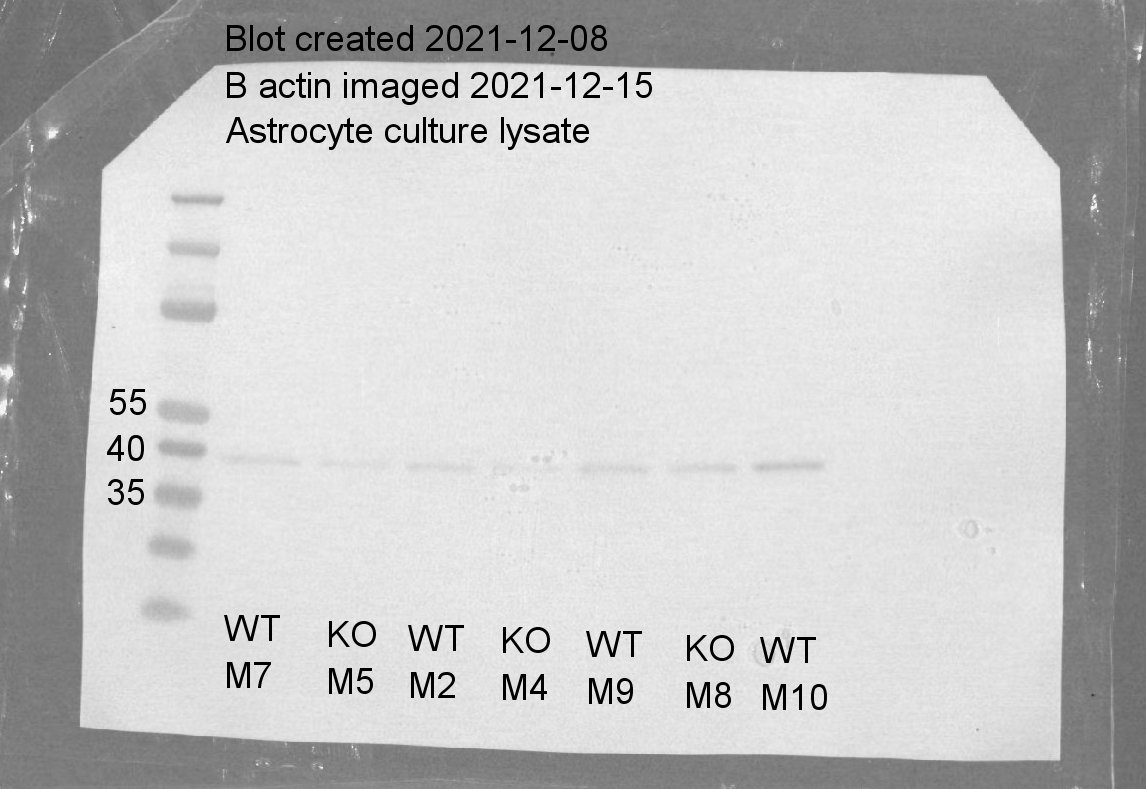

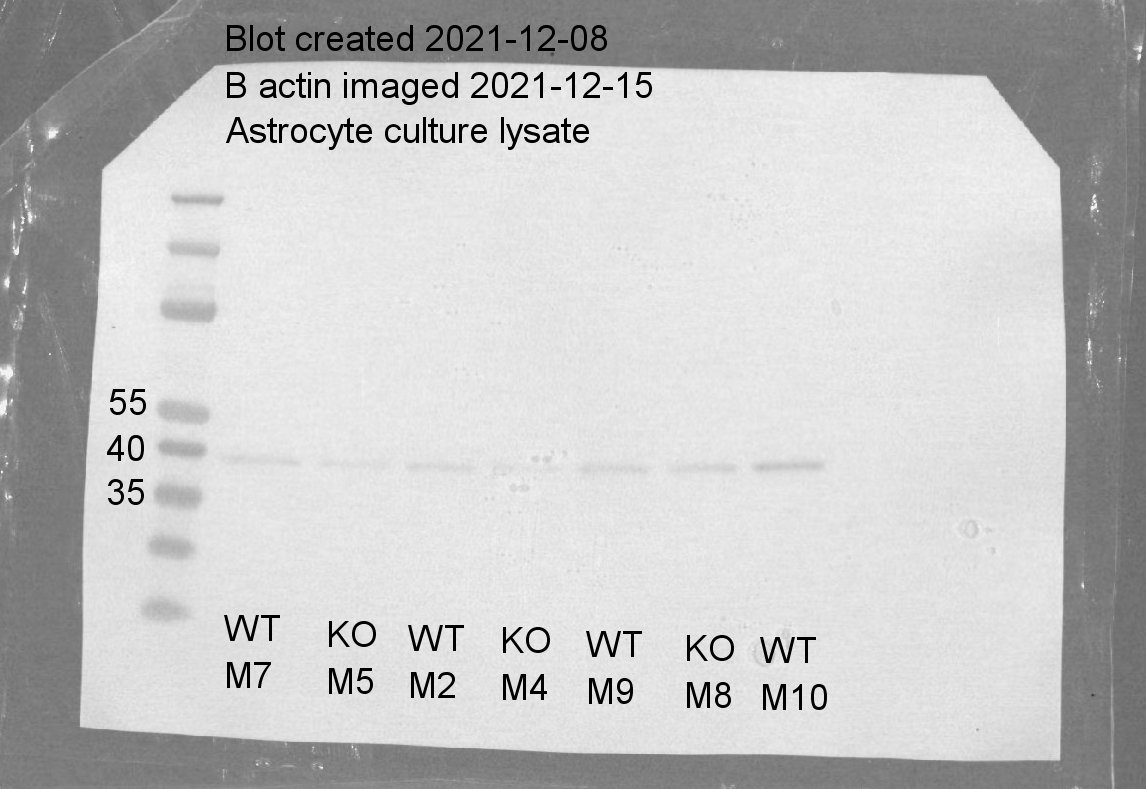


Aldha1 blots for Figure 2B


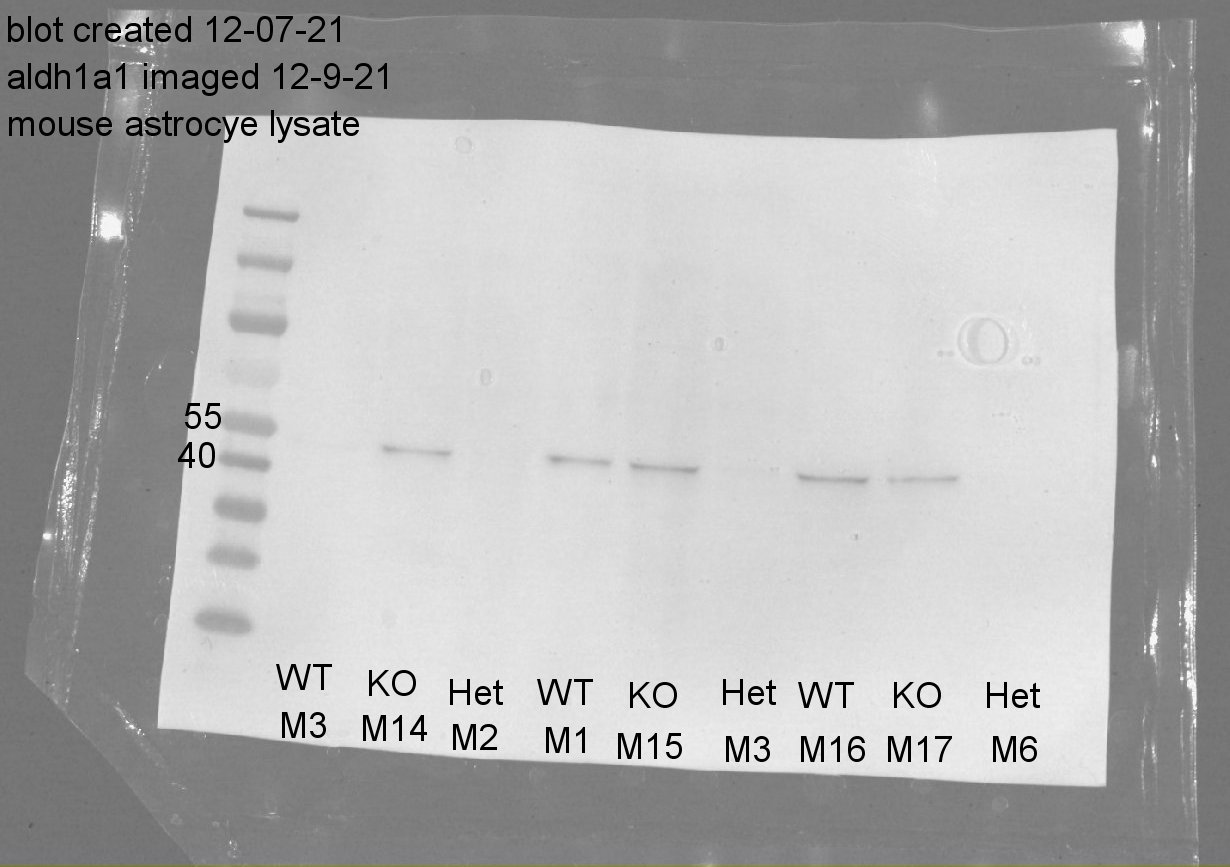


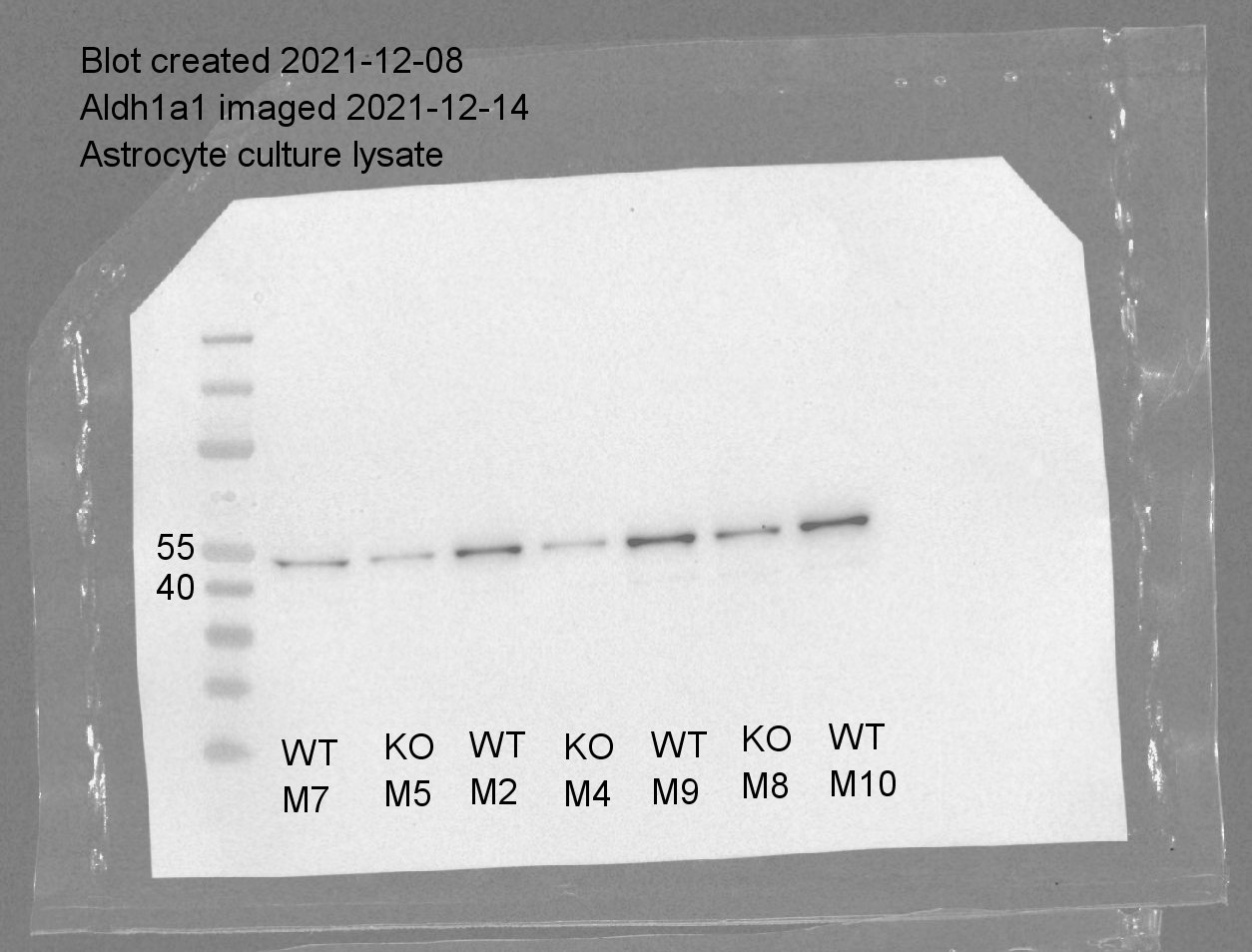


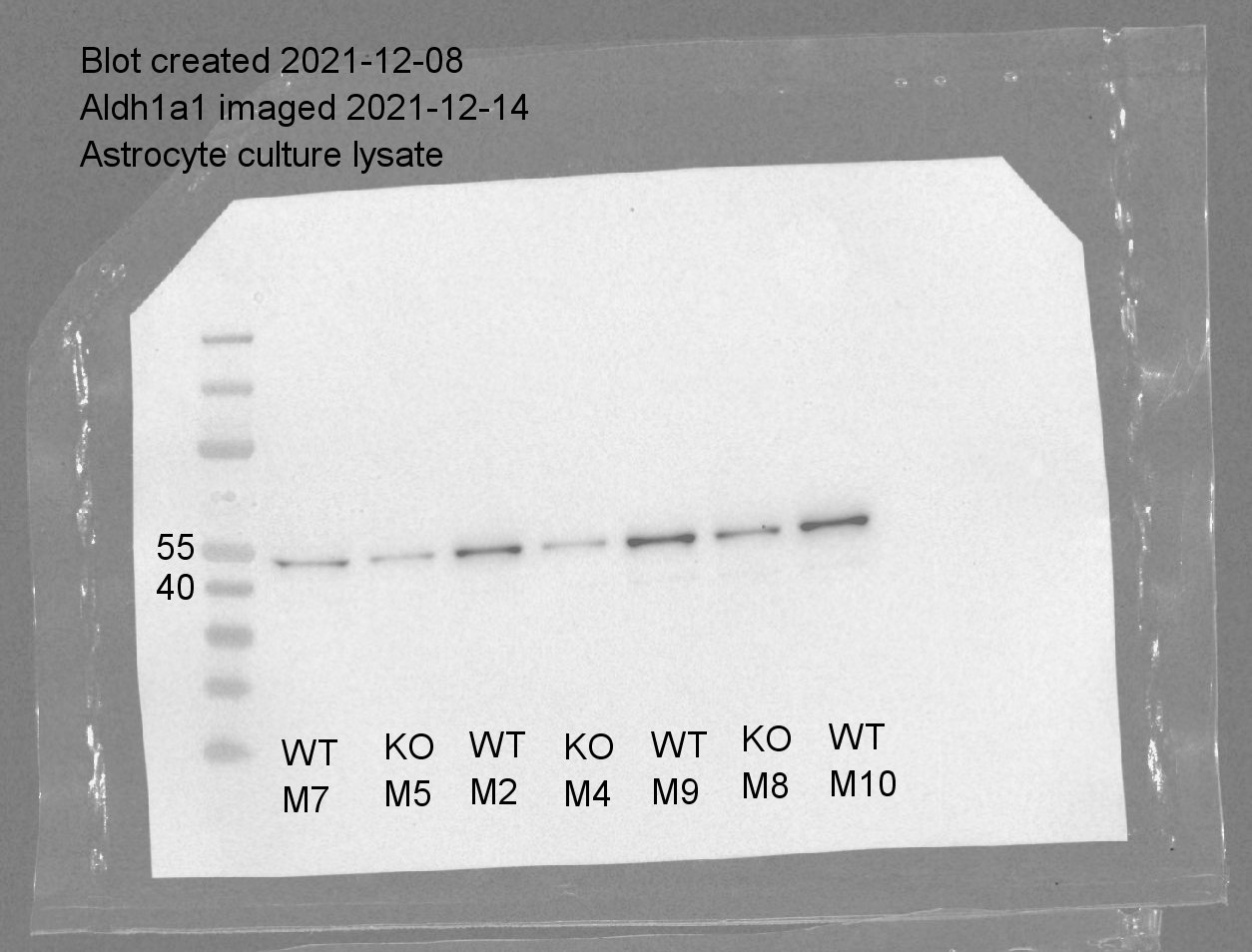


GAT3 blots for Figure 3G
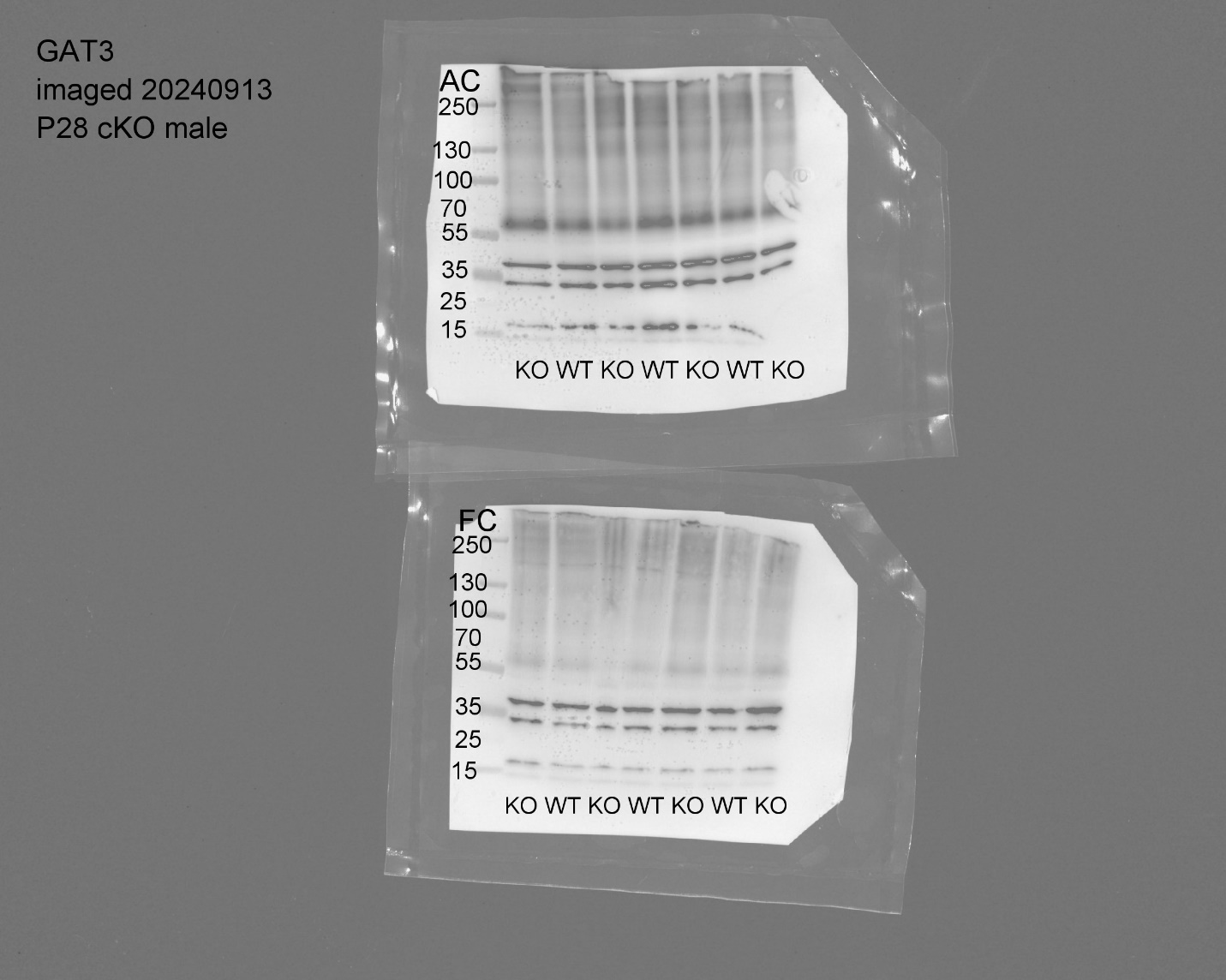

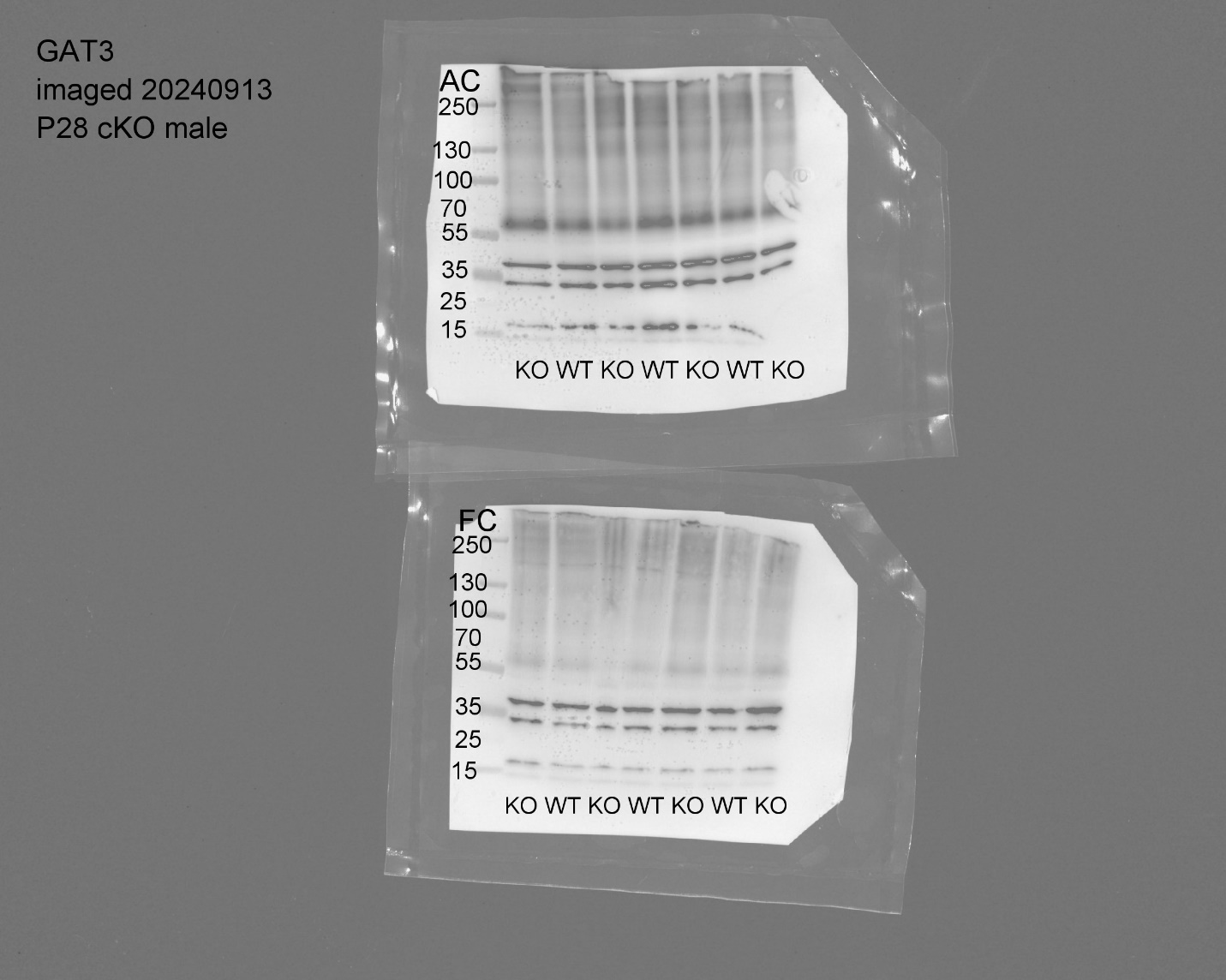


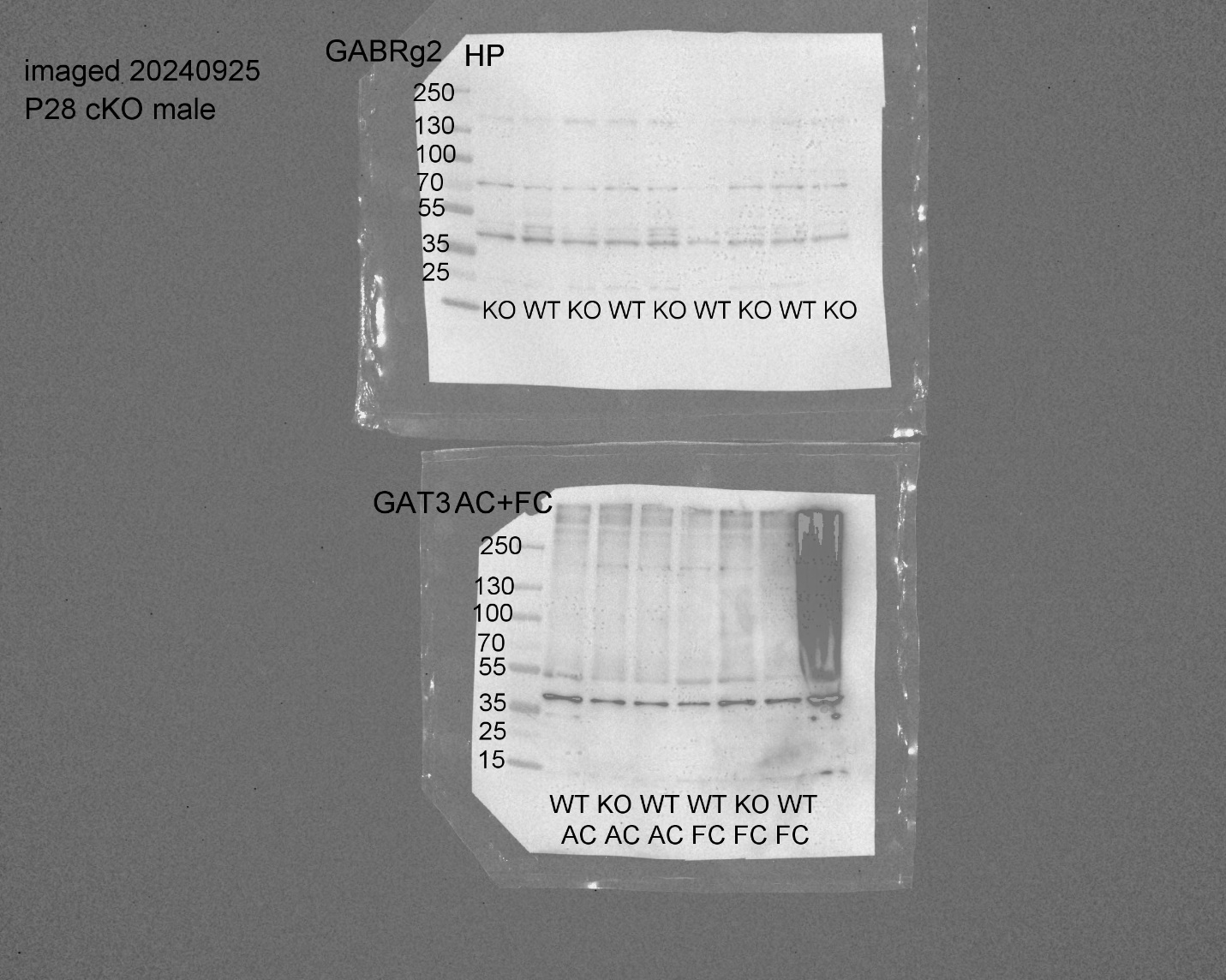

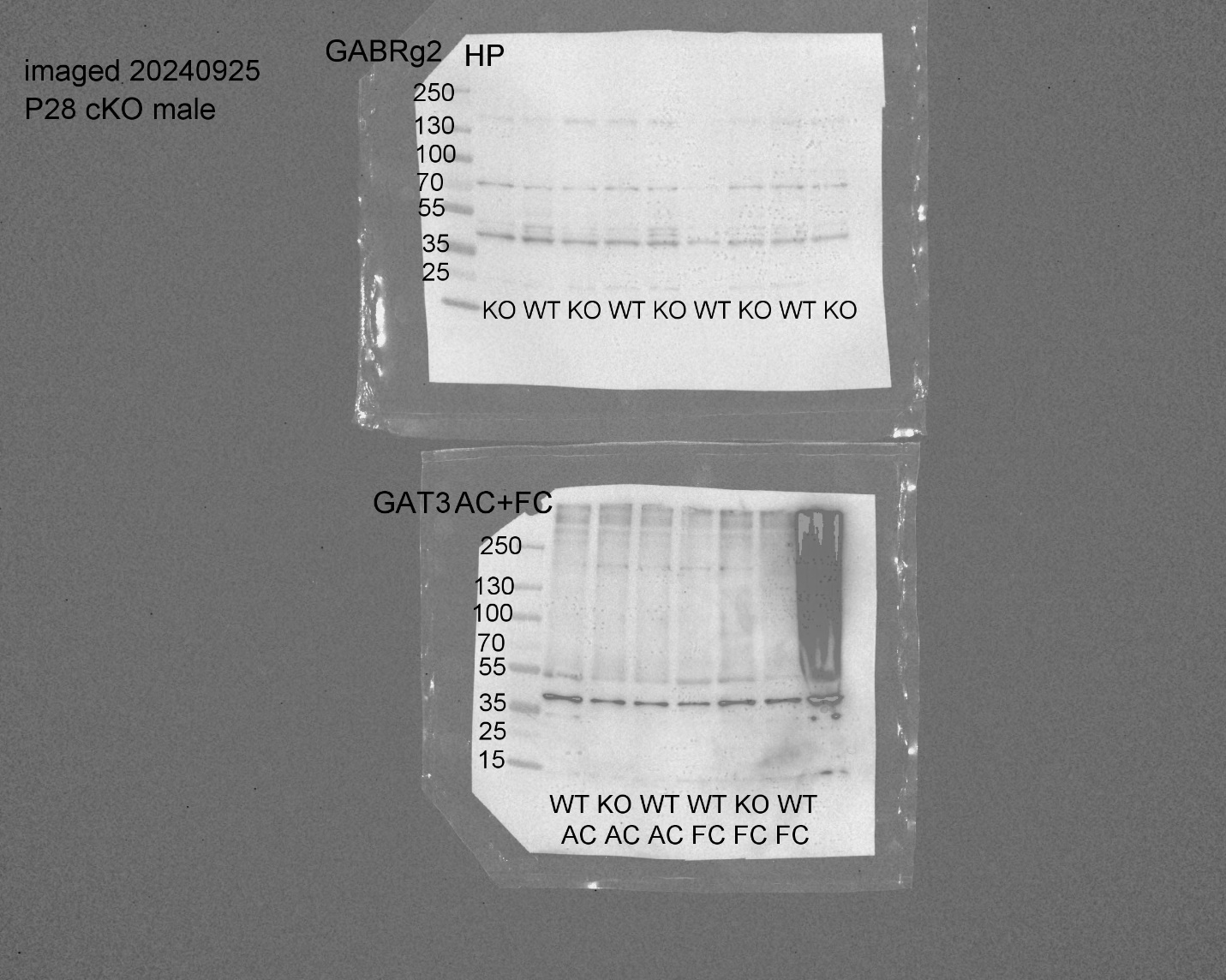


Beta-actin blot for Figure 3G, S2E
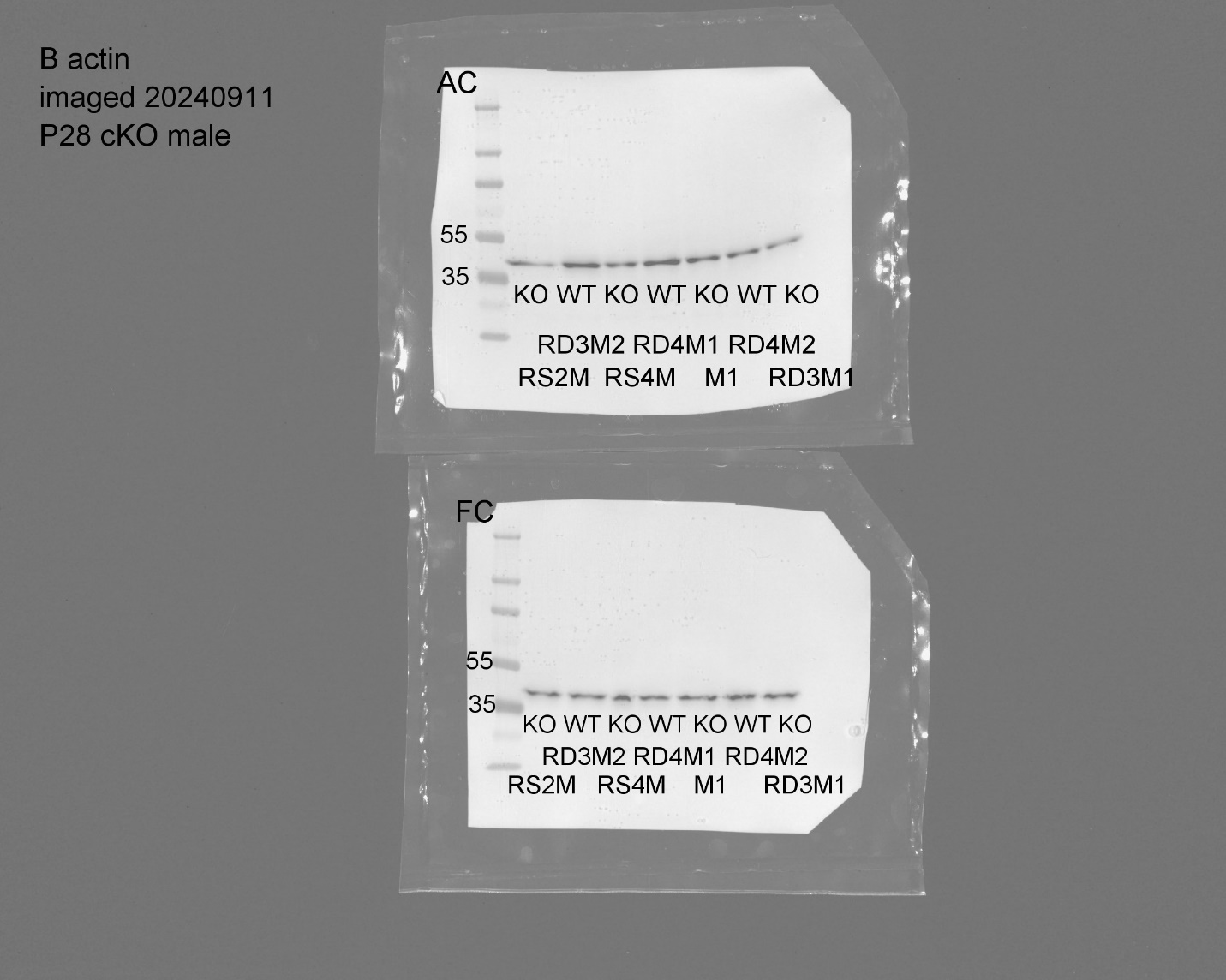

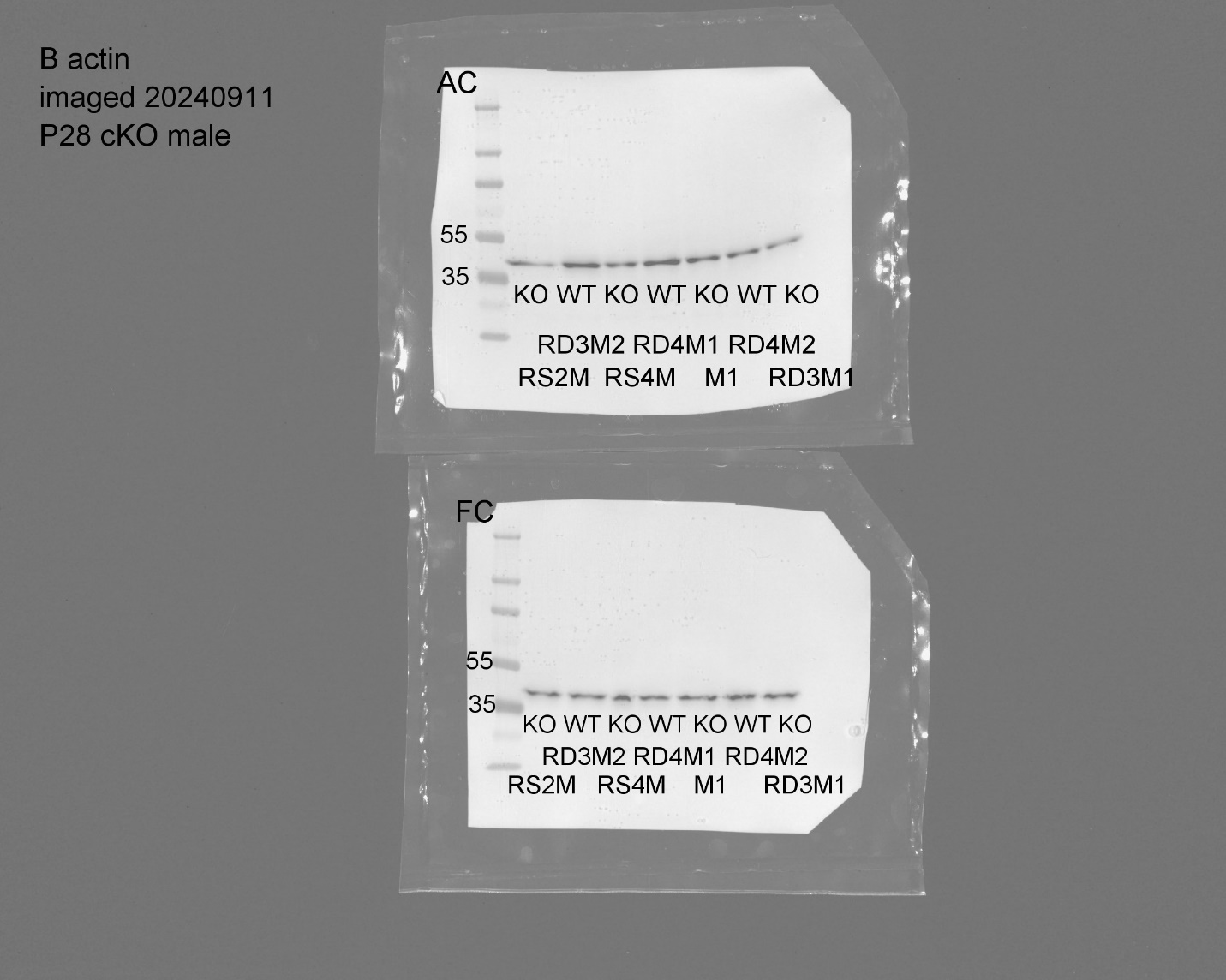


Beta-actin blot for Figure 3G


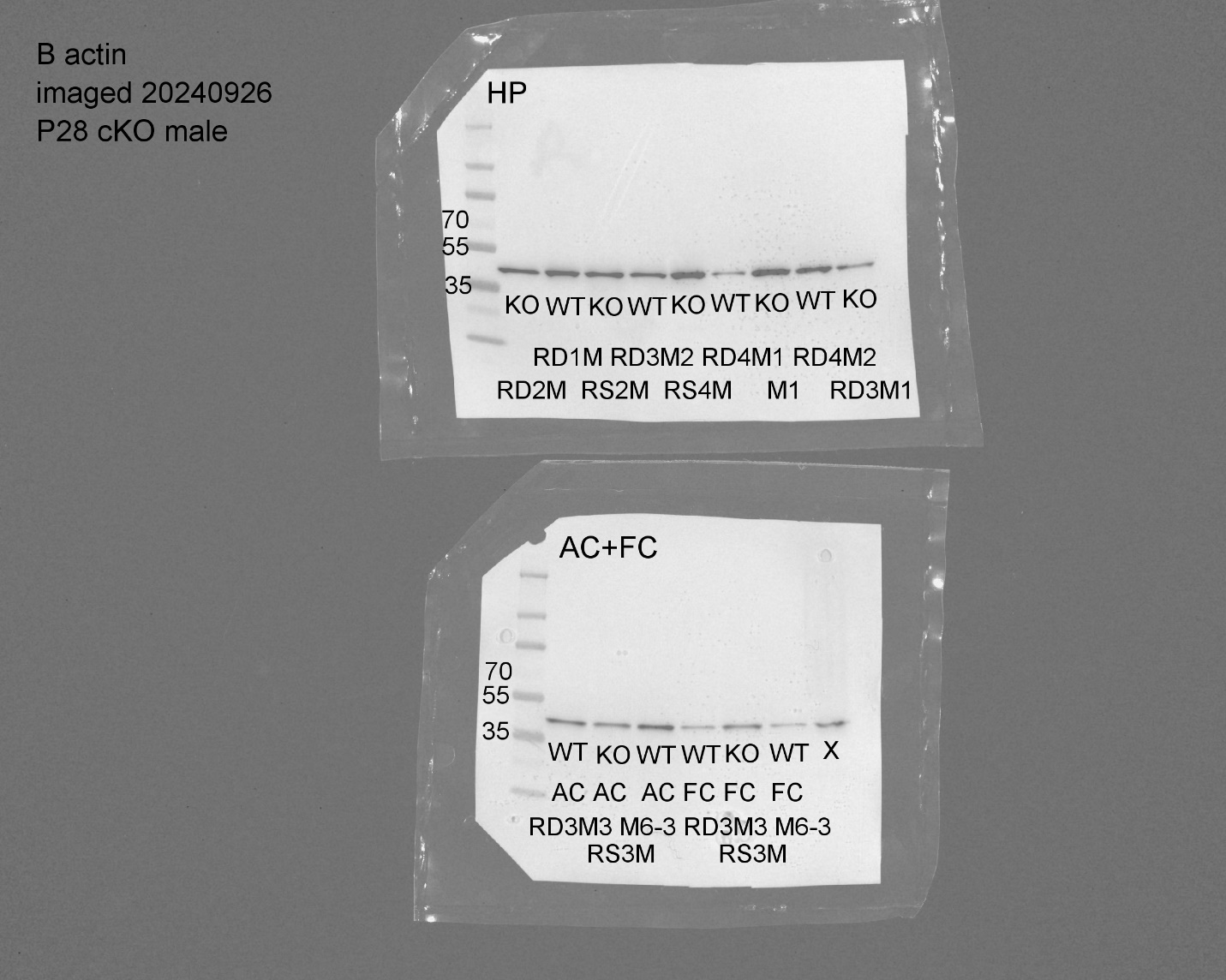

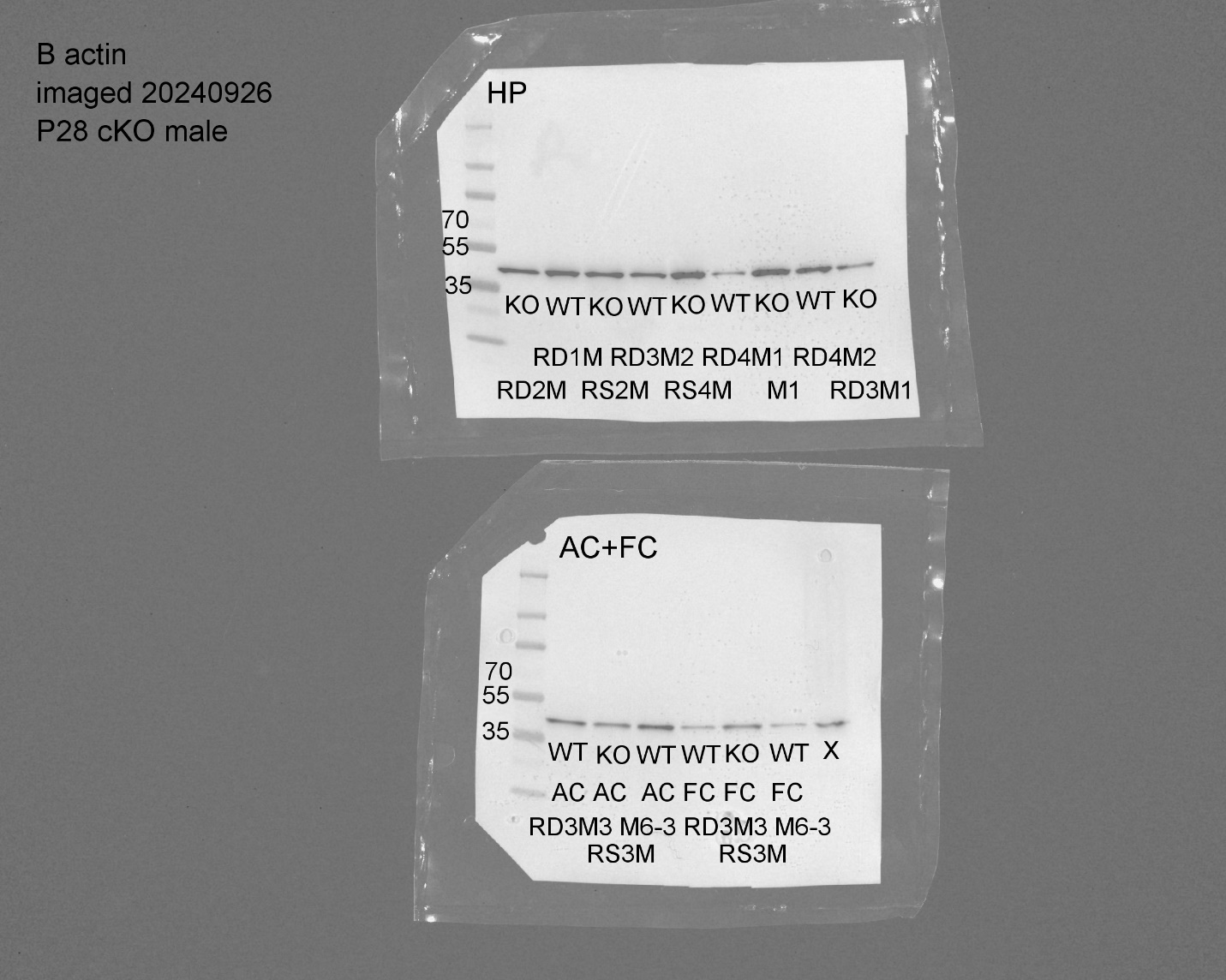


GABRg2 blots for Figure S2E
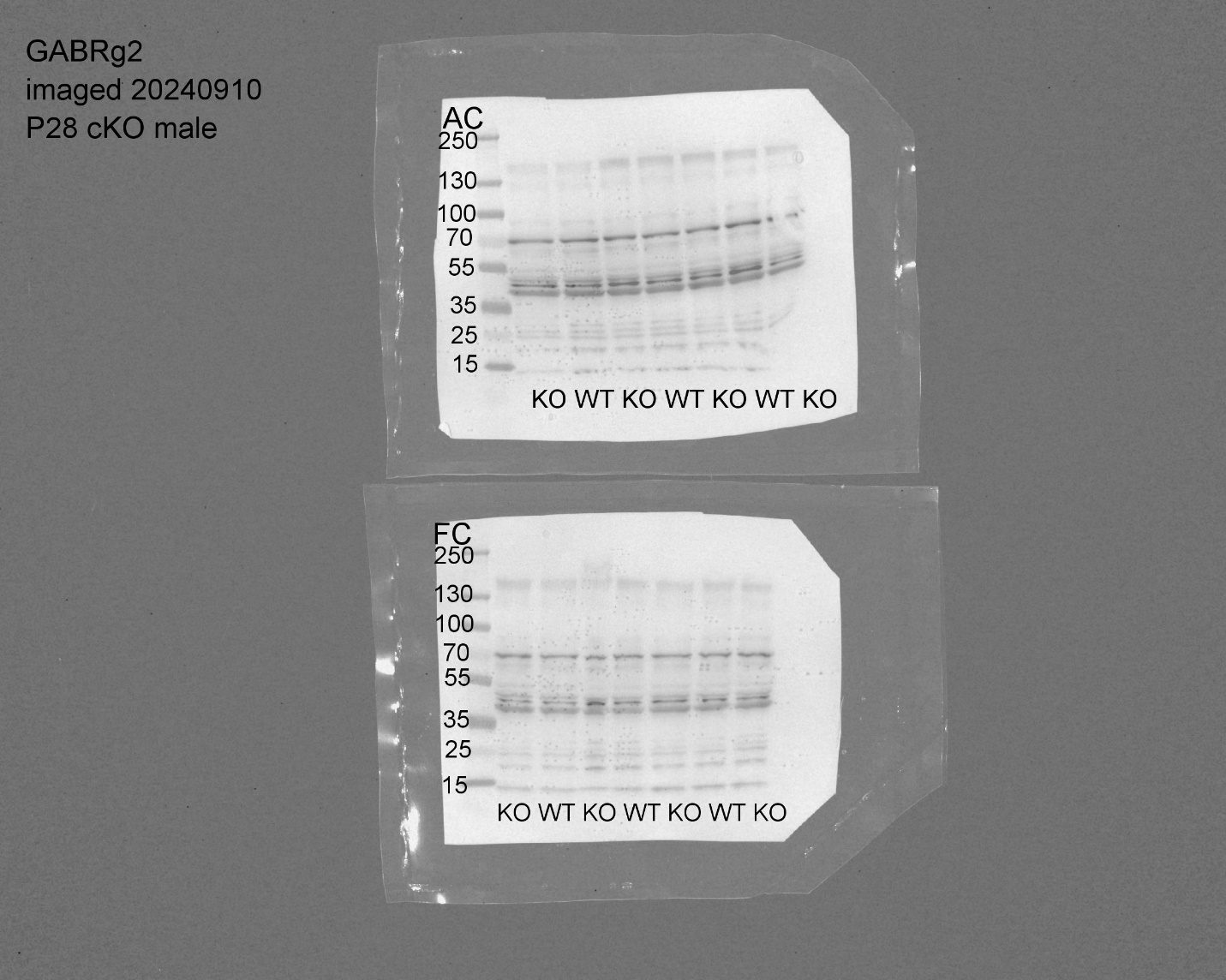

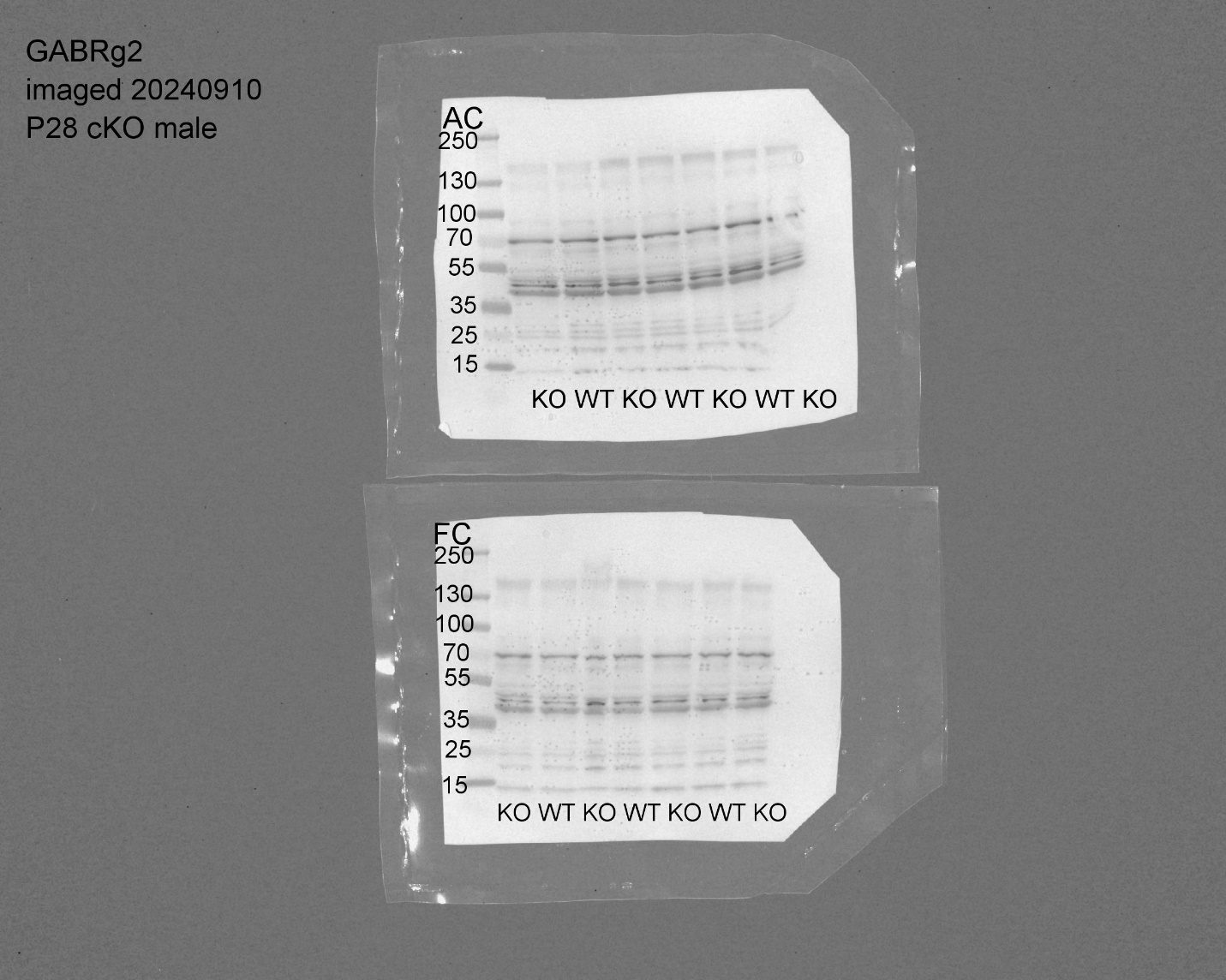


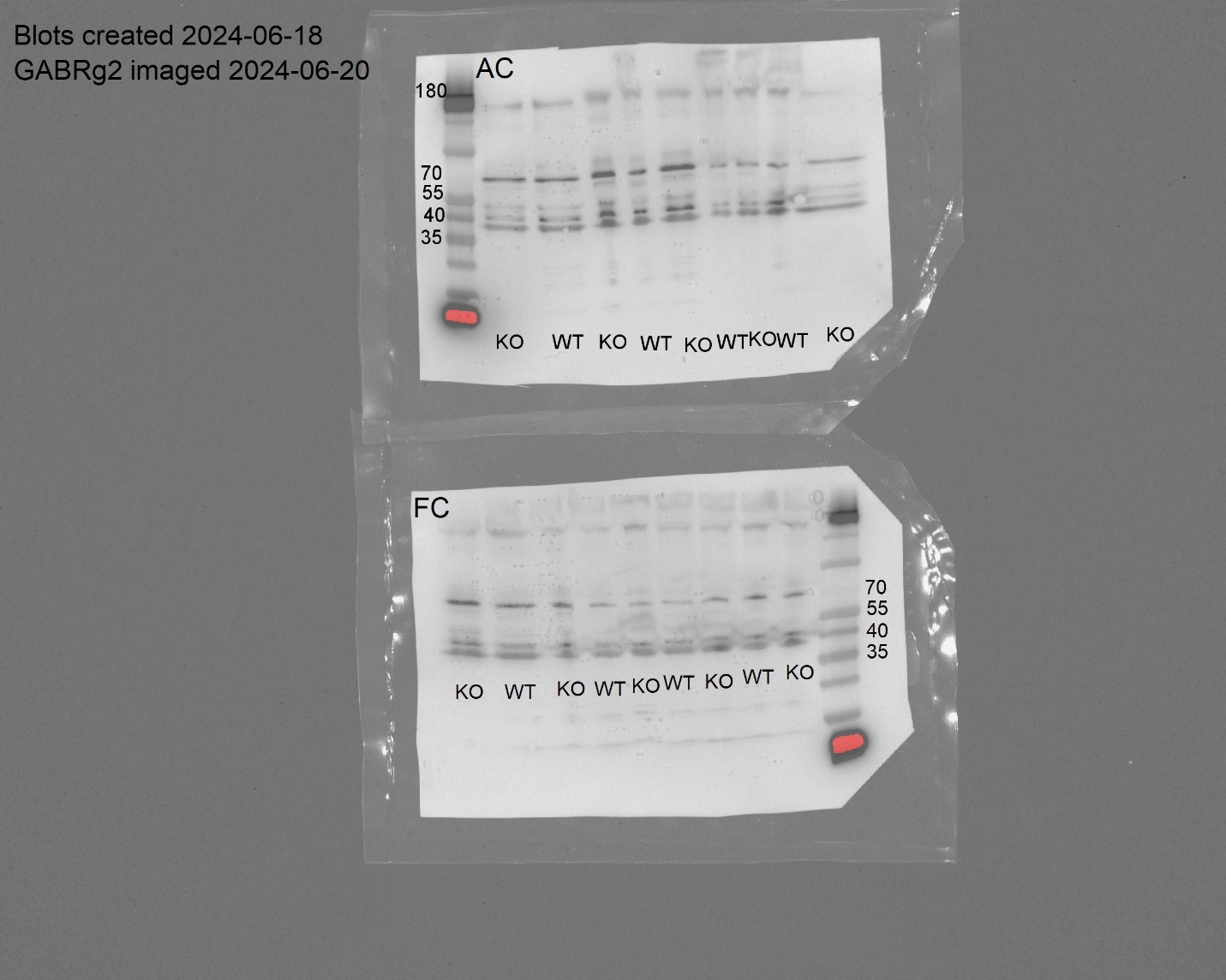


Beta-actin blot for Figure S2E


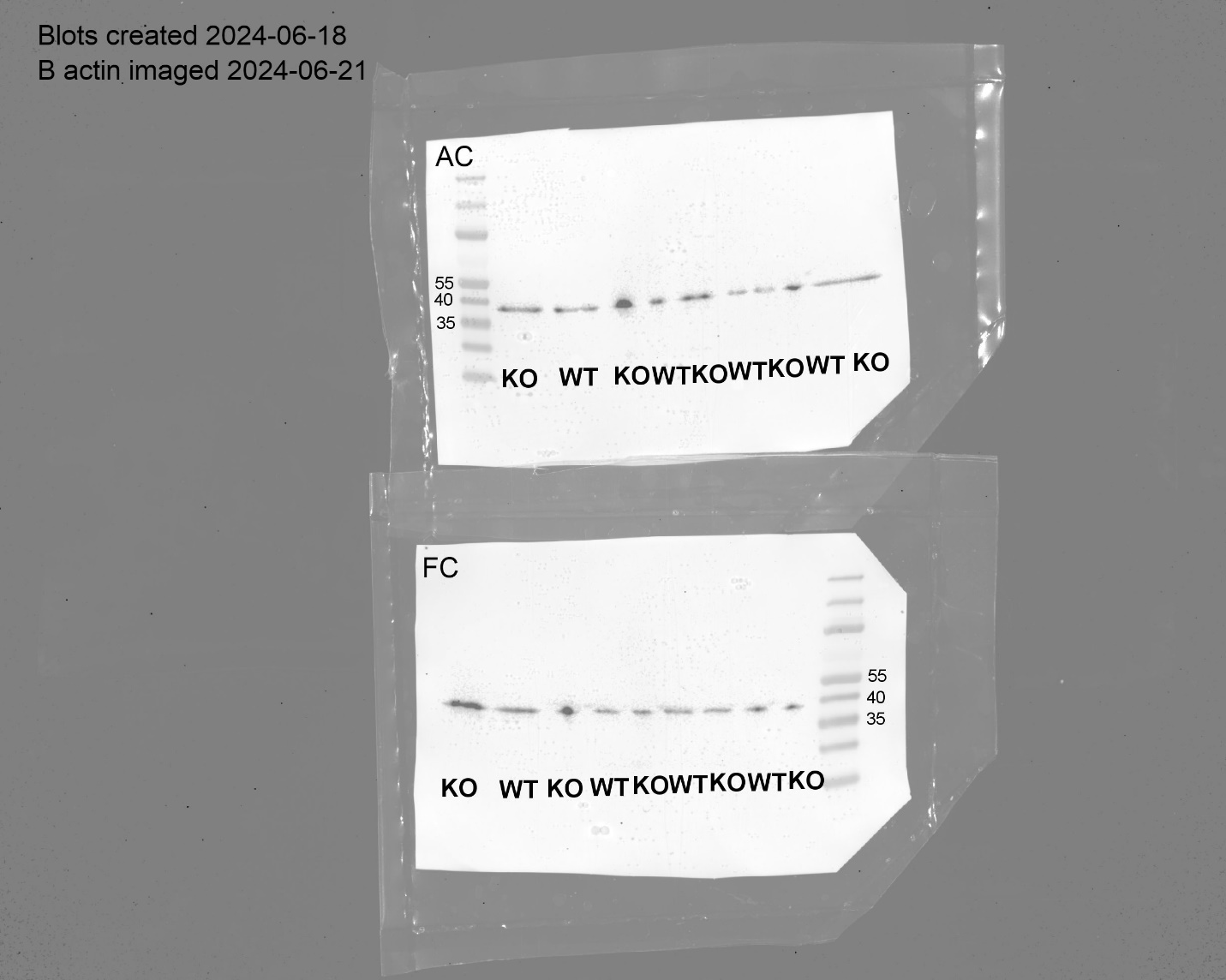


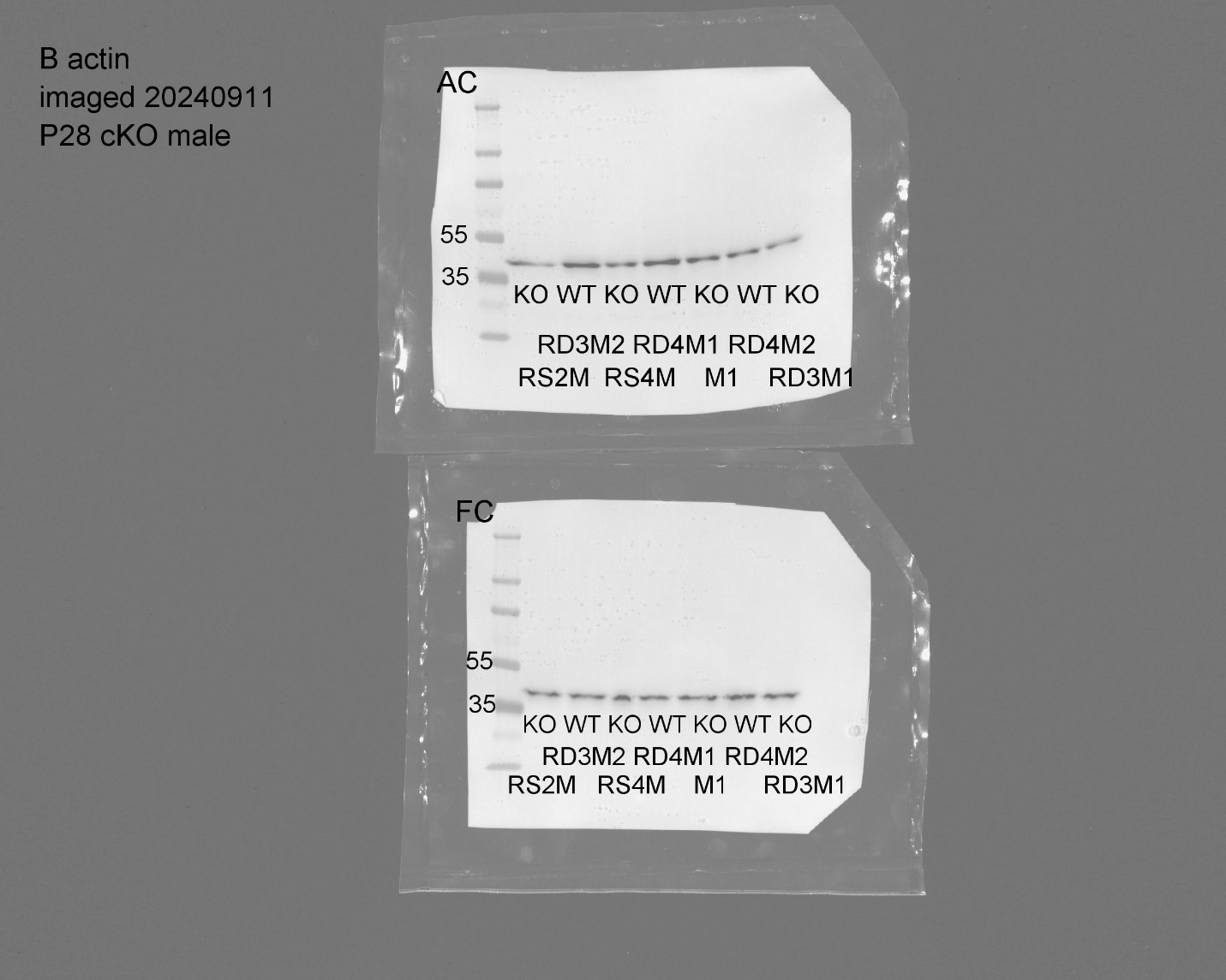

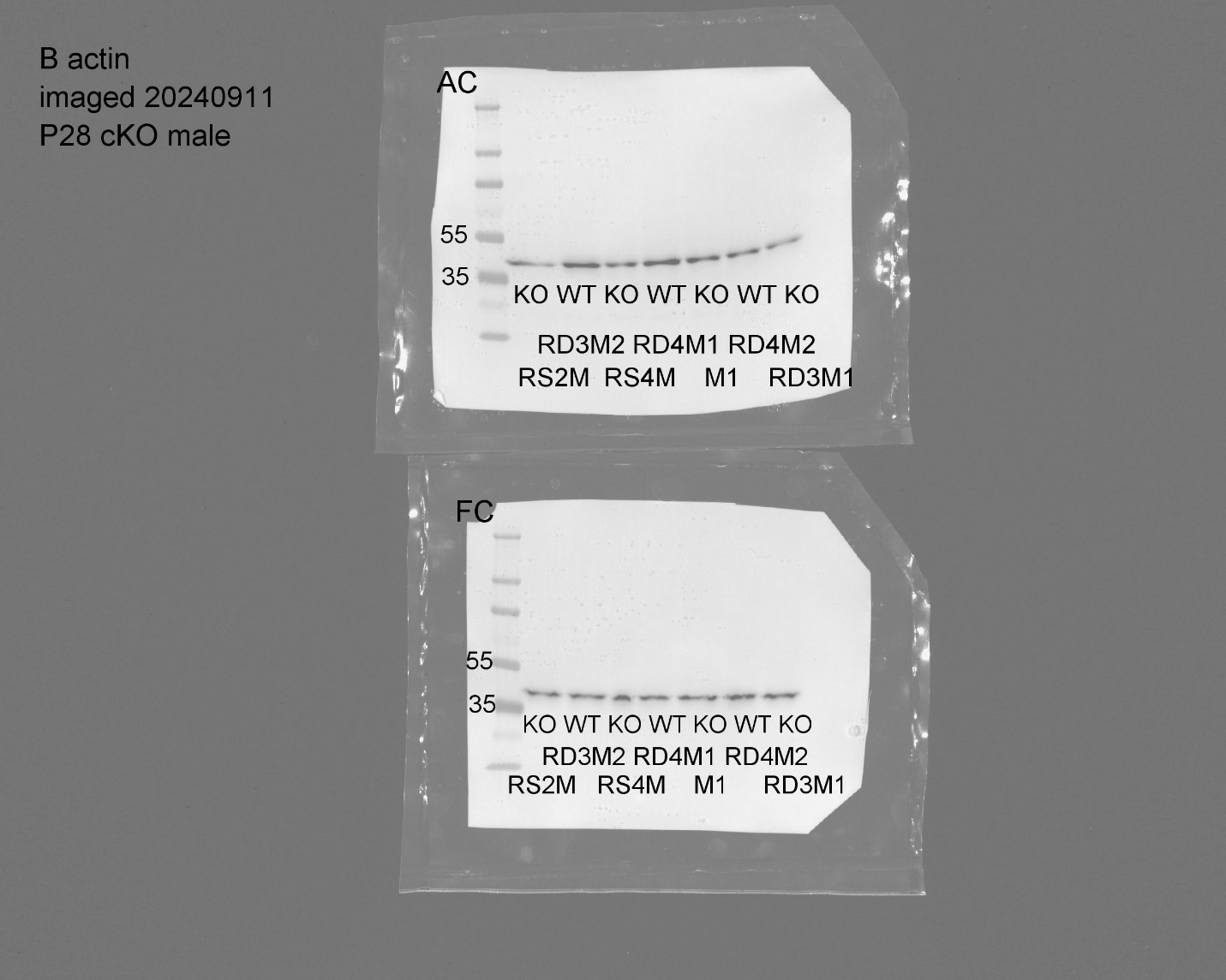


GABRa5 blots for Figure S2F(AuC first, FC second)


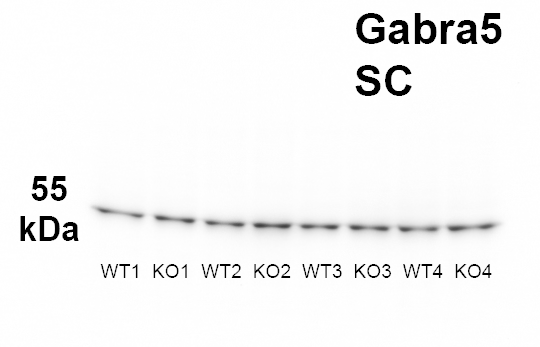


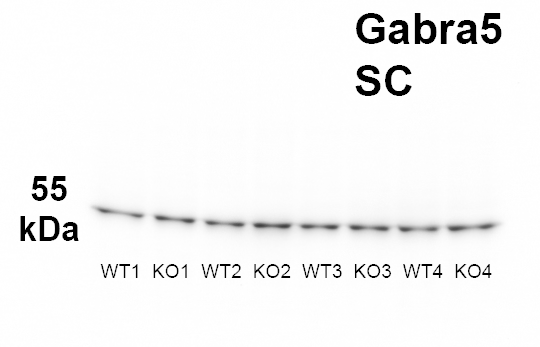


**

**

**

**

Beta-actin blots for Figure S2F (AuC first, FC second)


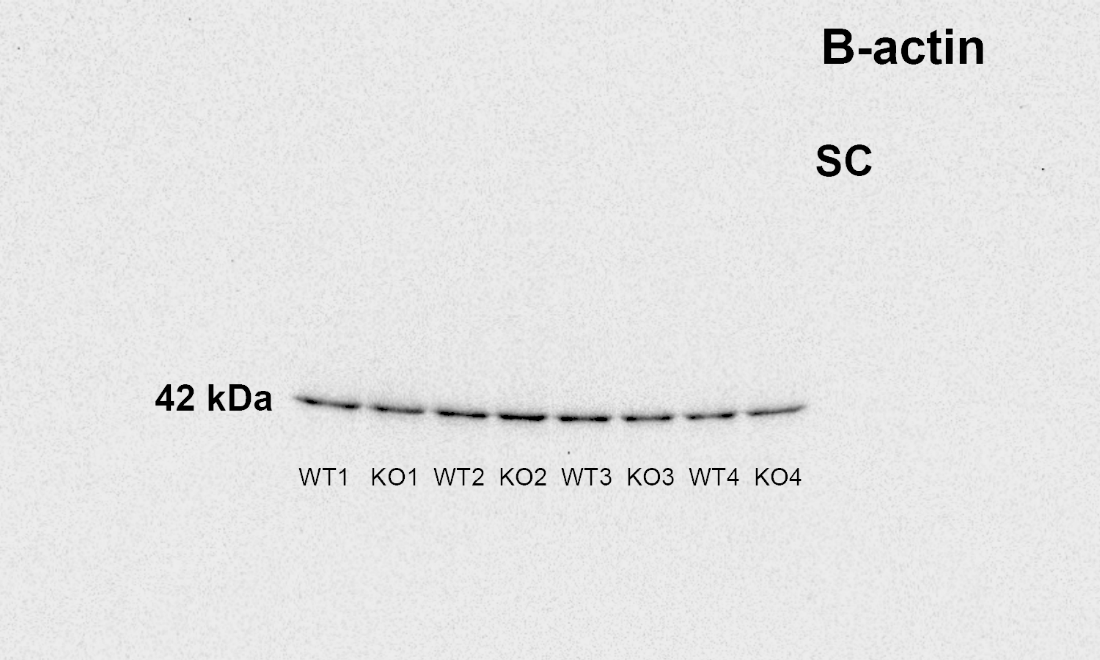


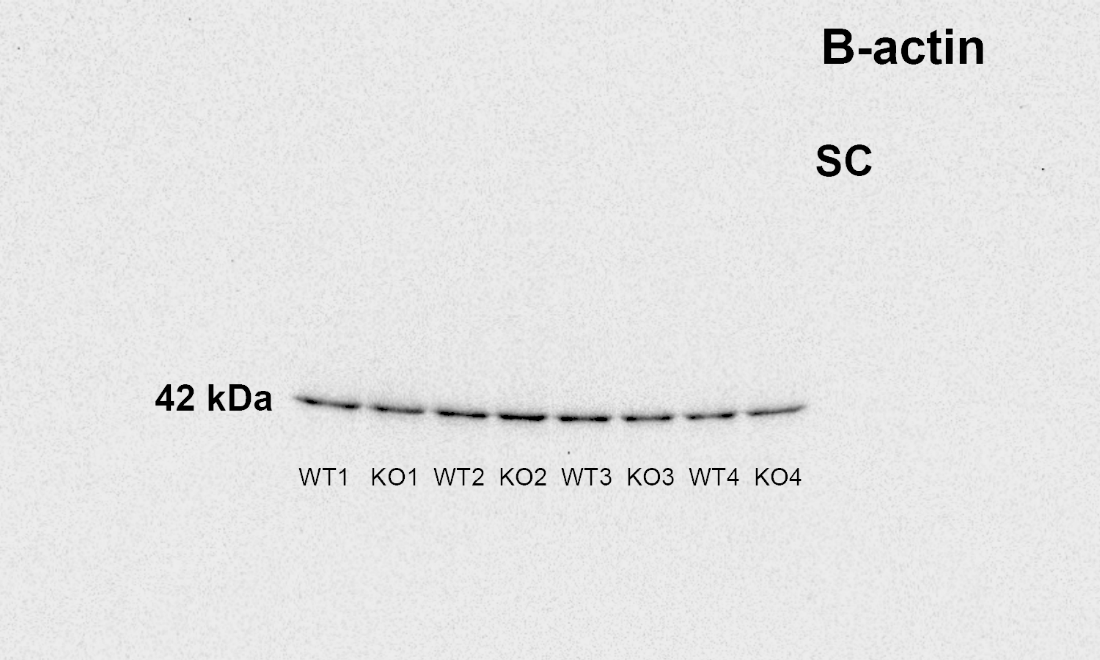


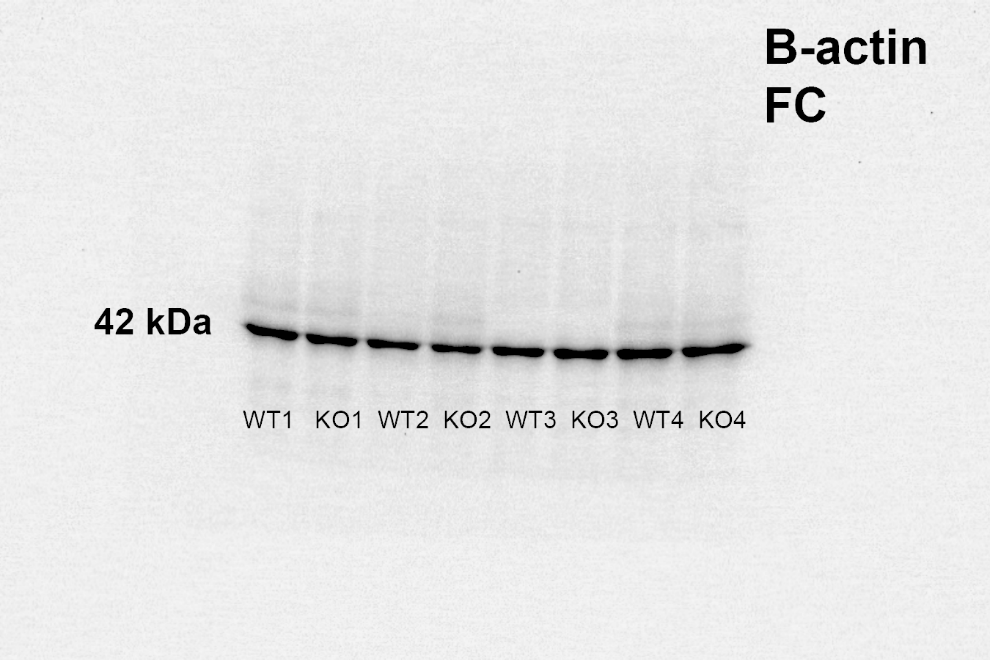


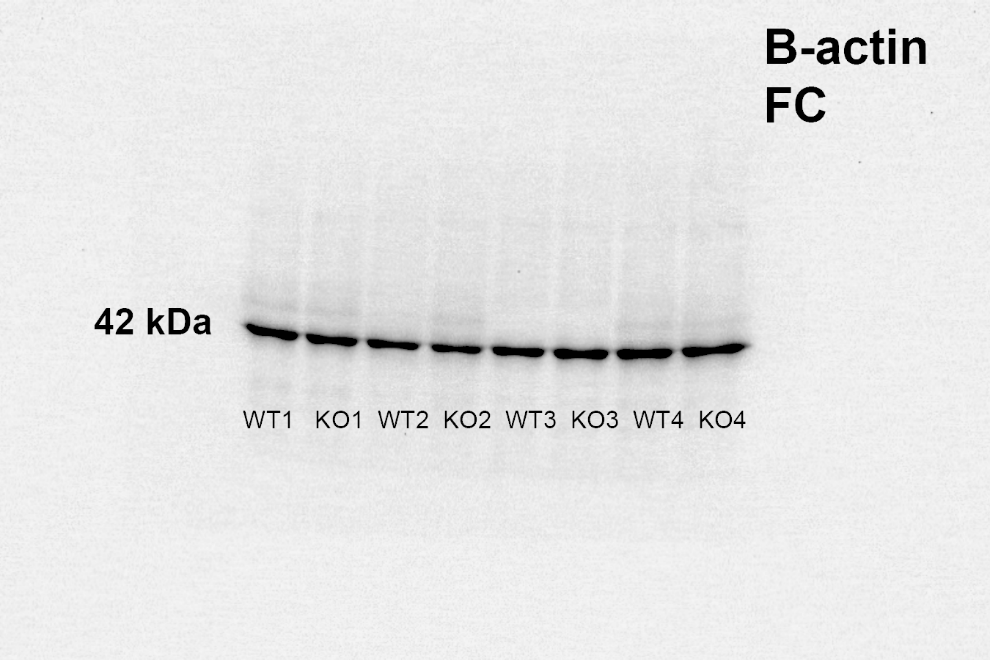

Supplement: 1 [file NIHMS2188793-supplement-1.docx]
